# Disease-specific tau polymorphs are associated with unique protein networks across proteinopathies

**DOI:** 10.1038/s44318-026-00897-8

**Published:** 2026-08-18

**Authors:** Nicha Puangmalai, Nemil Bhatt, Nopparat Suthprasertporn, Ernesto G Miranda-Morales, Nikita Shchankin, Madison Samples, Ahmet E Balci, Jia Yi Liew, Mauro Montalbano, Yingxin Zhao, Rakez Kayed

**Affiliations:** 1 Mitchell Center for Neurodegenerative Diseases, University of Texas Medical Branch, Galveston, TX USA; 2 Departments of Neurology, Neuroscience and Cell Biology, University of Texas Medical Branch, Galveston, TX USA; 3 Department of Internal Medicine, University of Texas Medical Branch, Galveston, TX USA; 4 Institute for Translational Sciences, University of Texas Medical Branch, Galveston, TX USA

**Keywords:** Molecular Biology of Disease, Neuroscience, Proteomics

## Abstract

Tau protein aggregates adopt distinct conformations across tauopathies, yet the protein interactions engaged by disease-specific polymorphs remain poorly characterized. Here, we demonstrate that conformationally distinct tau polymorphs associate with disease-specific interaction networks across Alzheimer’s disease (AD), progressive supranuclear palsy (PSP), and dementia with Lewy bodies (DLB). Interactome profiling of tau aggregates from PBS- and sarkosyl-soluble brain fractions identified 493 high-confidence interactors exhibiting remarkable disease specificity. As an exploratory feature discovery machine learning classification discriminated against diseases using as few as four to six protein features. AD tau selectively engaged glycolytic enzymes, TCA cycle components, and glutamate/GABA cycling machinery, with the astrocytic transporter SLC1A2 showing 27-fold enrichment. PSP tau exhibited extensive interactor depletion alongside selective proteasome enrichment, whereas DLB tau associated with neurogenesis modulators while depleting neuroinflammatory mediators. Interaction patterns were corroborated by parallel reaction monitoring mass spectrometry and proximity ligation assays and corresponded to disease-specific post-translational modification profiles. These findings show that tau polymorph conformations are associated with disease-specific interaction networks, providing molecular insight into tauopathy heterogeneity.

## Synopsis

Conformationally distinct tau aggregates arise across tauopathies, but the proteins they engage are unknown. This study links tau polymorphs from Alzheimer’s disease (AD), progressive supranuclear palsy (PSP), and dementia with Lewy bodies (DLB) to interaction networks, offering insight into disease heterogeneity.

- Interactome profiling of tau aggregates from PBS- and sarkosyl-soluble AD, PSP and DLB postmortem brain identifies 493 high-confidence interactors with pronounced disease specificity.
- AD-tau selectively engages glycolytic enzymes, TCA-cycle components and glutamate/GABA-cycling machinery, with the astrocytic glutamate transporter SLC1A2 enriched 27-fold.
- PSP-tau shows broad interactor depletion alongside selective proteasome enrichment, whereas DLB-tau associates with neurogenesis-modulators while depleting neuroinflammatory mediators.
- Interaction patterns are corroborated by parallel reaction monitoring mass spectrometry and proximity ligation assays, and correspond to disease-specific tau post-translational modification profiles.

## Abstract (web summary)

Interactome profiling of tau aggregates from postmortem brain of Alzheimer’s disease (AD), progressive supranuclear palsy (PSP), and dementia with Lewy bodies (DLB) provides new insights into disease heterogeneity.

## Graphical abstract

**Figure:**
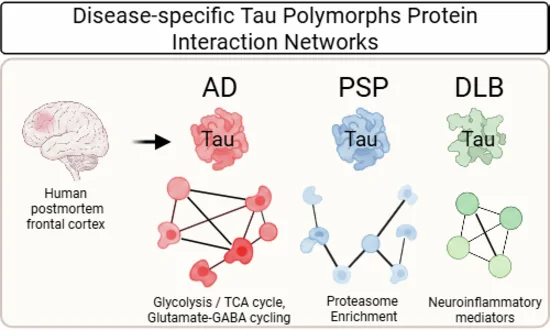

## Introduction

Pathological tau accumulation is strongly linked to neurodegeneration and cognitive decline across neurodegenerative diseases such as Alzheimer’s disease (AD), progressive supranuclear palsy (PSP), dementia with Lewy bodies (DLB), and other tauopathies (Chin et al, 2020; Creekmore et al, 2024; Ossenkoppele et al, 2022; Schumacher et al, 2021; Wu et al, 2022; Zheng et al, 2024). Notably, tau aggregates display remarkable structural and functional diversity between tauopathies, with distinct patterns of brain region distribution and cellular targets (Chung et al, 2021; Dujardin et al, 2018; He et al, 2020; Kaufman et al, 2016; Narasimhan et al, 2017; Vaquer-Alicea et al, 2021). PSP is characterized by neuronal and glial tau pathology, mainly involving 4R tau isoforms forming globose neurofibrillary tangles and tufted astrocytes (Dickson et al, 2010; Kovacs et al, 2020; Narasimhan et al, 2017). In AD, amyloid-β (Aβ) co-pathology synergistically accelerates tau-driven neurodegeneration (Busche and Hyman, 2020). DLB shows predominant α-synuclein (α-Syn) pathology in limbic and neocortical regions, with tau co-pathology present in over 70% cases and closely linked to cognitive impairment (Colom-Cadena et al, 2013; Irwin and Hurtig, 2018). Across these disorders, toxic soluble tau aggregates drive disease progression by promoting tau misfolding and aggregation (Hussong et al, 2023; Kopeikina et al, 2012; Wegmann et al, 2019). Recent evidence from our research highlights that tau oligomers isolated from postmortem human brains with AD, PSP, and DLB show distinct disease-specific polymorphs, with differences in structure, morphology, seeding ability, stability, and proteolytic digestion profiles, reflecting differential regulation of neuronal function and gene expression (Castillo-Carranza et al, 2014; Gerson et al, 2014; Lasagna-Reeves et al, 2010; Lasagna-Reeves et al, 2011; Lasagna-Reeves et al, 2012a; Lasagna-Reeves et al, 2012b; Lo Cascio et al, 2020; Lo Cascio et al, 2025; Puangmalai et al, 2020; Sengupta et al, 2015).

Beyond their aggregation, protein complexes are key molecular entities assembled into functional modules via protein–protein interactions (PPI) to execute core cellular functions (Gavin et al, 2006; Lin et al, 2015). These complexes prominently regulate essential processes, including DNA replication, transcription, translation, RNA splicing, protein secretion, cell cycle progression, signal transduction, and cellular metabolism (Lee and Yaffe, 2016; Yadav and Polasek-Sedlackova, 2024). PPIs are particularly crucial within cellular signaling networks; for instance, myriad cellular signals are transmitted through direct interactions between protein substrates and their post-translational modifying enzymes such as kinases/phosphatases, ubiquitinases/deubiquitinases, and acetyltransferases/deacetylases (Lee and Yaffe, 2016; Li et al, 2016; Mellert and McMahon, 2009; Wang and Wang, 2021). PPI analysis, therefore, yields meaningful insights into signal transduction mechanisms.

Recent studies underscore tau’s capacity to interact with varied protein partners, influencing signal transduction and diverse cellular processes (Drummond et al, 2020; Kavanagh et al, 2022; Tracy et al, 2022). However, it remains unknown if disease-specific tau oligomers possess distinct interactomes. To address this knowledge gap, we conducted a comprehensive investigation of the interactomes of PBS- and sarkosyl-soluble tau aggregates isolated from postmortem human brains with AD (AD tau), PSP (PSP tau), and DLB (DLB tau).

## Results

### Identification of disease-relevant misfolded tau interactomes

**Figure 1:**
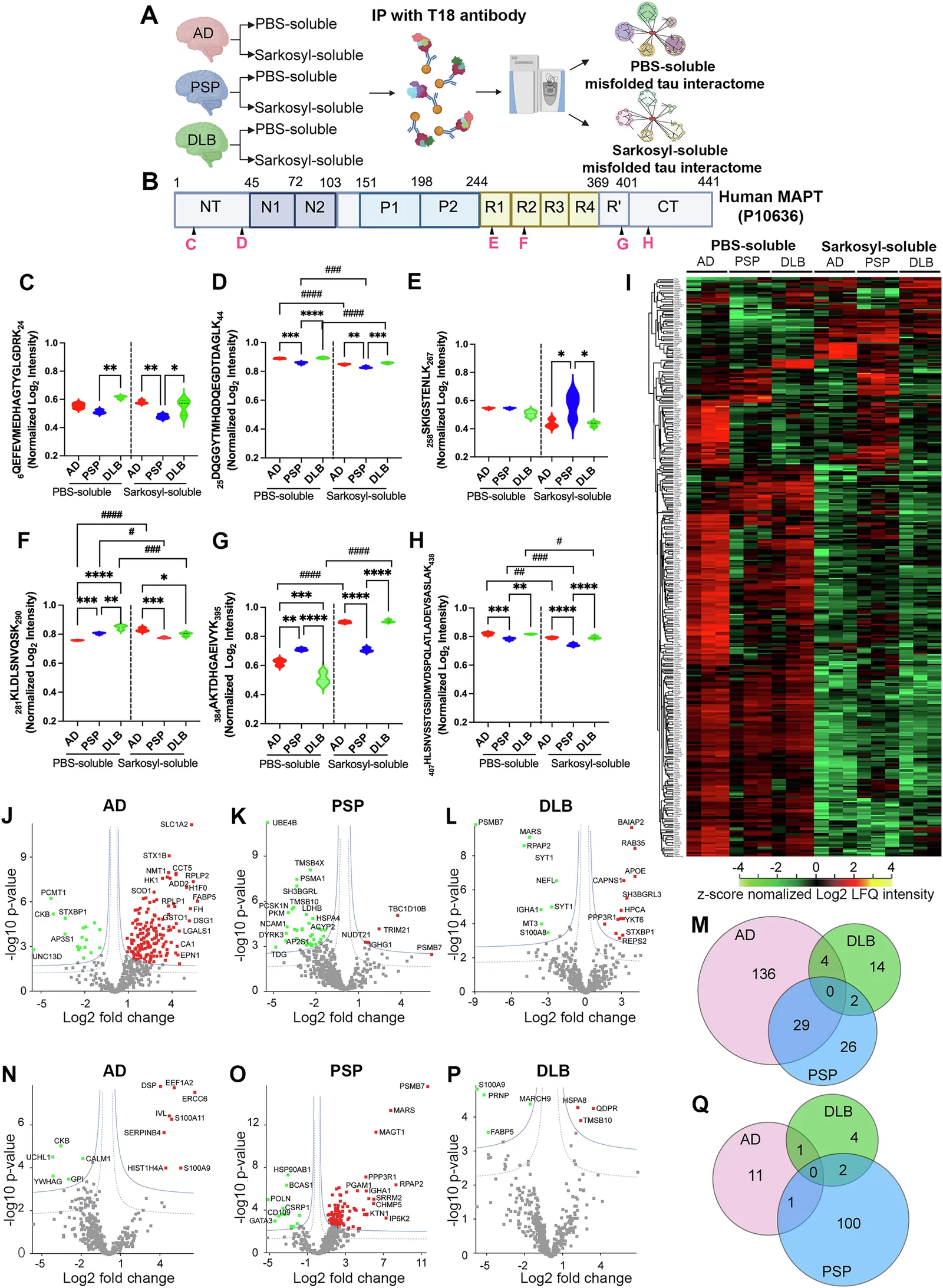
Identification of disease-specific PBS- and sarkosyl-soluble tau interactomes in postmortem brains. (**A**) Schematic workflow of the IP-MS approach used to isolate and quantify the disease-specific misfolded-tau interactome. Misfolded tau was immunoprecipitated from PBS-soluble and sarkosyl-soluble fractions of AD, PSP, and DLB human brain tissues using the conformation-specific T18 antibody, followed by LC-MS/MS (*n* = 3 independent cases per group). (**B**) Domain map of the longest human tau isoform (2N4R; UniProt P10636), detailing the N-terminal region (NT), inserts (N1, N2), proline-rich regions (P1 and P2), microtubule-binding repeats (R1–R4 and R′), and the C-terminus (CT). Magenta letters (**C**–**H**) map the precise locations of the representative tryptic peptides quantified below. (**C**–**H**) Quantitative profiles of representative misfolded-tau peptides captured in the pull-downs. Data were presented as violin plots showing the normalized log2 LFQ intensities in PBS- and sarkosyl-soluble fractions from AD (red), PSP (blue), and DLB (green) cases (*n* = 3 independent cases per group). *p < 0.05, **p < 0.01, ***p < 0.001, ****p < 0.0001 for pairwise differences across diseases within the same fraction; ^#^*p* < 0.05, ^##^*p* < 0.01, ^###^*p* < 0.001, ^####^*p* < 0.0001 for differences between PBS- and sarkosyl-soluble fractions within the same disease, determined by two-way ANOVA with Tukey’s post hoc test. The adjusted *p* values are as follows: (**C**) _**6**_QEFEVMEDHAGTYGLGDRK_24_; PBS-soluble PSP tau vs DLB tau (*p* = 0.0044), sarkosyl-soluble AD tau vs PSP tau (*p* = 0.0045), sarkosyl-soluble PSP tau vs DLB tau (*p* = 0.0463), (**D**) _25_DQGGYTMHQDQEGDTDAGLK_44_; PBS-soluble AD tau vs PSP tau (*p* = 0.0001), PBS-soluble PSP tau vs DLB tau (*p *< 0.0001), sarkosyl-soluble AD tau vs PSP tau (*p* = 0.0079), sarkosyl-soluble PSP tau vs DLB tau (*p* = 0.0002), PBS-soluble AD tau vs sarkosyl-soluble AD tau (*p* < 0.0001), PBS-soluble PSP tau vs sarkosyl-soluble PSP tau (*p* = 0.0001), PBS-soluble DLB tau vs sarkosyl-soluble DLB tau (*p* < 0.0001), (**E**) _258_SKIGSTENLK_267_; sarkosyl-soluble AD tau vs PSP tau (*p* = 0.0217), sarkosyl-soluble PSP tau vs DLB tau (*p* = 0.0188), (**F**) _281_KLDLSNVQSK_290_; PBS-soluble AD tau vs PSP tau (*p* = 0.0010), PBS-soluble PSP tau vs DLB tau (*p* = 0.0028), PBS-soluble AD tau vs DLB tau (*p* < 0.0001), sarkosyl-soluble AD tau vs PSP tau (*p* = 0.0003), sarkosyl-soluble AD tau vs DLB tau (*p* = 0.0263), PBS-soluble AD tau vs sarkosyl-soluble AD tau (*p* < 0.0001), PBS-soluble PSP tau vs sarkosyl-soluble PSP tau (*p* = 0.0319), PBS-soluble DLB tau vs sarkosyl-soluble DLB tau (*p* = 0.0009), (**G**) _384_AKTDHGAEIVYK_395_; PBS-soluble AD tau vs PSP tau (*p* = 0.0026), PBS-soluble PSP tau vs DLB tau (*p* < 0.0001), PBS-soluble AD tau vs DLB tau (*p* = 0.0004), sarkosyl-soluble AD tau vs PSP tau (*p* < 0.0001), sarkosyl-soluble PSP tau vs DLB tau (*p *< 0.0001), PBS-soluble AD tau vs sarkosyl-soluble AD tau (*p* < 0.0001), PBS-soluble DLB tau vs sarkosyl-soluble DLB tau (*p* < 0.0001), (**H**) _407_HLSNVSSTGSIDMVDSPQLATLADEVSASLAK_438_; PBS-soluble AD tau vs PSP tau (*p* = 0.0008), PBS-soluble PSP tau vs DLB tau (*p* = 0.0019), sarkosyl-soluble AD tau vs PSP tau (*p* < 0.0001), sarkosyl-soluble PSP tau vs DLB tau (*p* < 0.0001), PBS-soluble AD tau vs sarkosyl-soluble AD tau (*p* = 0.0061), PBS-soluble PSP tau vs sarkosyl-soluble PSP tau (*p* = 0.0002), PBS-soluble DLB tau vs sarkosyl-soluble DLB tau (*p* = 0.0229). (**I**) Hierarchical clustering heatmap of all significant proteins identified from the T18 immunoprecipitation (ANOVA with permutation-based FDR <5%). Data were displayed as z-score–normalized log2 LFQ intensities. (**J**–**L**) Volcano plots illustrating the statistical significance versus magnitude of change for proteins quantified from misfolded-tau pull-downs in the PBS-soluble fractions of AD, PSP, and DLB brains (*n* = 3 independent cases per group). Significant interactors were determined using a permutation-based false discovery rate (FDR). Solid and dashed blue lines denote high-confidence (Class A, 1% FDR) and lower-confidence (Class B, 5% FDR) thresholds, respectively. Red dots represent significantly enriched proteins (Class A, 1% FDR), green dots represent significantly depleted proteins (Class A, 1% FDR), and gray dots denote non-significant background proteins. (**M**) Venn diagrams detailing the intersection of significant misfolded-tau interacting proteins across the AD, PSP, and DLB cohorts in the PBS-soluble fractions. (**N**–**P**) Volcano plots illustrating the statistical significance versus magnitude of change for proteins quantified from misfolded-tau pull-downs in the sarkosyl-soluble fractions of AD, PSP, and DLB brains (*n* = 3 independent cases per group). Significant interactors were determined using a permutation-based false discovery rate (FDR). Solid and dashed blue lines denote high-confidence (Class A, 1% FDR) and lower-confidence (Class B, 5% FDR) thresholds, respectively. Red dots represent significantly enriched proteins (Class A, 1% FDR), green dots represent significantly depleted proteins (Class A, 1% FDR), and gray dots denote non-significant background proteins. (**Q**) Venn diagrams detailing the intersection of significant misfolded-tau interacting proteins across the AD, PSP, and DLB cohorts in the sarkosyl-soluble fractions. Source data are available online for this figure.

Our previous studies have demonstrated that tau oligomers from PBS- and sarkosyl-soluble fractions significantly differ in post-translational modifications, morphology, and function (Lo Cascio et al, 2025; Puangmalai et al, 2022). Therefore, we investigated the interactome of toxic tau aggregates from the two fractions separately. To do so, tau aggregates and their interacting proteins from the two fractions of AD, PSP, and DLB human brain homogenates (*n* = 3 cases, Table 1A) were isolated using co-immunoprecipitation (co-IP) with T18 antibody, which is specific for misfolded tau aggregates (Arar et al, 2025; Kadamangudi et al, 2024; Lo Cascio et al, 2020; Lo Cascio et al, 2025; Marcatti et al, 2025; Puangmalai et al, 2024; Puangmalai et al, 2022; Ruan et al, 2021; Samelson et al, 2026; Scaduto et al, 2024) (Fig. 1A). The proteins were then identified and quantified using label-free LC-MS, as described previously (Zhang et al, 2017). A separate cohort of postmortem brain tissues from control, AD, PSP, and DLB (*n* = 5 cases, Table 1B) were used for validation.

**Table 1:** Summary of human brain tissues analysed in this study.

Proteomic study type	Pathology/ Brain #	Sex	Age	Brain area	PMI (h)	Braak stage
A: Tau interactome	AD1	F	70	frontal cortex	3.25	6
	AD2	M	71	frontal cortex	11	6
	AD3	F	76	frontal cortex	11	6
	PSP1	M	85	frontal cortex	7	NA
	PSP2	M	77	frontal cortex	12	NA
	PSP3	F	71	frontal cortex	18	NA
	DLB1	M	81	frontal cortex	4	NA
	DLB2	M	72	frontal cortex	18	NA
	DLB3	F	76	frontal cortex	10	NA
B: Tau interactome validation	Control 1	M	71	frontal cortex	2	1
	Control 2	M	79	frontal cortex	NA	NA
	Control 3	M	78	frontal cortex	1.17	2
	Control 4	M	84	frontal cortex	1.5	2
	Control 5	F	75	frontal cortex	3.5	1
	AD1	M	77	frontal cortex	7	6
	AD2	M	78	frontal cortex	5	6
	AD3	M	89	frontal cortex	10	6
	AD4	F	80	frontal cortex	2.3	6
	AD5	F	87	frontal cortex	5	6
	PSP1	M	66	frontal cortex	5	7
	PSP2	F	84	frontal cortex	14	4
	PSP3	M	80	frontal cortex	2.66	3
	PSP 4	M	85	frontal cortex	4.75	5
	PSP 5	F	≥90	frontal cortex	4	3
	DLB1	M	71	frontal cortex	6	5
	DLB2	F	≥90	frontal cortex	3.47	4
	DLB3	M	85	frontal cortex	3.5	5
	DLB 4	M	82	frontal cortex	4.58	3
	DLB 5	F	>89	frontal cortex	3	NA

**Figure EV1:**
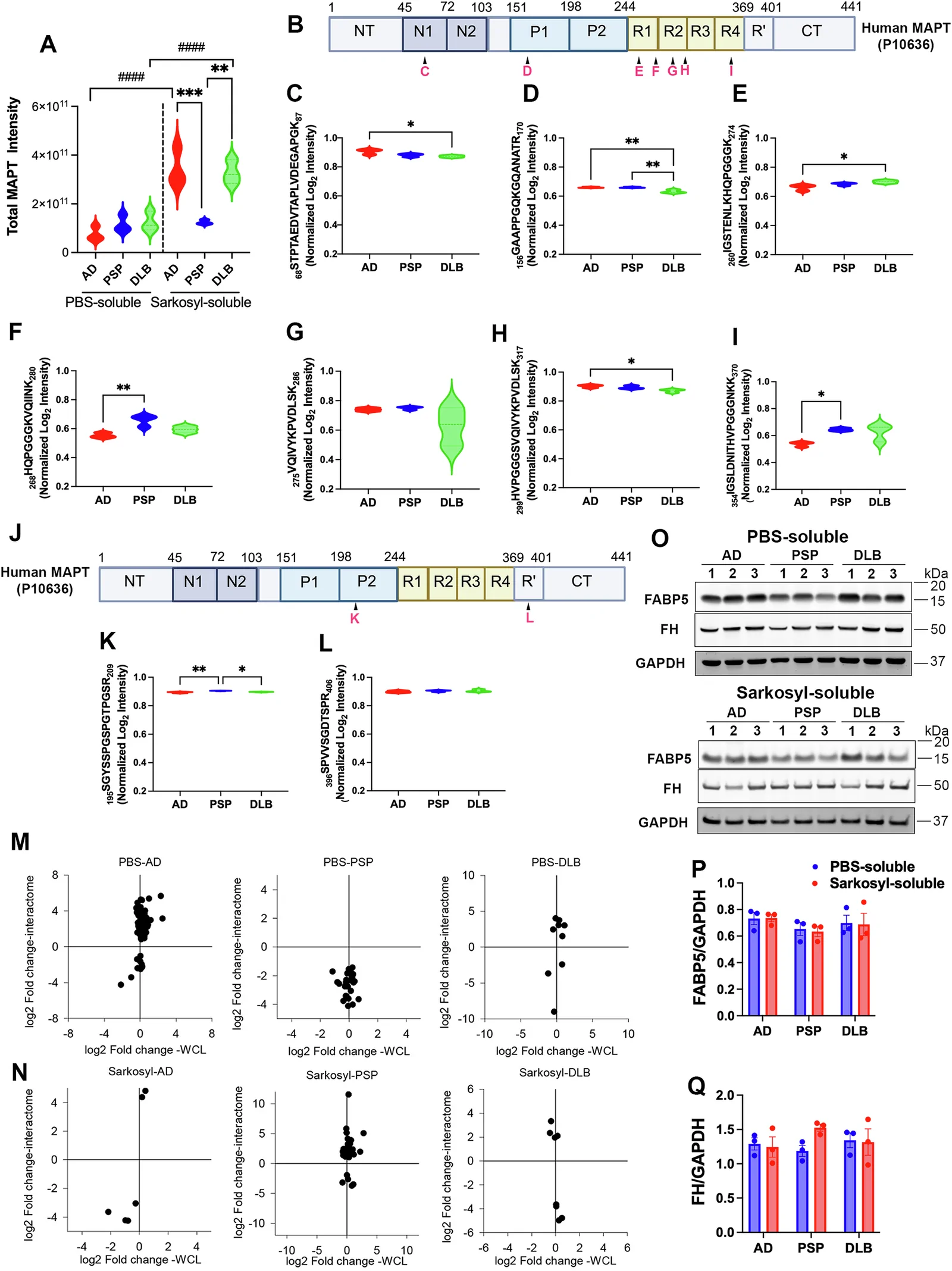
Related to Fig. 1. Comparison of tau interactomes with the brain proteome and validation of selected proteins. (**A**) Total MAPT signal intensity across PBS-soluble and sarkosyl-soluble fractions in AD (red), PSP (blue), and DLB (green) cases (*n* = 3 independent cases per group) was detected by label-free LC-MS/MS. The adjusted *p* values are as follows: PBS-soluble AD tau vs sarkosyl-soluble AD tau (*p* < 0.0001), PBS-soluble DLB tau vs sarkosyl-soluble DLB tau (*p* < 0.0001), sarkosyl-soluble AD tau vs PSP tau (*p* = 0.0006), sarkosyl-soluble PSP tau vs DLB tau (*p* = 0.0012). (**B**) Domain map of the full-length human MAPT with magenta letters marking the positions of representative tryptic peptides plotted for PBS-soluble tau (**C**–**I**). (**C**–**I**) Normalized Log2 LFQ intensities for representative tau peptides from PBS-soluble fractions across AD, PSP, and DLB (*n* = 3 independent cases per group). The adjusted *p* values are as follows: (**C**) _68_STPTAEDVTAPLVDEGAPGK_87_; AD tau vs DLB tau (*p* = 0.0406), (**D**) _156_GAAPPGQKGQANATR_170_; AD tau vs DLB tau (*p* = 0.0070), PSP tau vs DLB tau (*p* = 0.0070), (**E**) _260_IGSTENLKHQPGGGK_274_; AD tau vs DLB tau (*p* = 0.0331), (**F**) _268_HQPGGGKVQIINK_280_; AD tau vs PSP tau (*p* = 0.0094), (**H**) _299_HVPGGGSVQIVYKPVDLSK_317_; AD tau vs DLB tau (*p* = 0.0306), (**I**) _354_IGSLDNITHVPGGGNKK_370_; AD tau vs PSP tau (*p* = 0.0317). (**J**) MAPT domain map with peptide positions for sarkosyl-soluble tau (**K**, **L**). (**K**,** L**) Normalized Log2 LFQ intensities for representative tau peptides from sarkosyl-soluble fractions across AD, PSP, and DLB (*n* = 3 independent cases per group). The adjusted *p* values are as follows: (**K**) _195_SGYSSPGSPGTPGSR_209_; AD tau vs PSP tau (*p* = 0.0059), PSP tau vs DLB tau (*p* = 0.0244). (**M**) Scatter plots comparing Log2 fold change (FC) in the tau interactome (y-axis) versus Log2 FC in total PBS-soluble lysate (x-axis) for PBS-soluble fractions from AD, PSP, and DLB brains. Each point is a protein significantly detected in T18 immunoprecipitations; gray crosshairs mark zero on both axes. (**N**) Scatter plots comparing Log2 FC in the misfolded tau interactome (y-axis) versus Log2 FC in total sarkosyl-soluble lysate (x-axis) for sarkosyl-soluble fractions from AD, PSP, and DLB brains. Each point is a protein significantly detected in T18 immunoprecipitations; gray crosshairs mark zero on both axes. (**O**–**Q**) Representative immunoblots and quantification of FABP5 and FH normalized to GAPDH in total PBS-soluble (top) and sarkosyl-soluble (bottom) from AD, PSP, and DLB cases (*n* = 3). Blue dots, PBS-soluble; red dots, sarkosyl-soluble. Quantification of band intensity was normalized to GAPDH (mean ± SEM). The same immunoblots probed with loading controls are shown in Figs. 6. and EV3. Statistical analyses were calculated by two-way ANOVA with the Tukey test. Asterisks indicate pairwise differences among diseases within a fraction; hash marks indicate PBS- vs sarkosyl-soluble differences within the same disease (*p < 0.05, **p < 0.01, ***p < 0.001, ****p < 0.0001; ^#^*p* < 0.05, ^##^*p* < 0.01, ^###^*p* < 0.001, ^####^*p* < 0.0001).Source data are available online for this figure.

First, we investigated whether misfolded tau aggregates in AD, PSP, and DLB differ in their susceptibility to trypsin digestion. LC-MS/MS analysis of the tryptic digests of misfolded tau aggregates pulled down from the brains of AD, PSP, and DLB identified 39 tau peptides. To demonstrate the variation in peptide abundance across different tau domains, we normalized the intensity of each peptide with total tau intensity (Fig. EV1A). Figure 1C–H show the normalized intensities of peptides mapped to tau domains. Statistical analysis revealed significant differences in intensity of tau peptides between the diseases as well as tau aggregates with different solubility (PBS- vs sarkosyl-soluble), including the tau fragments from the N-terminal region (NT) (Fig. 1B–D), microtube-binding repeats R1 (Fig. 1B,E), R2 (Fig. 1B,F), R’ (Fig. 1B,G), and C-terminal region (Fig. 1B,H). Additionally, as illustrated in the MAPT domain mapping (Fig. EV1B), we identify seven tau peptides from the PBS-soluble-enriched fraction (Fig. EV1C–I) that are mainly located in microtubule-binding regions (R1–R4), and two tau peptides from the sarkosyl-soluble-enriched fraction (Fig. EV1J–L) on proline-rich (P2) and R’ regions. Together, trypsin digestion coupled with LC-MS/MS analysis demonstrated disease-specific differences in tau conformation in AD, PSP, and DLB brains, as evidenced by differential cleavage site accessibility.

Next, we investigated the disease-specific tau interactome. Proteomic analysis of tau pull-down identified a total of 493 proteins with high confidence (false discovery rate (FDR) <1%) (Dataset EV1). Since the abundance of tau varies in each sample, the intensity of each protein was normalized using total tau. Then, multiple ANOVA test with permutation-based FDR was used to identify significant proteins among groups, revealing that 317 proteins exhibited group-wise differences (FDR <5%) (Fig. 1I; Dataset EV2). The heatmap of these 317 proteins displays the intensity of the proteins in each sample, with proteins in red being enriched in each tau aggregate pull-down and proteins in green being depleted from tau aggregates, compared to other groups. As shown in Fig. 1I, significant differences in the misfolded tau interactome are present not only among diseases, but also between the two fractions (PBS vs sarkosyl-soluble). Hawaii plot analysis (multi-volcano plots) with the complement negative control was used to identify the disease-specific misfolded tau interactome (Rudolph and Cox, 2019) (Fig. 1J–P). Different s0 and permutation-based FDR parameters were used in the multi-volcano plot analysis to define class A (higher confidence, s0 = 1, FDR = 1%) and class B (lower confidence, s0 = 1, FDR = 5%) interactors. Each volcano plot displays the results of a pull-down of PBS- or sarkosyl-soluble misfolded tau aggregates from AD (Fig. 1J,N), PSP (Fig. 1K,O), and DLB (Fig. 1L,P), respectively. The thresholds for Class A significant interactors (blue solid lines) and Class B significant interactors (blue dashed lines) are shown in the plot. Class A proteins that were significantly enriched or depleted from PBS-soluble AD tau aggregates, relative to PBS-soluble DLB and PSP tau aggregates, are highlighted in red and green, respectively, while those in gray represent non-specific binding proteins. Only Class A interactors were used for the downstream protein interaction network and bioinformatics analysis. In the PBS-soluble fraction, we identified significant Class A interactors, of which 169 were (151 enriched and 18 depleted) from AD (Fig. 1J), 57 (5 enriched and 52 depleted) from PSP (Fig. 1K), and 20 (12 enriched and 8 depleted) from DLB (Fig. 1L), respectively. Similarly, we identified 13 (8 enriched and 5 depleted), 103 (91 enriched and 12 depleted), and 7 (4 enriched and 3 depleted) significant proteins from the AD (Fig. 1N), PSP (Fig. 1O), and DLB (Fig. 1P) pull-downs in the sarkosyl-soluble fraction. Following Hawaii plot analysis for the visualization of the tau interactome profile, Venn diagrams were generated to quantify misfolded tau interactors within the disease-specific interactors and to identify the common interactors among AD, PSP, and DLB across both PBS-soluble (Fig. 1M; Dataset EV3) and sarkosyl-soluble fractions (Fig. 1Q; Dataset EV4). These results suggest the distinct tau conformation between AD, PSP, and DLB may influence the misfolded tau interacting partners, resulting in diverse pathological phenotypes among the three diseases.

To determine whether the significant disease-specific enrichment or depletion observed in misfolded tau pull-downs was caused by the differential expression levels of these proteins in the brains of patients with AD, PSP, and DLB, we conducted a proteomics analysis of the whole lysate of the brain tissues. We compared the results with those from the misfolded tau pull-down experiments. For example, among the 169 significant proteins identified in the PBS-soluble AD tau pull-down, 119 were also quantified in the proteome study. As shown in Fig. EV1M, there is no strong correlation between the disease-specific changes of these proteins in PBS-soluble tau pull-downs and their differential expression in the brain tissues, like the sarkosyl-soluble tau pull-downs (Fig. EV1N). Similarly, there is no strong correlation between the PSP-or DLB-specific changes of tau interactors and their differential expression in the brain tissues (Fig. EV1M,N; Dataset EV5). Western blot (WB) validation of the representative tau interactors related to the TCA cycle (FH) and glucose metabolic process (FABP5) shows that their expression levels were not significantly different in the brains of AD, PSP, and DLB, or between the two fractions. (Fig. EV1O–Q). Together, these results suggested that the significant changes observed in misfolded tau pull-downs were caused by alterations in protein–protein interactions, rather than differential expression levels in the brains of patients with AD, PSP, and DLB.

### Disease-specific PBS-soluble tau interactomes reveal distinct functional signatures

**Figure 2:**
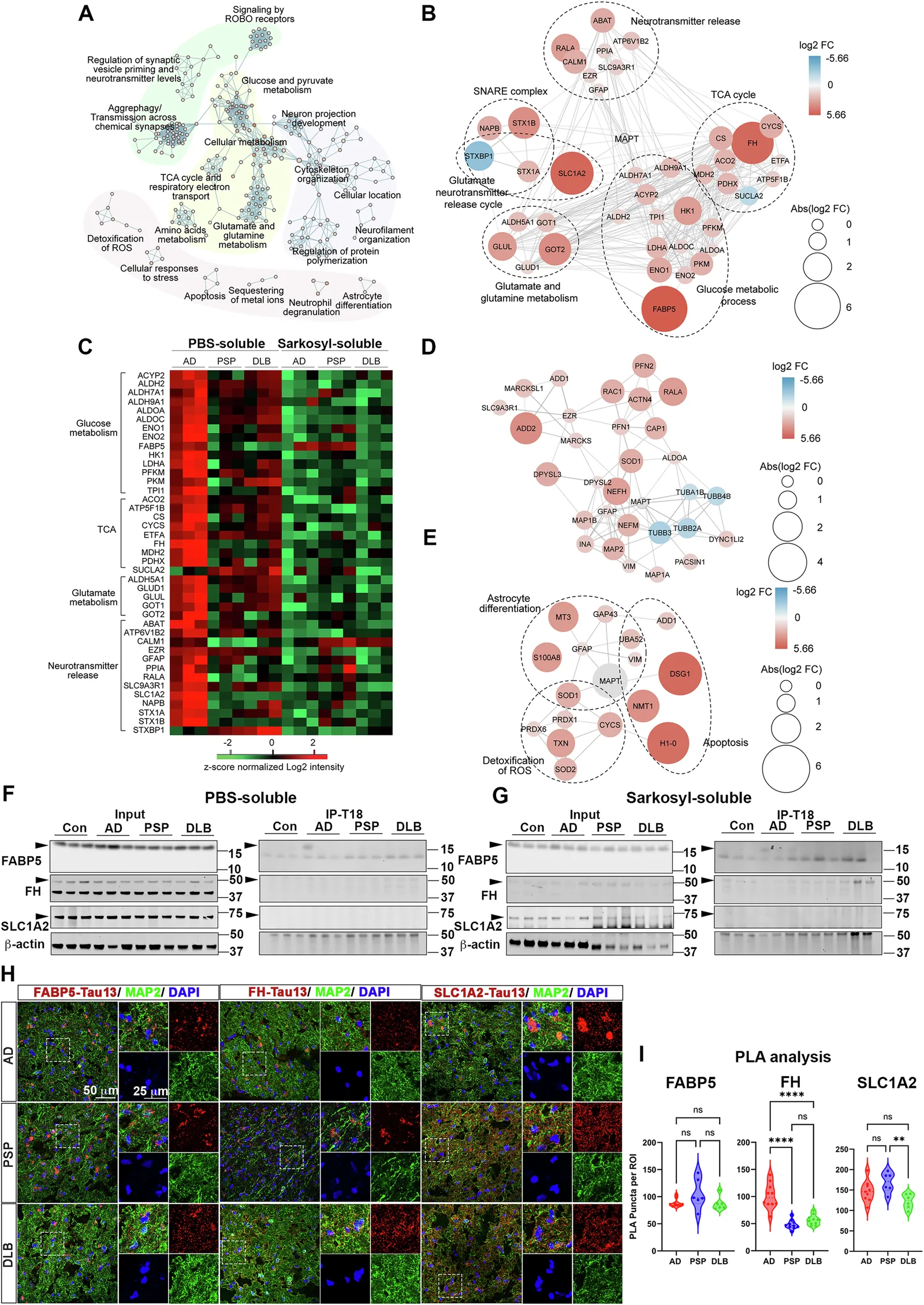
Characterization of PBS- and sarkosyl-soluble AD tau interactome. (**A**) Functional landscape of the PBS-soluble tau interactome. The network map highlights enriched biological processes among the 169 proteins significantly enriched with, or depleted from, misfolded tau immunoprecipitated from PBS-soluble AD brain extracts. (**B**) Sub-network analysis of significant AD tau interactors associated with glucose metabolism, the TCA cycle, glutamine and glutamate metabolism, and neurotransmitter release (including the SNARE complex). Node color indicates the log2 fold change (FC) relative to complement controls (red: enriched in AD; blue: depleted in AD), and node size reflects the absolute magnitude. (**C**) Heatmap displaying the relative abundance of the significant interactors highlighted in (**B**). Data were shown as z-score–normalized log2 LFQ intensities across both PBS- and sarkosyl-soluble fractions from AD, PSP, and DLB (ANOVA with permutation-based FDR <5%). (**D**,** E**) Sub-networks detailing significant AD tau interactors implicated in (**D**) cytoskeleton organization and (**E**) reactive oxygen species (ROS) detoxification, astrocyte differentiation, and apoptosis. Node color indicates the log2 FC relative to complement controls (red: enriched in AD; blue: depleted in AD), and node size reflects the absolute magnitude. (**F**,** G**) Biochemical validation of selected interactors. Representative WB of inputs and misfolded-tau (T18) immunoprecipitates (IP-T18) from (**F**) PBS-soluble and (**G**) sarkosyl-soluble fractions of Control (Con), AD, PSP, and DLB brain extracts. Blots were probed for FABP5, FH, and SLC1A2. β-actin serves as a loading control. All the protein of interests probed for here have been detected on the same, re-probed membrane for marker proteins in Figs. 3 and 6. (**H**) Validation of in situ protein interactions via PLA. Representative confocal micrographs showing PLA signals (red) for FABP5–Tau13, FH–Tau13, and SLC1A2–Tau13 interactions in AD, PSP, and DLB brain sections. Neurons are counterstained with MAP2 (green) and nuclei with DAPI (blue). Scale bars: 50 μm (main panels) and 25 μm (magnified insets). (**I**) Quantification of PLA puncta per region of interest (ROI) for the interactions shown in (H). Data were presented as violin plots showing the data distribution (*n* = 3 independent cases per group). *p < 0.05, **p < 0.01, ****p < 0.0001, ns not significant, determined by one-way ANOVA followed by Tukey’s post hoc test. The adjusted *p* values are as follows: FH; AD vs PSP (*p* < 0.0001), AD vs DLB (*p* < 0.0001), SLC1A2; PSP vs DLB (*p* = 0.0087). Source data are available online for this figure.

**Figure EV2:**
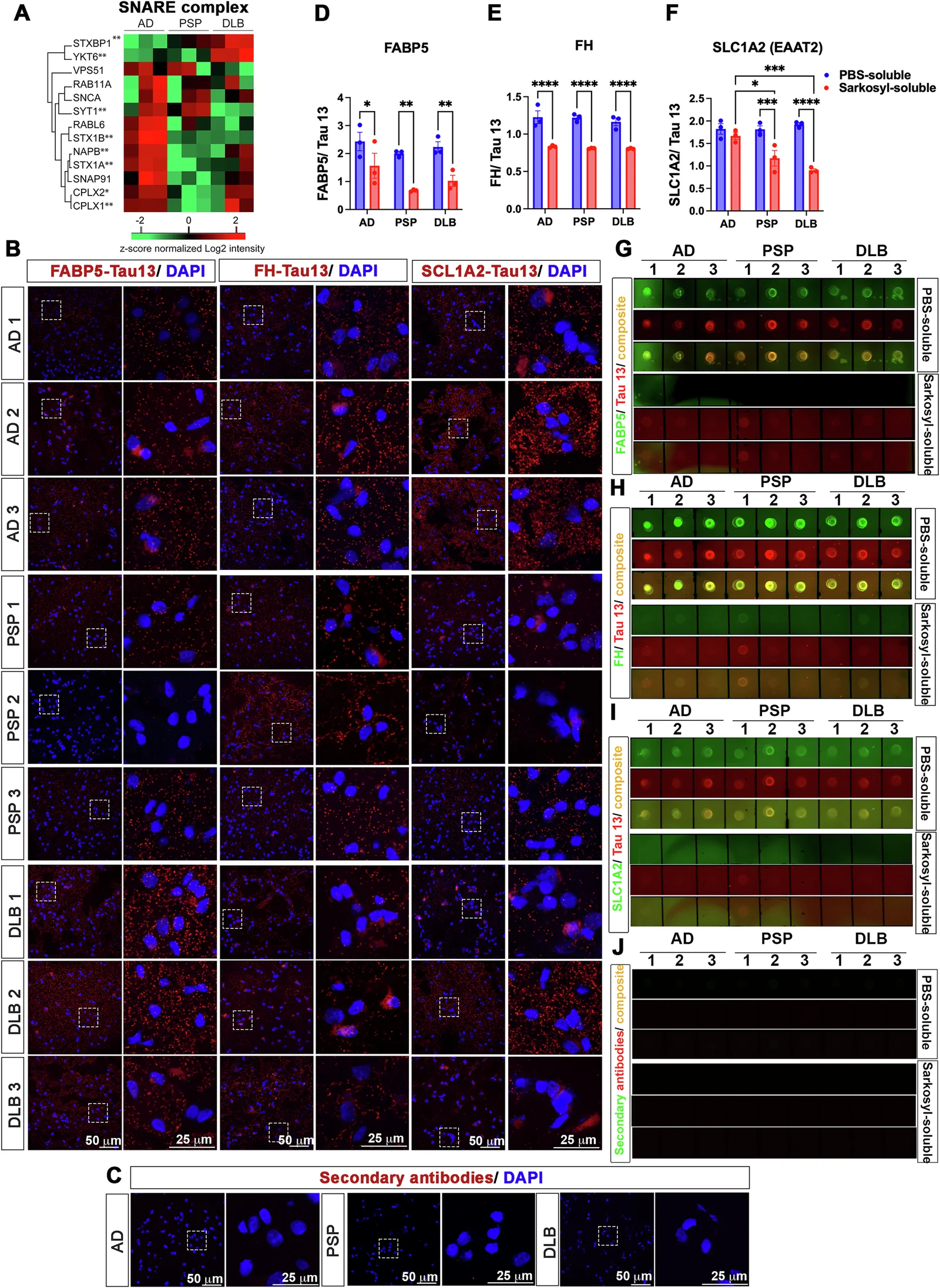
Disease-specifi c tau inteact with SNARE complex and biochemical validation of selected interactors. Related to Fig. 2. (**A**) SNARE complex interacts with disease-relevant misfolded tau. Heatmap of proteins involved in the SNARE complex. **, Class A interactor (*q* < 0.01); *, Class B interactor (*q* < 0.05) (ANOVA with permutation-based FDR <5%). Interactome analysis identified 13 SNARE complex proteins, with 9 showing disease-specific interaction patterns. STXBP1 and YKT6 were enriched with DLB-derived tau, while SYT1 was depleted from DLB tau. Several SNARE proteins, including STX1A, STX1B, and NAPB, significantly interacted with AD tau, but no significant SNARE enrichment was observed with PSP tau. CPLX1 and CPLX2 were significantly depleted from PSP-derived tau compared to AD- or DLB tau. These results highlight disease-specific tau aggregate interactions with SNARE components involved in synaptic vesicle function. (**B**) PLA images showing Tau13 (total tau) in proximity with FABP5, FH, and SLC1A2 in postmortem cortex from AD (AD1–3), PSP (PSP1–3), and DLB (DLB1–3). Boxed regions are magnified at right. Scale bars: 50 μm (left), 25 μm (right). (**C**) Control immunostaining for secondary antibodies shows no background signal across disease groups (AD, PSP, DLB). (**D**–**J**) Dual-color dot blot assays of IP materials with T18 antibody (misfolded tau) from PBS- and sarkosyl-soluble fractions probed with Tau13 and antibodies against (**D**,** G**) FABP5, (**E**,** H**) FH, and (**F**,** I**) SLC1A2 in AD, PSP, and DLB samples (*n* = 3 independent cases per group). Blue dots, PBS-soluble; red dots, sarkosyl-soluble. Data presented as mean ± SEM. *p < 0.05, **p < 0.01, ****p < 0.0001 by one-way ANOVA with Tukey test. The adjusted *p* values are as follows: (**D**) FABP5; PBS-soluble vs sarkosyl-soluble AD tau (*p* = 0.0331), PBS-soluble vs sarkosyl-soluble PSP tau (*p* = 0.0032), PBS-soluble vs sarkosyl-soluble DLB tau (*p* = 0.0058), (**E**) FH; PBS-soluble vs sarkosyl-soluble AD tau (*p* < 0.0001), PBS-soluble vs sarkosyl-soluble PSP tau (*p* < 0.0001), PBS-soluble vs sarkosyl-soluble DLB tau (*p* < 0.0001), (**F**) SLC1A2 (EAAT2); sarkosyl-soluble AD tau vs PSP tau (*p* = 0.0101), sarkosyl-soluble AD tau vs DLB tau (*p* = 0.0004), PBS-soluble vs sarkosyl-soluble PSP tau (*p *= 0.0006), PBS-soluble vs sarkosyl-soluble DLB tau (*p* < 0.0001). (**J**) Dot blot negative control (secondary antibodies only) for PBS- and sarkosyl-soluble IP materials with T18 antibody from AD, PSP, and DLB showing no detectable signal.Source data are available online for this figure.

Following Hawaii plot analysis of PBS-soluble proteins, functional enrichment and network annotation using Cytoscape StringApp (Doncheva et al, 2019) and EnrichmentMap tool (Betters et al, 2023; Kavanagh et al, 2022) revealed that the 169 significant proteins are predominantly associated with cellular metabolism, synaptic function, neuron development, and inflammatory response (Fig. 2A). Clusters enriched in glucose metabolism, TCA cycle, and respiratory electron transport processes were detected among PBS-soluble AD tau aggregates, with network analysis (Figs. 2B and EV2A) highlighting co-immunoprecipitation of key glycolytic enzymes (HK1, PKM, and PFKM) and mitochondrial proteins (PDHX, CS, ACO2, MDH2, FH, ETFA, and CYCS) with PBS-soluble AD tau aggregates. These findings underscore strong links between AD tau pathology and early metabolic dysfunction (Cheng and Bai, 2018; Isei et al, 2024; Liu et al, 2009; Qu et al, 2023).

Heatmap analysis further revealed group-wise differences (FDR <5%) with key enzymes of the glutamate/GABA-glutamine cycle, including GOT1, GOT2, GLUD1, GLUL, and ALDH5A1, as well as SNARE complex proteins (STX1A, STX1B, STXBP1, and NAPB) and glutamate transporter SLC1A2 were highly enriched with PBS-soluble AD tau aggregates, showing ~27-fold greater SLC1A2 interaction with PBS-soluble AD tau than DLB or PSP tau (Fig. 2C). These interactions imply tau aggregates in glutamate and GABA metabolism and neurotransmitter regulation. Notably, these associations were specific to PBS-soluble AD tau aggregates, with no comparable interactions detected from sarkosyl-soluble AD, PSP, or DLB tau aggregates. Additionally, PBS-soluble AD tau showed enhanced interaction with cytoskeletal proteins (GFAP, NEFH, NEFM, MAP1A, and MAP1B), while several tubulins (TUBA1B, TUBB2A, TUBB3, and TUBB4B) were depleted (Fig. 2D). Furthermore, PBS-soluble AD tau was enriched for proteins related to ROS detoxification (TXN, PRDX1, SOD1, and SOD2), astrocyte differentiation, and apoptosis (Fig. 2E). Additionally, these enriched proteins were also validated in a separate independent cohort (*n* = 5, Table 1B). To biochemically validate the selected interactors from these disease-specific clusters, immunoblotting of IP-T18 with their respective inputs was performed, representative blots are shown in Figs. 2F,G and EV2D–J. Immunoreactivity is observed with FABP5, FH, and SLC1A2 antibodies in IP-T18 materials from PBS- and sarkosyl-soluble fractions, further confirming the presence of these selected proteins in the respective fractions.

Validation by proximity ligation assay (PLA) in AD brain tissues co-stained with MAP2, confirmed significantly more PLA puncta for FABP5 (metabolism), FH (TCA cycle), and SLC1A2 (glutamate transporter) with Tau 13 (total tau) in AD vs PSP vs DLB (Figs. 2H and EV2B,C). Quantification of PLA puncta per region of interest (ROI) demonstrated a statistically significant elevation of all three interactions in AD compared to both PSP and DLB (Fig. 2I; *p* < 0.05 to *p* < 0.0001, Fig. EV2B,C), with no significant differences observed between PSP and DLB for most pairwise comparisons. Taken together, these orthogonal biochemical and histological data demonstrate the direct interactions of FABP5, FH, and SLC1A2 with misfolded tau in a disease-selective manner.

**Figure 3:**
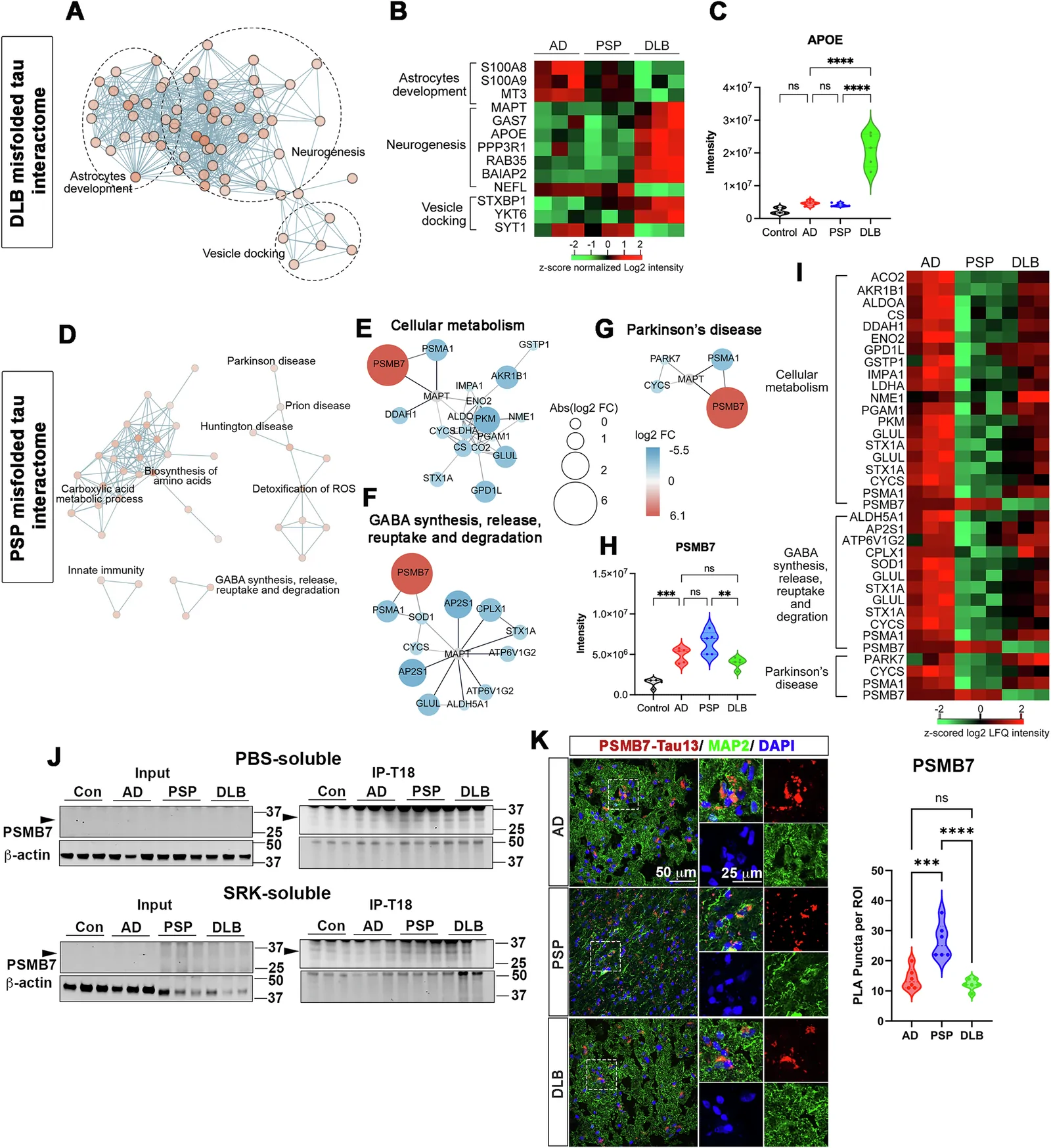
Characterization of the PBS-soluble DLB and PSP tau interactomes. (**A**) Functional landscape of the DLB tau interactome. The network map illustrates the functional enrichment of 20 significant proteins either enriched with, or depleted from, misfolded tau immunoprecipitated from DLB cases. (**B**) Heatmap displaying the relative abundances of significant DLB tau interactors involved in astrocyte development, neurogenesis, and vesicle docking. Data were presented as z-score–normalized log2 LFQ intensities across AD, PSP, and DLB fractions (ANOVA with permutation-based FDR <5%). (**C**) Quantification of APOE targeted PRM-MS intensity across a new set of Control, AD, PSP, and DLB groups. Data were presented as violin plots showing the full data distribution, with individual points representing discrete cases across the Con, AD, PSP, and DLB groups (*n* = 5 independent cases per group). *p < 0.05, **p < 0.01, ***p < 0.001, ****p < 0.0001, ns not significant, determined by one-way ANOVA followed by Tukey’s post hoc test. The adjusted *p* values are as follows: AD vs DLB (*p* < 0.0001), PSP vs DLB (*p* < 0.0001). (**D**) Functional enrichment network of the 57 significant proteins either enriched with, or depleted from, PSP tau. (**E**–**G**) Sub-networks detailing significant PSP tau interactors implicated in (**E**) cellular metabolism, (**F**) GABA synthesis, release, reuptake, and degradation, and (**G**) Parkinson’s disease. Node size correlates with the absolute magnitude of log2 fold change. Node color indicates the log2 FC relative to complement controls (red: enriched in PSP; blue: depleted in PSP), and node size reflects the absolute magnitude. (**H**) Quantification of PSMB7 targeted PRM-MS intensity across a new set of Control, AD, PSP, and DLB groups (*n* = 5). Data were presented as violin plots showing the full data distribution, with individual points representing discrete cases across the Con, AD, PSP, and DLB groups (*n* = 5 independent cases per group). *p < 0.05, **p < 0.01, ***p < 0.001, ****p < 0.0001, ns not significant, determined by one-way ANOVA followed by Tukey’s post hoc test. The adjusted *p* values are as follows: Con vs AD (*p* = 0.0001), PSP vs DLB (*p* = 0.0025). (**I**) Heatmap of significant PSP tau interactors highlighted in the sub-networks (**E**–**G**), shown as z-score–normalized log2 LFQ intensities across AD, PSP, and DLB (ANOVA with permutation-based FDR <5%). (**J**) Biochemical validation of the tau-PSMB7 interaction. Representative WBs of inputs and misfolded-tau (T18) immunoprecipitates (IP-T18) from both PBS-soluble and sarkosyl-soluble (SRK-soluble) brain extracts of Con, AD, PSP, and DLB cases. Blots were probed for PSMB7, with β-actin serving as a loading control. All the proteins of interest probed for here have been detected on the same, re-probed membrane for marker proteins in Figs. 2 and 6. (**K**) In situ validation of the PSMB7-tau interaction. **Left:** Representative confocal images of PLA showing PSMB7-Tau13 interactions (red puncta) in AD, PSP, and DLB brain tissues. Neurons are counterstained with MAP2 (green) and nuclei with DAPI (blue). Scale bars: 50 μm (main panels) and 25 μm (magnified right panels). Right: Quantification of PLA puncta per region of interest (ROI) across the disease groups. (For **C**,** H**,** K**) Data were presented as violin plots showing the data distribution, with individual points representing discrete cases (*n* = 3 independent cases per group). *p < 0.05, **p < 0.01, ***p < 0.001, ****p < 0.0001, ns not significant, determined by one-way ANOVA followed by Tukey’s post hoc test. The adjusted *p* values are as follows: AD vs PSP (*p* = 0.0002), PSP vs DLB (*p* < 0.0001). Source data are available online for this figure.

In marked contrast to the metabolically focused AD tau interactome, functional enrichment analysis of DLB tau-associated proteins (20 significant proteins) identified three distinct annotation clusters: astrocyte development, neurogenesis, and vesicle docking (Fig. 3A). Notably, heatmap analysis showed that neuroinflammatory mediators, S100A8, S100A9, and metallothionein-3 (MT3) were significantly depleted from DLB tau compared to AD and PSP tau, while neurogenesis-related proteins (1N4R tau isoform, GAS7, APOE, BAIAP2) were enriched, and NEFL was depleted (Fig. 3B). Quantification of APOE protein intensity, by a measure of targeted parallel reaction monitoring-mass spectrometry (PRM-MS) across an independent cohort of control, AD, PSP, and DLB cases (*n* = 5 per group) validated this disease-selective enrichment, revealing significantly elevated APOE abundance in DLB relative to all other groups examined (Fig. 3C; Table 1B; **p < 0.01 to ****p < 0.0001).

PSP tau exhibited the most distinctive interactome profile, with only 5 of 57 significant proteins showing enrichment (TRIM21, PSMB7, TBC1D10B, IGHG1, and NUDT21), while the majority (52/57) were depleted compared to AD and DLB tau. These proteins were functionally associated with cellular metabolism, stress response/ROS detoxification, GABA neurotransmission, and innate immunity (Fig. 3D), as demonstrated in corresponding network and heatmap analysis. In direct opposition to AD tau findings, metabolic proteins were predominantly depleted from PSP tau (Fig. 3E,I), as were proteins involved in GABA synthesis, release, and degradation (Fig. 3F,I). Several Parkinson’s disease-associated proteins, including PARK7, were also depleted from PSP tau (Fig. 3G,I). Notably, PSMB7 (20 s proteosome core) emerged as one of the few proteins selectively enriched with PSP tau; quantification using targeted PRM-MS of PSMB7 protein intensity in an independent cohort (*n* = 5 per group, Table 1B) validated this observation, demonstrating significantly elevated PSMB7 abundance in PSP relative to control, AD, and DLB (Fig. 3H; **p < 0.01, ***p < 0.001 to ****p < 0.0001). Immunoblotting of IP-T18 eluates for PSMB7 demonstrated co-immunoprecipitation of PSMB7 with misfolded tau in PSP brain extracts, which was greater than the co-immunoprecipitation observed in AD, DLB, and control cases across both solubility fractions (Fig. 3J). In situ validation via PLA in postmortem brain sections further confirmed the PSMB7-tau interaction. PLA signals for PSMB7-Tau13 pairs detected within MAP2-positive neuronal somata were significantly more abundant in PSP brain sections relative to AD and DLB (Fig. 3K). Quantification of PLA puncta per ROI demonstrated statistically significant elevations of the PSMB7-Tau13 interaction in PSP compared to both AD and DLB (***p < 0.001 to ****p < 0.0001), with no significant difference observed between AD and DLB (Fig. 3K). Taken together, these biochemical and histological data identify PSMB7 as a preferential interactor of PSP tau, implicating aberrant proteasomal engagement as a distinguishing molecular feature of PSP tau pathology.

### Sarkosyl-soluble tau fractions exhibit reduced interactome complexity with disease-specific signatures

**Figure 4:**
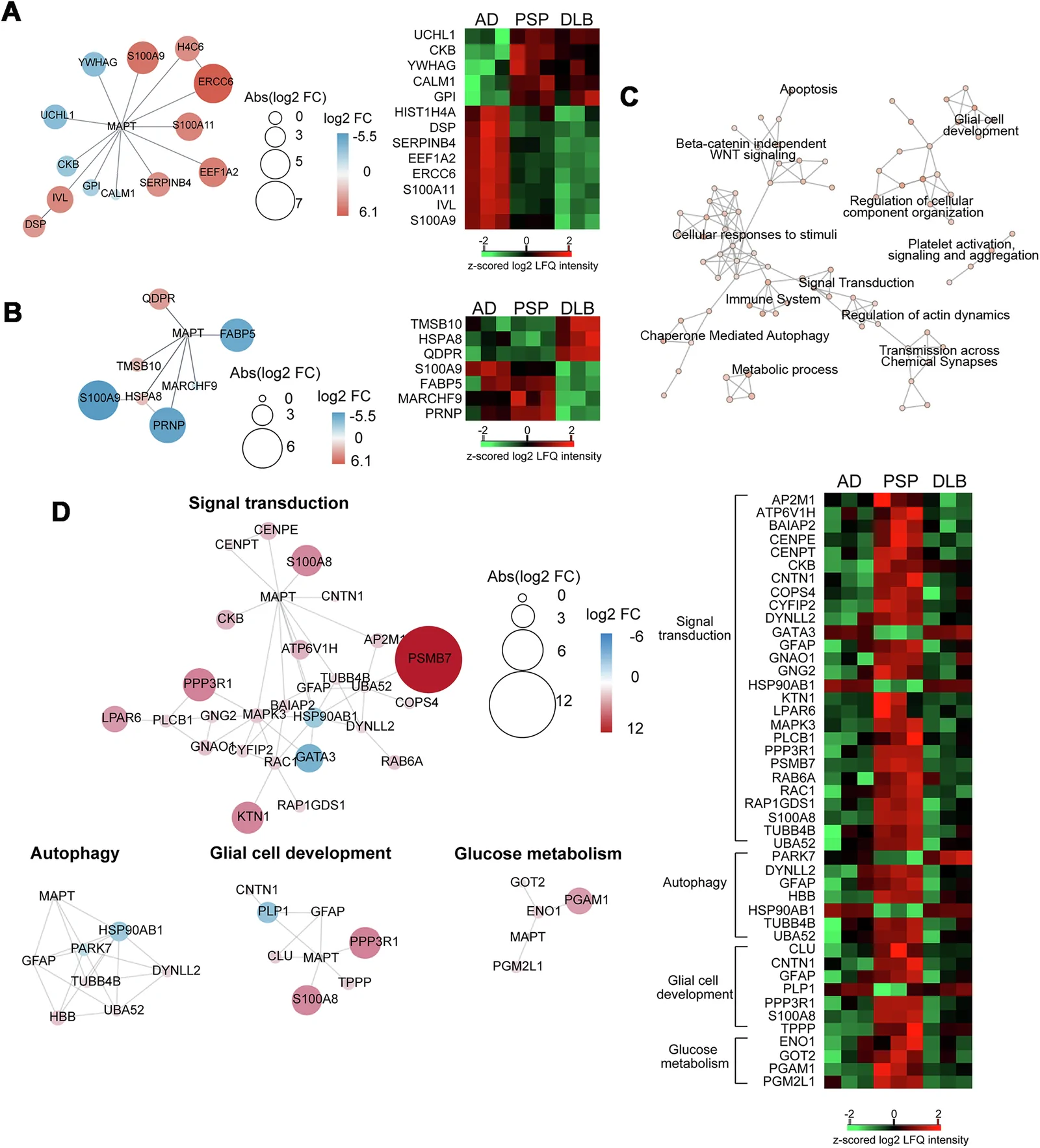
Characterization of disease-specific sarkosyl-soluble tau interactomes. (**A**) Left: Protein interaction network of significant sarkosyl-soluble AD tau interactors in the fraction of AD brains. Node color (red: enriched; blue: depleted) and size correspond to log2 FC. Right: Heatmap detailing the relative abundance of these significant AD tau interactors, presented as z-score–normalized log2 LFQ intensities across AD, PSP, and DLB cases (ANOVA with permutation-based FDR <5%). (**B**) Left: Protein interaction network of significant sarkosyl-soluble DLB tau interactors. Node color (red: enriched; blue: depleted) and size correspond to log2 FC. Right: Heatmap showing the relative abundance of these significant DLB tau interactors across the three disease cohorts. (**C**) Functional landscape of the PSP tau interactome. The network map illustrates the broad functional enrichment of the 103 significant proteins either enriched with, or depleted from, sarkosyl-soluble PSP tau. (**D**) Left: Sub-network analysis detailing specific functional clusters of significant PSP tau interactors, specifically those involved in signal transduction, autophagy, glial cell development, and glucose metabolism. Node color (red: enriched; blue: depleted) and size correspond to log2 FC. Right: Heatmap displaying the z-score–normalized log2 LFQ intensities of the proteins highlighted in these specific functional sub-networks across the AD, PSP, and DLB fractions.

While PBS-soluble tau fractions demonstrated extensive protein interactions, sub-network analysis of the sarkosyl-soluble fractions revealed markedly fewer significant protein associations across all three diseases. MS analysis identified only a limited number of proteins significantly enriched or depleted from sarkosyl-soluble AD or DLB tau, with corresponding protein networks and respective heatmaps are shown in Fig. 4A,B. Although functional enrichment analysis of these proteins did not yield significantly enriched terms, functionally important proteins were identified, including ubiquitin carboxyl-terminal hydrolase isozyme L1 (UCHL1), which was depleted from sarkosyl-soluble AD tau, and E3 ubiquitin-protein ligase MARCH9, which was depleted from sarkosyl-soluble DLB tau (Fig. 4A,B).

In contrast to AD and DLB, sarkosyl-soluble PSP tau demonstrated a more robust interactome, with MS analysis identifying 103 significant proteins that yielded multiple significantly enriched annotation terms (Fig. 4C). Sub-network analysis further delineated discrete functional clusters within this interactome, highlighting PSP tau interactors associated with signal transduction, autophagy, glial cell development, and glucose metabolism (Fig. 4D, left). Heatmap visualization of these interactors confirmed that their relative abundance profiles were substantially distinct in PSP compared to AD and DLB (Fig. 4D, right), further corroborating the disease-selective nature of these associations. Taken together, these findings indicate that PSP tau retains a functionally diverse interactome within the sarkosyl-soluble fraction, a pattern not observed for AD or DLB, and implicate dysregulation of signal transduction, autophagic clearance, glial homeostasis, and glucose metabolism as molecular features selectively associated with insoluble PSP tau pathology.

### Machine learning classification identifies disease-specific tau interactome signatures

**Figure 5:**
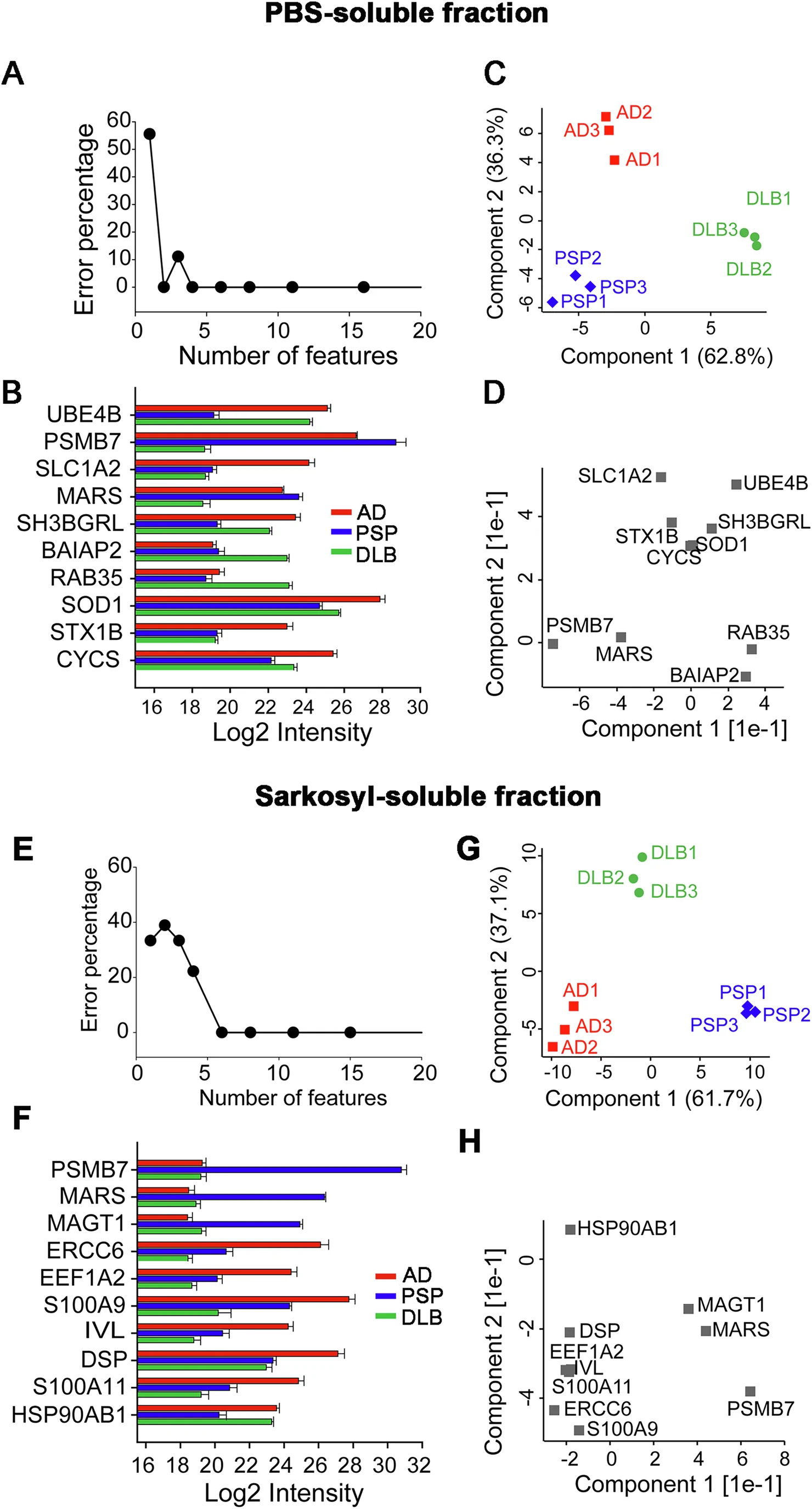
Classification analysis of disease-specific PBS- and sarkosyl-soluble tau interactomes. (**A**) The prediction error percentage corresponding to the number of features used for disease phenotype prediction in PBS-soluble fraction by using the SVM algorithm. (**B**) Abundance (Log2 LFQ intensity) of the ten most informative proteins in PBS-soluble fraction across AD (red), PSP (blue), and DLB (green) tau interactomes (*n* = 3 independent cases per group). Bar graphs represent the mean ± SEM. (**C**) PCA analysis using the abundances of these top ten proteins in PBS-soluble fraction across AD (red), PSP (blue), and DLB (green) tau interactomes. (**D**) The PCA loadings of the top ten proteins in the PBS-soluble fraction. It illustrates how much each protein influences the variance captured by that component. (**E**) The prediction error percentage corresponding to the number of features used for disease phenotype prediction in the sarkosyl-soluble fraction by using the SVM algorithm. (**F**) Abundance (Log2 LFQ intensity) of the ten most informative proteins in sarkosyl-soluble fraction across AD (red), PSP (blue), and DLB (green) tau interactomes (*n* = 3 independent cases per group). Bar graphs represent the mean ± SEM. (**G**) PCA analysis using the abundances of these top ten proteins in sarkosyl-soluble fraction across AD (red), PSP (blue), and DLB (green) tau interactomes. (**H**) The PCA loadings of the top ten proteins in the sarkosyl-soluble fraction. It illustrates how much each protein influences the variance captured by that component.

**Figure EV3:**
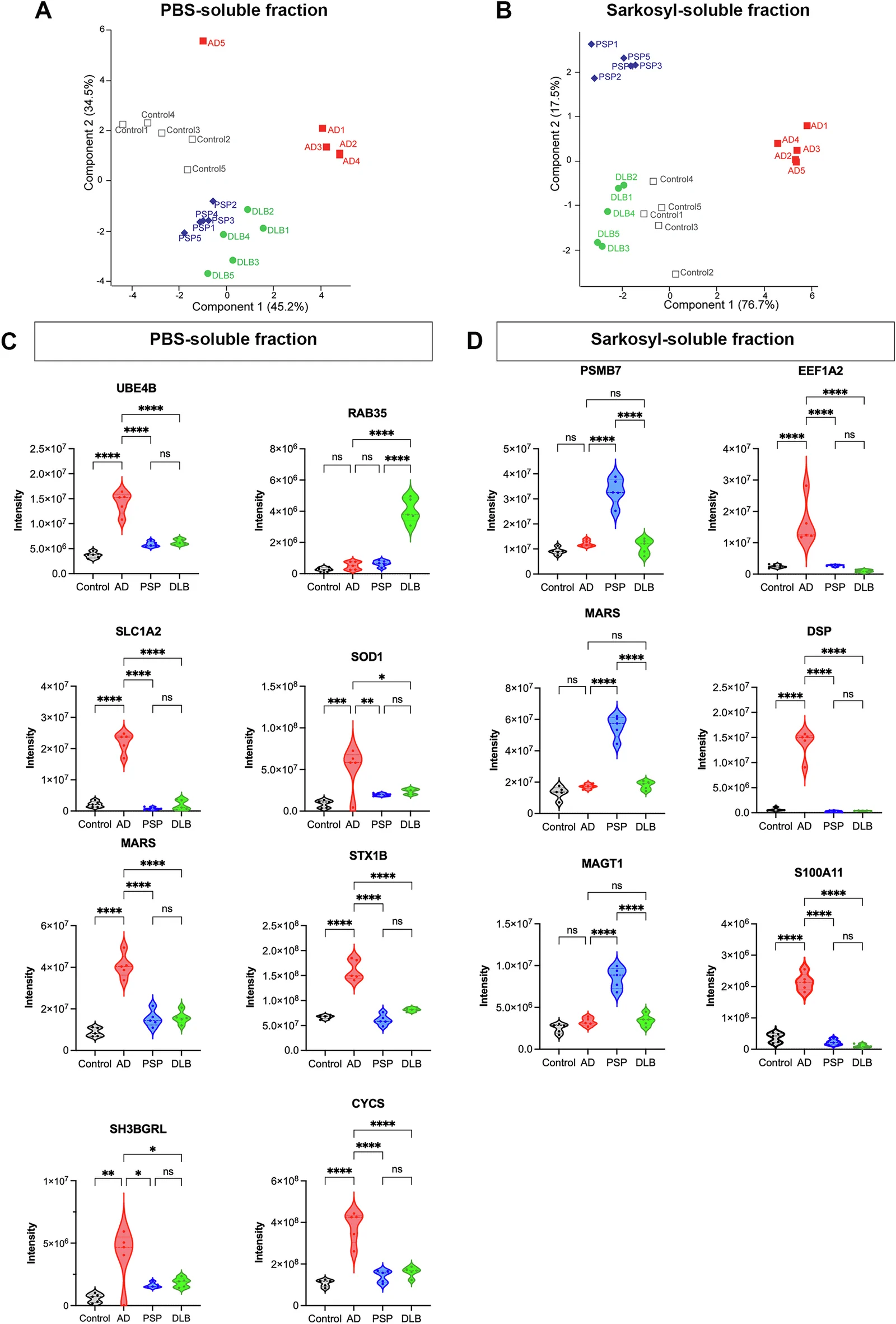
Related to Fig. 5. Validation of targeted tau interactors in an independent cohort via PRM-MS. (**A**,** B**) Principal component analysis (PCAs) illustrating the distinct segregation of Con (gray), AD, (red), PSP, (blue), and DLB, (green) cases based on their targeted PRM-MS protein profiles. The distinct clustering demonstrates disease-specific interactomic signatures in both (**A**) PBS-soluble and (**B**) sarkosyl-soluble fractions of the independent validation cohort (*n* = 5 independent cases per group). (**C**,** D**) Targeted quantification of selected significant interactors using PRM-MS in the (**C**) PBS-soluble fraction (UBE4B, RAB35, SLC1A2, SOD1, MARS, STX1B, SH3BGRL, and CYCS) and (**D**) sarkosyl-soluble fraction (PSMB7, EEF1A2, MARS, DSP, MAGT1, and S100A11). Data were presented as violin plots showing the full data distribution, with individual points representing discrete cases across the Con, AD, PSP, and DLB groups (*n* = 5 independent cases per group). *p < 0.05, **p < 0.01, ***p < 0.001, ****p < 0.0001, ns not significant, determined by one-way ANOVA followed by Tukey’s post hoc test. The adjusted *p* values are as follows: (**C**) UBE4B; Con vs AD (*p* < 0.0001), AD vs PSP (*p* < 0.0001), AD vs DLB (*p* < 0.0001), SLC1A2; Con vs AD (*p* < 0.0001), AD vs PSP (*p* < 0.0001), AD vs DLB (*p* < 0.0001), MARS; Con vs AD (*p* < 0.0001), AD vs PSP (*p* < 0.0001), AD vs DLB (*p* < 0.0001), SH3BGRL; Con vs AD (*p* = 0.0015), AD vs PSP (*p* = 0.0261), AD vs DLB (*p* = 0.0494), RAB35; AD vs DLB (*p* < 0.0001), PSP vs DLB (*p* < 0.0001), SOD1; Con vs AD (*p* = 0.0006), AD vs PSP (*p* = 0.0089), AD vs DLB (*p* = 0.0235), STX1B; Con vs AD (*p* < 0.0001), AD vs PSP (*p* < 0.0001), AD vs DLB (*p* < 0.0001), CYCS; Con vs AD (*p* < 0.0001), AD vs PSP (*p* < 0.0001), AD vs DLB (*p* < 0.0001), (**D**) PSMB7; AD vs PSP (*p* < 0.0001), PSP vs DLB (*p* < 0.0001), MARS; AD vs PSP (*p* < 0.0001), PSP vs DLB (*p* < 0.0001), MAGT1; AD vs PSP (*p* < 0.0001), PSP vs DLB (*p* < 0.0001), EEF1A2; Con vs AD (*p* < 0.0001), AD vs PSP (*p* < 0.0001), AD vs DLB (*p* < 0.0001), DSP: Con vs AD (*p* < 0.0001), AD vs PSP (*p* < 0.0001), AD vs DLB (*p* < 0.0001), S100A11; Con vs AD (*p* < 0.0001), AD vs PSP (*p* < 0.0001), AD vs DLB (*p* < 0.0001). Source data are available online for this figure.

Machine learning analysis was employed as an exploratory discovery to identify the most discriminative features of tau interactome across disease phenotypes. In the PBS-soluble fraction, ANOVA identified 264 significantly different proteins (permutation-based FDR <0.01), and Support Vector Machine classification with fourfold cross-validation achieved 0% prediction error using only the top four features (Fig. 5A). Principal component analysis (PCA) of the top ten proteins (Table 2; Figs. 5B and EV3; Dataset EV6) demonstrated distinct segregation of AD, PSP, and DLB cases (Fig. 5C), with PCA loadings revealing disease-specific contributions (Fig. 5D). SLC1A2 and STX1B interactions were most distinctive for AD (38× and 13× more abundant than DLB/PSP, respectively), BAIAP2 and RAB35 characterized DLB, while PSMB7 and MARS distinguished PSP.

**Table 2:** Top ten features of disease-specific misfolded tau interactors in PBS-soluble and Sarkosyl-soluble fractions.

Ranks	PBS-soluble tau interactome			Sarkosyl-soluble tau interactome		
	Gene names	Protein IDs	Protein names	Gene names	Protein IDs	Protein names
1	UBE4B	O95155	Ubiquitin conjugation factor E4 B	PSMB7	Q99436	Proteasome subunit beta type-7
2	PSMB7	Q99436	Proteasome subunit beta type-7	MARS	P56192	Methionine--tRNA ligase, cytoplasmic
3	SLC1A2	P43004	Excitatory amino acid transporter 2	MAGT1	Q9H0U3	Magnesium transporter protein 1
4	MARS	P56192	Methionine--tRNA ligase, cytoplasmic	ERCC6	Q03468	DNA excision repair protein ERCC6
5	SH3BGRL	O75368	SH3 domain-binding glutamic acid-rich-like protein	EEF1A2	Q05639	Elongation factor 1-alpha 2
6	BAIAP2	Q9UQB8	Brain-specific angiogenesis inhibitor 1-associated protein 2	S100A9	P06702	Protein S100A9
7	RAB35	Q15286	Ras-related protein Rab-35	IVL	P07476	Involucrin
8	SOD1	P00441	Superoxide dismutase (Cu-Zn)	DSP	P15924	Desmoplakin
9	STX1B	P61266	Syntaxin-1B	S100A11	P31949	Protein S100A11
10	CYCS	P99999	Cytochrome C	HSP90AB1	P08238	Heat shock protein HSP 90-beta

Similarly, in the sarkosyl-soluble fraction, 166 significant proteins were identified, with the top six features achieving 0% classification error (Fig. 5E). PCA of the top ten proteins (Table 2; Figs. 5F and EV3; Dataset EV6) again showed clear disease separation (Fig. 5G). Notably, PSMB7 and MARS remained highly distinctive for PSP across both fractions, with PSMB7 showing >3000-fold enrichment in sarkosyl-soluble PSP tau compared to other diseases (Fig. 5H). AD was characterized by interactions with ERCC6, EEF1A2, and S100A11, while HSP90AB1 was strongly associated with DLB. These findings demonstrate that tau interactomes contain robust, disease-specific molecular signatures that enable exploratory feature discovery classification.

To assess the generalizability of these disease-specific interactions beyond the discovery cohort, we examined an independent cohort of human brain tissue (*n* = 5 per disease group, Table 1B) by targeted PRM-MS. We monitored the interactors that most strongly discriminated AD, PSP, and DLB in the classification analysis (Table 2). The independent cohort corroborated the disease-specific associations observed by targeted PRM-MS, with the AD-, PSP-, and DLB-enriched interactors reproducing their respective disease specificity (Fig. EV3; Dataset EV6). Together, these results indicate that the disease-specific misfolded tau interactors identified here are reproducible in an independent cohort.

### Ubiquitination enzymes modify disease-specific tau conformations

**Figure 6:**
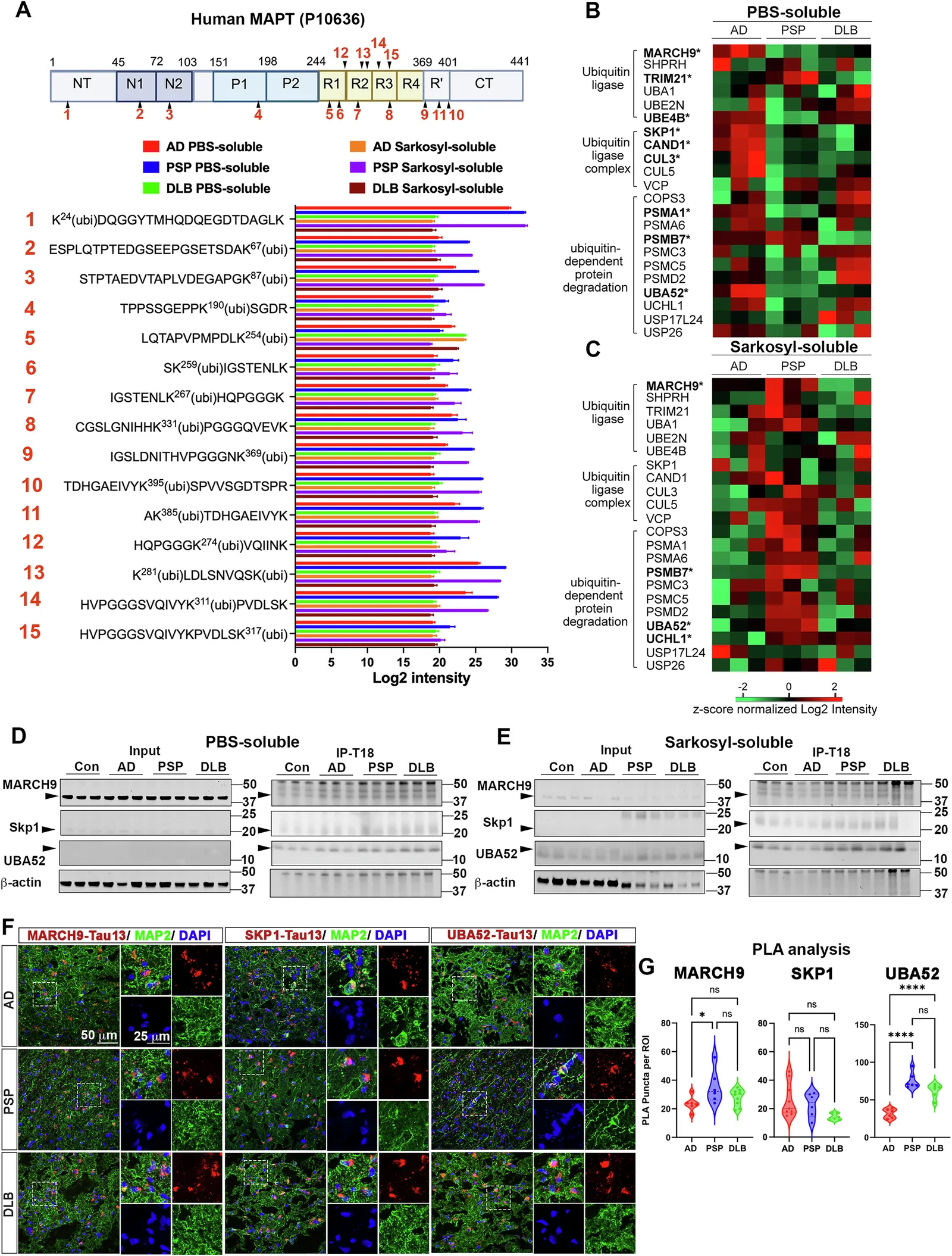
Ubiquitination profiling of tau and validation of ubiquitination-related interactors. (**A**) Top: Domain map of human tau (MAPT, UniProt P10636) highlighting the locations of 15 identified ubiquitinated lysine residues (red arrows, 1–15). Bottom: Quantitative profiles of the 15 ubiquitinated tau peptides, expressed as log2 intensity, demonstrating significant variance across AD, PSP, and DLB in both PBS- and sarkosyl-soluble fractions (*n* = 3 independent cases per group). Bar graph represents mean ± SEM (ANOVA with permutation-based FDR <0.01). (**B**,** C**) Heatmaps detailing the relative abundance of ubiquitination-related tau interactors in (**B**) PBS-soluble and (**C**) sarkosyl-soluble fractions. Proteins are categorized into ubiquitin ligases, ubiquitin ligase complexes, and ubiquitin-dependent protein-degradation pathways. Proteins in bold and marked with an asterisk (*) indicate statistical significance (ANOVA with permutation-based FDR <0.01). Data are presented as z-score–normalized log2 intensities across AD, PSP, and DLB cases. (**D**,** E**) Biochemical validation of tau interaction with ubiquitination machinery. Representative Western blots of inputs and misfolded tau (T18) immunoprecipitates (IP-T18) from (**D**) PBS-soluble and (**E**) sarkosyl-soluble extracts of Con, AD, PSP, and DLB brains. Blots were probed for MARCH9 (ubiquitin E3 ligase), Skp1 (SCF complex), and UBA52 (ubiquitin-ribosomal fusion protein). β-actin serves as the loading control. All the proteins of interest probed for here have been detected on the same, re-probed membrane for marker proteins in Figs. 2 and 3. (**F**) In situ validation of protein interactions. Representative confocal micrographs of PLA demonstrating spatial colocalization (red puncta) of Tau13 with MARCH9, SKP1, and UBA52 in AD, PSP, and DLB postmortem brain tissues. Neurons are counterstained with MAP2 (green) and nuclei with DAPI (blue). Scale bars: 50 μm (main panels) and 25 μm (magnified right panels). (**G**) Quantification of PLA puncta per region of interest (ROI) for the interactions shown in (**F**). Data were presented as violin plots illustrating the full data distribution (*n* = 3 independent cases per group). *p < 0.05, ****p < 0.0001, ns not significant, determined by one-way ANOVA followed by Tukey’s post hoc test. The adjusted *p* values are as follows: Tau13 with MARCH9; AD vs PSP (*p* = 0.0412), and Tau13 with UBA52; AD vs PSP (*p* < 0.0001), AD vs DLB (*p* < 0.0001). Source data are available online for this figure.

Proteomic profiling identified 20 ubiquitinated tau peptides across AD, PSP, and DLB, with 15 peptides exhibiting significant disease-specific differences after normalization to total tau (Fig. 6A and EV5). In PBS-soluble fractions, ubiquitination at Lys-24, Lys-87, Lys-267, Lys-300, Lys-311, Lys-338, and Lys-385 (2N4R/2N3R isoforms) was elevated in AD tau compared to DLB but remained lower in PSP tau. PSP tau showed globally elevated ubiquitination in both PBS- and sarkosyl-soluble fractions, while DLB tau displayed the lowest levels except for Lys-254 (2N4R), which was specifically increased. In sarkosyl-soluble fractions, most AD tau ubiquitination resembled DLB, except for Lys-254, which was higher in AD than PSP (Fig. 6A).

Interactome studies revealed that E1 (UBA1) and E2 (UBE2N) ubiquitin enzymes did not show disease-specific interactions, whereas E3 ligases demonstrated differential enrichment across disease groups (Fig. 6B,C). In the PBS-soluble fraction, AD tau specifically interacted with E3 ligases MARCH9 and UBE4B, while PSP tau was enriched for TRIM21. DLB tau lacked significant E3 interaction, consistent with its low ubiquitination profile (Fig. 6B). In the sarkosyl-soluble fraction, PSP tau uniquely enriched for MARCH9; AD and DLB tau did not display significant E3 associations (Fig. 6C). PBS-soluble AD tau also associated with cullin-RING ligase complex components CUL3, SKP1, and CAND1, implicating the B-Cell Receptor and Skp1-Cullin 1-F-box complexes in AD-specific ubiquitination. Furthermore, UBA52, a ubiquitin-ribosomal fusion protein, was identified as a differentially enriched misfolded-tau interactor with disease-selective abundance across AD, PSP, and DLB (Fig. 6B, C). Several candidate substrates of these ligase complexes, including the NF-κB inhibitors (NFKBIB and NFKBIE) and additional proteins implicated in signaling and cell cycle regulation, were identified as potential ubiquitination targets within the misfolded-tau interactome.

Deubiquitinating enzymes UCHL1, USP17L24, and USP26 did not show disease-specific interactions with tau aggregates in the PBS-soluble fraction; however, in the sarkosyl-soluble fraction, UCHL1 binding to AD tau was significantly decreased compared to DLB and PSP (Fig. 6C), which may influence the elevated ubiquitination at Lys-254 in insoluble AD tau.

The tau interactome also showed that only PSP tau aggregates, in both PBS- and sarkosyl-soluble fractions, interacted significantly with PSMB7 (20S proteasome core), while AD and DLB tau did not exhibit substantial proteasome association (Fig. 6B,C). This unique association suggests differential proteasomal targeting among tauopathies. Additionally, these enriched proteins were also validated in a separate independent cohort (*n* = 5, Table 1B).

**Figure EV4:**
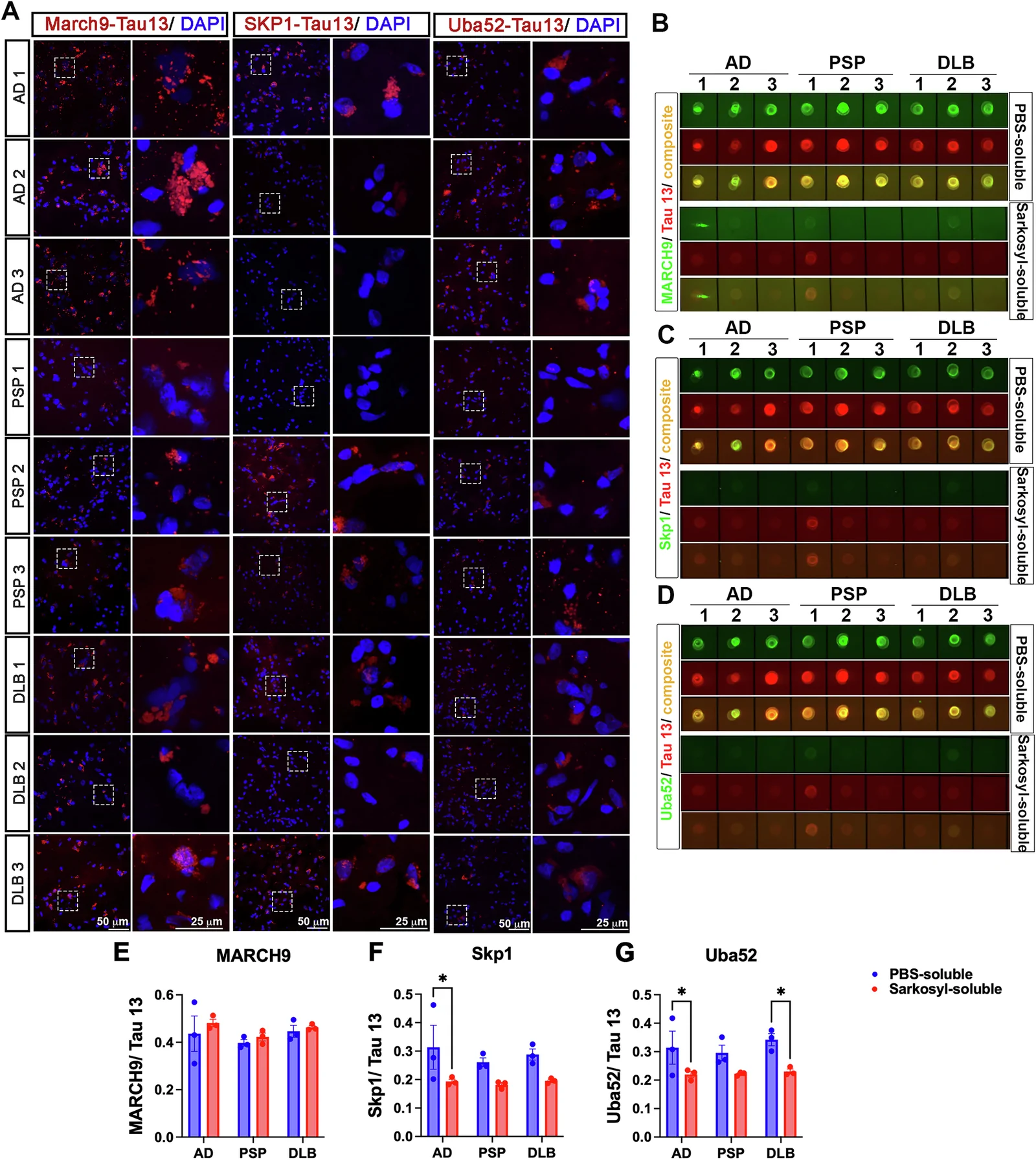
Biochemical validation of ubiquitination-related tau interactors. Related to Fig. 6. (**A**) Representative PLA images showing colocalization of Tau13 with ubiquitin ligase MARCH9, ubiquitin ligase complex Skp1, and ubiquitin-dependent protein-degradation UBA52 in postmortem brain tissue from AD (AD1–3), PSP (PSP1–3), and DLB (DLB1–3) cases. Boxed regions are magnified at right. Scale bars: 50 μm (left panels), 25 μm (zoom, right panels). (**B**–**D**) Dot blot analysis of tau immunoprecipitated from PBS- and sarkosyl-soluble brain fractions using Tau13 in combination with antibodies against (**B**) MARCH9, (**C**) Skp1, or (**D**) UBA52. Tau was immunoprecipitated using the T18 antibody from AD, PSP, and DLB PBS-and sarkosyl-soluble fractions (*n* = 3 per disease group). (**E**–**G**) Quantification of dot-blot signals expressed as protein interactor/Tau13 intensity ratios for (**E**) MARCH9, (**F**) SKP1, and (**G**) UBA52 across AD, PSP, and DLB in PBS (blue) and Sarkosyl (red) fractions. Data were mean ± SEM (*n* = 3 per disease); statistics by two-way ANOVA with Tukey’s post hoc test (*p < 0.05, **p < 0.01). The adjusted *p* values are as follows: (**F**) SKP1; PBS-soluble vs sarkosyl-soluble AD tau (*p* = 0.0265), (**G**) Uba52; PBS-soluble vs sarkosyl-soluble AD tau (*p* = 0.0368), PBS-soluble vs sarkosyl-soluble DLB tau (*p* = 0.0165). Source data are available online for this figure.

Representative immunoblots of IP-T18 eluates for MARCH9, SKP1, and UBA52 demonstrated selective co-immunoprecipitation of all three proteins with misfolded tau in a disease-specific pattern in the PBS-soluble fraction, consistent with the enrichment profiles observed in the interactome heatmap (Fig. 6D). In the sarkosyl-soluble fraction, co-IP of MARCH9, SKP1, and UBA52 with IP-T18 was substantially altered relative to the PBS-soluble profiles, further corroborating the fraction-dependent nature of these interactions (Fig. 6E). Confirmation by PLA with Tau13 and co-stained with MAP2, (Figs. 6F,G and EV4) corroborated the interactome findings regarding E3 ligases, SCF components, and proteasome subunits.

Collectively, these data demonstrate that tau aggregates from AD, PSP, and DLB display distinct profiles of ubiquitination, shaped primarily by differential E3 ligase interactions and supported by unique associations with ligase complexes and the proteasome system.

### Distinct acetylation and phosphorylation profiles are associated with disease-specific tau aggregates

**Figure 7:**
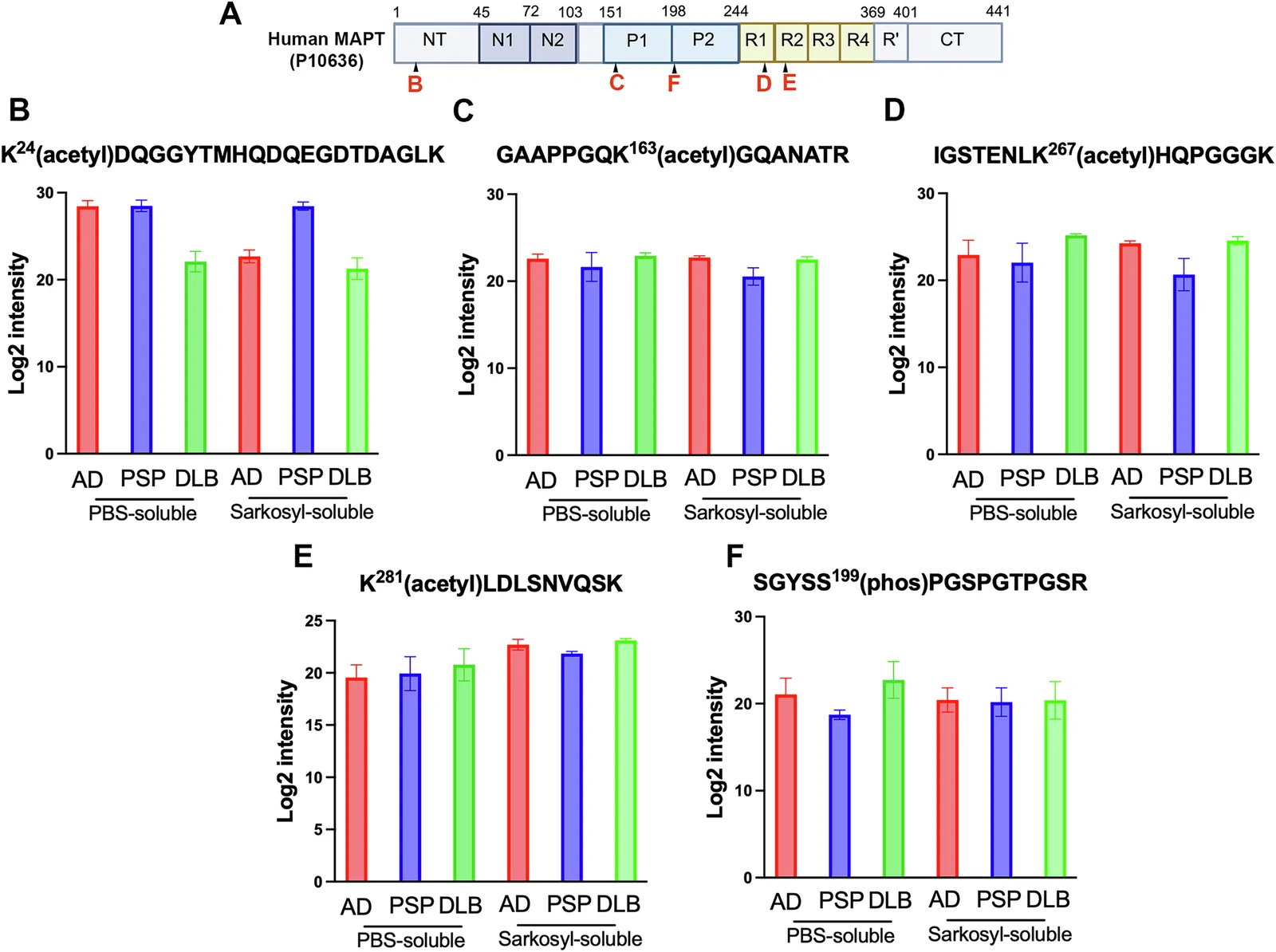
Acetylation and phosphorylation of disease-specific tau aggregates. (**A**) MAPT domain schematic of the acetylation (**B**–**E**) and phosphorylation sites (**F**) with their respective quantified peptides that exhibited significant variance in a disease-specific manner (*n* = 3 independent cases per group). Bar graphs represent the mean ± SEM (ANOVA with permutation-based FDR <0.01). Source data are available online for this figure.

**Figure EV5:**
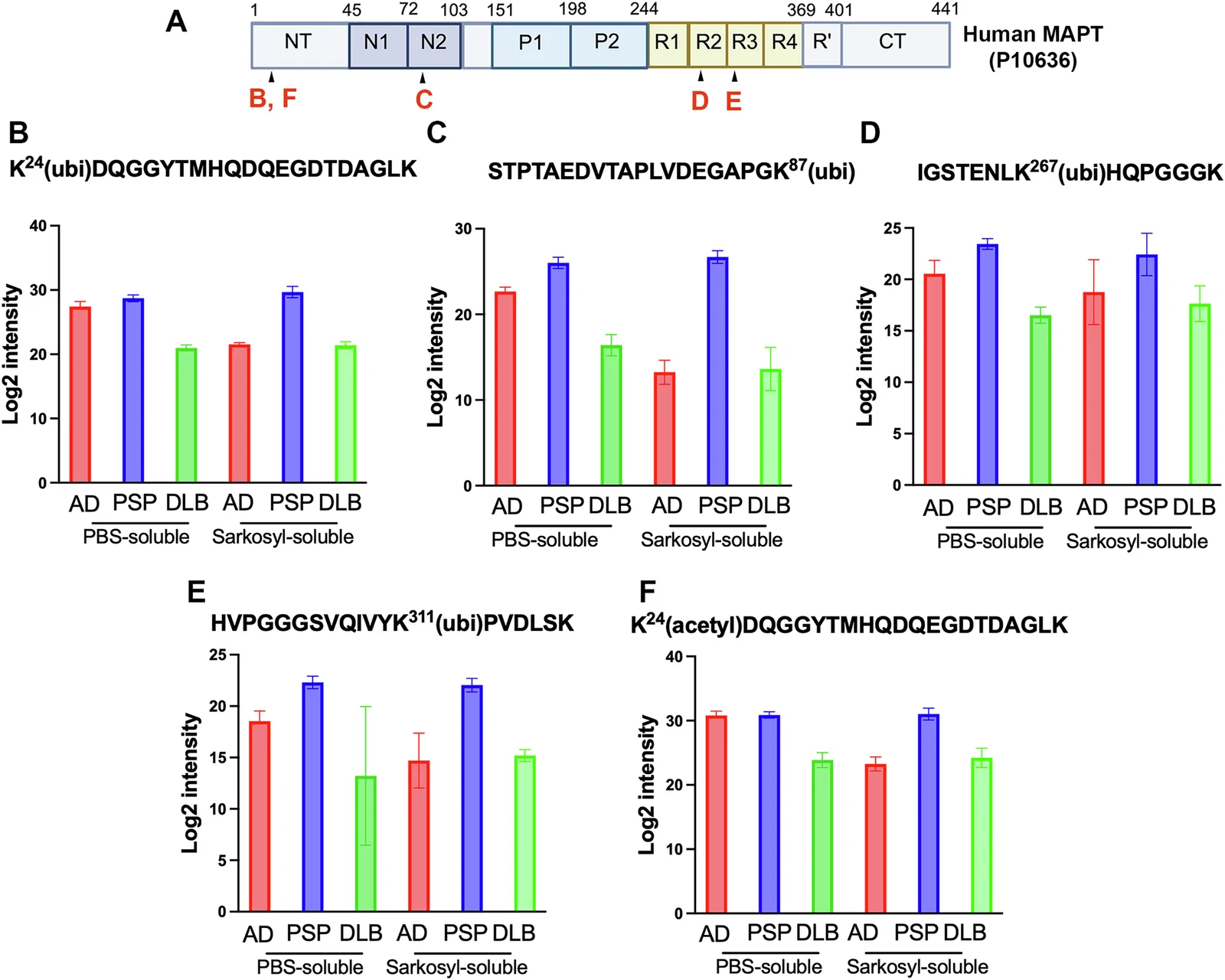
Related to Figs. 6 and 7. PRM-MS validation of tau ubiquitination and acetylation in the tau interactome. (**A**) Domain map of human MAPT (UniProt P10636) with red letters marking the locations of the quantified peptides. (**B**–**E**) Targeted PRM-MS quantification of four ubiquitinated tau peptides in T18 immunoprecipitates from PBS-soluble and Sarkosyl-soluble fractions of AD, PSP, and DLB brains (*n* = 3 per disease). Sequences shown on each panel with modified lysines marked “[ubi]”. Bars are log₂ PRM intensities (mean ± SEM). (**F**) Targeted PRM-MS quantification of an acetylated tau peptide (K^24^[ac]DQGGYTMHQDQEGDTDAGLK) across the same samples and fractions. Bars are log₂ PRM intensities (mean ± SEM).

Tau lysine residues are subject to acetylation, which can compete with ubiquitination at the same sites and influence protein stability. Proteomic analysis identified seven acetylation sites on tau, with Lys-24, Lys-163, Lys-267, and Lys-281 showing significant disease-specific differences (Fig. 7A–E). Acetylation of Lys-267, Lys-281, and Lys-163 was notably lower in PSP tau aggregates compared to AD and DLB, whereas acetylation of Lys-24 was significantly elevated in PBS-soluble AD and PSP tau, as well as in sarkosyl-soluble PSP tau. These profiles were validated by PRM-MS (Fig. EV5). No specific acetyltransferase or deacetylase was found to be associated with tau aggregates in the interactome analysis, leaving the enzymatic basis of these modifications unresolved.

In parallel, six phosphorylation sites were detected, with phosphorylation at Ser-199 being markedly higher in AD and DLB tau than in PSP tau (Fig. 7A,F), highlighting a disease-specific phosphorylation signature relevant to neurofibrillary degeneration (Maurage et al, 2001, 2003).

Together, these findings demonstrate that disease-specific tau polymorphs exhibit distinct patterns of ubiquitination, acetylation, and phosphorylation. Among these modifications, ubiquitination displays the most pronounced disease-specific variation.

## Discussion

**Figure 8:**
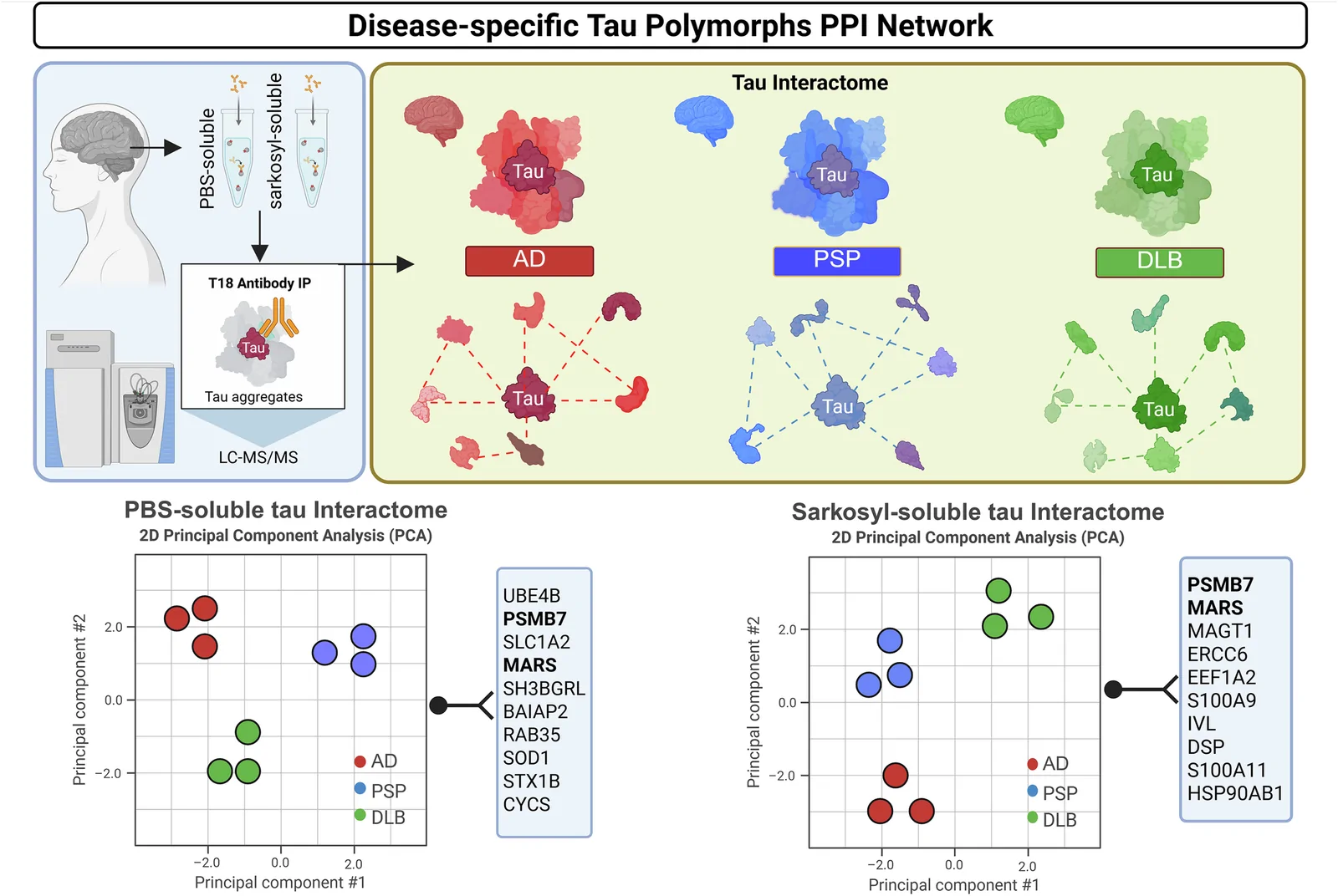
Schematic overview of the study.

This study provides the first comparative, proteomic-based characterization of misfolded tau interactomes isolated from PBS- and sarkosyl-soluble fractions of AD, PSP, and DLB postmortem brains. By coupling conformation-specific capture of misfolded tau with label-free LC-MS, we uncover three principal and interrelated findings (Fig. 8): (1) Tau aggregates form disease-specific interaction networks with distinct molecular and cellular functions; (2) PBS-soluble AD tau harbor richer and more functionally coherent interactomes than sarkosyl-soluble fractions, while sarkosyl-soluble PSP tau forms more protein complexes than PBS-soluble PSP tau. Neither PBS-soluble nor sarkosyl-soluble DLB tau has many interactors; (3) The pattern of PTMs, especially ubiquitination, but also acetylation and phosphorylation, aligns with disease-specific E3 ligase and proteasome associations. Together, these findings underscore that the conformational heterogeneity of tau polymorphs, along with the formation of different tau-protein complexes, drives divergent cellular pathobiology and explains the heterogeneity of tau pathologies.

The distinct tau interactomes and PTM profiles in the brains of AD, PSP, and DLB support the concept that tau adopts disease-specific conformations that expose different interaction surfaces and therefore recruit different sets of cellular proteins. Our previous as well as high-resolution CryoEM studies have demonstrated disease-specific soluble and paired helical filaments (PHF) in AD vs unique folds in several 4R tauopathies, providing compelling evidence for selective interactor recruitment (Fitzpatrick et al, 2017; Lo Cascio et al, 2025; Shi et al, 2021). Moreover, the apparent differences in the PBS-soluble fraction suggest that early conformers rather than late are the principal determinants of selective recruitment and early cellular dysfunction (Balducci et al, 2025; Colom-Cadena et al, 2023; Kopeikina et al, 2012; Lasagna-Reeves et al, 2011; Lasagna-Reeves et al, 2012a; Takeda et al, 2015; Tanaka et al, 2019).

PBS-soluble AD tau selectively co-immunoprecipitated glycolytic enzymes (HK1, PKM, and PFKM) and TCA/mitochondrial proteins (CS, ACO2, MDH2, FH, ETFA, and CYCS), and strongly enriched glutamate/GABA cycle enzymes and the astrocytic glutamate transporter SLC1A2/EAAT2. This pattern, combined with extensive literature implicating early metabolic and mitochondrial dysfunction in AD, suggests that tau can disrupt mitochondrial dynamics and bioenergetics (Cheng and Bai, 2018; Guha et al, 2020; Quintanilla et al, 2020; Szabo et al, 2020; Tracy et al, 2022). The SCLA1A2/EAAT2 enrichment is particularly striking given prior evidence that EAAT2 dysfunction reduces glutamate clearance in AD and contributes to excitotoxic stress, which is a known association between soluble tau aggregates and glutamate-handling machinery, providing a mechanistic route linking tau conformers to synaptic hyperexcitability and metabolic stress (Cheng and Bai, 2018; Tracy et al, 2022).

PBS-soluble AD tau is also associated with the SNARE complex proteins and syntaxins, implicating misfolded tau in the presynaptic vesicle mechanism. Recent interactomes and functional studies map tau binding to presynaptic vesicle proteins and show syntaxins, which can facilitate tau secretions, known mechanisms that may underlie trans-synaptic spread and synaptic dysfunction in tauopathies (Tracy et al, 2022). As such, our data complements other studies showing that misfolded tau may impair energy supply, altering neurotransmitter handling and presynaptic release, producing a convergent synaptic/metabolic vulnerability in AD.

Enrichment of antioxidant enzymes (TXN, PRDX1, SOD1/SOD2) and astrocytic markers with AD tau supports the model, which suggests that misfolded tau may perturb neuron-glia metabolic coupling and antioxidant defenses, a phenomenon also seen with Aβ (Norambuena et al, 2022). Oxidative stress is well-documented in AD and promotes tau pathology and arises as a downstream consequence of mitochondrial dysfunction; the detected direct interaction suggests that misfolded tau may amplify oxidative damage with its association with redox machinery (Du et al, 2022; Houldsworth, 2024).

Comparatively, PSP tau displayed a strikingly different profile where metabolic and many neurotransmission-associated proteins were depleted from PSP tau (both fractions), while proteostasis-related proteins, particularly PSMB7 and the E3 TRIM21-selectively associated compared to AD or DLB. Consistent with this, PSP phosphorylated tau proximity proteomics independently highlighted enrichment of protein-degradation and stress-response networks alongside cytoskeletal interactors (Radford et al, 2023). Of note, PLA confirmed direct interaction with PSMB7 and tau. Although group-wise differences were not statistically significant, the direction and effect sizes were consistent with the interactome data generated through LC-MS/MS. One possible explanation for this is that our LC-MS/MS derives from T18 immunocapture of PBS- and sarkosyl-soluble fractions, whereas PLA was performed on fixed tissue sections. Fractionation followed by immunocapture enriches misfolded tau containing complexes and boosts signal to noise ratio, while PLA identifies the full cellular milieu, diluting events confined to specific fractions. TRIM21 has been shown to neutralize cytosolic tau seeds by recruiting proteasomal and p97/VCP activity, offering a possible mechanistic interpretation for its PSP association in our dataset (Malpetti et al, 2021; Mukadam et al, 2023; Radford et al, 2023). This proteostasis-dominant interactome distinguishes PSP from the metabolic/synaptic signature of AD.

DLB tau interactors clustered around vesicle docking, synaptic organization and proteins implicated in neuronal remodeling/neurogenesis (e.g., BAIAP2, GAS7, and APOE). The depletion of inflammatory mediators (S100A8/A9), MT3 from DLB tau, contrasts with AD. This pattern fits a broader observation where S100A8/A9 positive microglia are enriched in primary tauopathies and AD, as well as accelerated innate immune aging, while postmortem and in vivo data suggest that robust microglial activation in DLB is often tied to concomitant AD pathology rather than pure DLB (Amin et al, 2020; Gruel et al, 2024; Wetering et al, 2024). MT3, frequently reduced in AD and central to neuronal metal homeostasis, has also been linked to α-Syn biology via metalothionien–metal interactions, offering a possible explanation for disease-specific differences in MT3 engagement with tau (Calvo et al, 2020; Meloni and Vasak, 2011; Tsuji et al, 1992; Yu et al, 2001; Zuo et al, 2009). Given the frequent copresence and interaction between tau and α-Syn pathologies, the synaptic/vesicular tilt of the DLB-derived tau interactome may capture pathways by which pathologies disrupt presynaptic trafficking and neuronal connectivity. One possible explanation may be that α-Syn is concentrated at the presynaptic terminals and pathological increases perturb synaptic vesicular cycling, which is elevated in DLB cohorts (Burre et al, 2010; Calabresi et al, 2023; Fouke et al, 2021; Maroteaux et al, 1988; Wang et al, 2014; Williams et al, 2020). This further explains our tau-centric synaptic/vascular network.

Ubiquitination emerged as the most disease-specific PTM as PSP tau displayed globally elevated ubiquitination and selective enrichment for proteasomal components, notably TRIM21. This is consistent with previously published works investigating PSP-phospho-tau proximity proteomics that show strong representation of ubiquitin proteosome pathways and with mechanistic works that established that TRIM21 can neutralize intracellular seeds (McEwan et al, 2017; Mukadam et al, 2023; Radford et al, 2023). By contrast, AD tau preferentially associated with cullin-RING ligase components (SKP1, CUL3, and CAND1), which aligns with previously published reports of cullin-RING ligase components being activated by neddylation and that neddylation/CRL signaling is perturbed in AD brain (Chen et al, 2012; Zhang et al, 2024). We also report enrichment of ubiquitin E3/E4 factors with AD tau, including MARCH9 and UBE4B. MARCH9 functions as a RING-CH E3, which ubiquitinates membrane proteins to route them to the lysosomes. To our knowledge, direct tau ubiquitination by MARCH9 has not been reported; as such, its enrichment in our data extends prior MARCH family involvement beyond MARCH7, which has been reported to ubiquitinate tau and reduce microtubule binding (Flach et al, 2014). Interestingly, UBE4B cooperates with CHIP to extend the tau ubiquitin chains and promote clearance (Subramanian et al, 2021), providing a potential explanation for their enrichment in AD. Conversely, we also observed the lowest overall tau ubiquitination and minimal E3 enrichment relative to AD/PSP.

Mechanistically, our data fits a PTM-cross talk model in which disease-specific ubiquitin E3 sculpt tau’s lysine usage and routing. Tau acetylation at key microtubule-binding lysines play a crucial role in microtubule binding, promoting aggregation, and functionally competes with lysine ubiquitination (Cohen et al, 2011; Ferreon et al, 2018; Min et al, 2010; Yan et al, 2022). Conversely, UBE4B ubiquitination has been reported to suppress aggregation and favor degradation (Subramanian et al, 2021). This fundamentally suggests that E3 interaction and PTM patterning are integral to tau polymorph identity, aggregation and life span of the aggregates (proteosome vs autophagy) (Li et al, 2021; Yan et al, 2022). To add, we did not detect phosphorylation at the AT8 epitope (pSer202/pThr205); however, pSer199 was ~8-fold more abundant than pSer202 in our dataset. The absence of AT8-site phosphorylation, together with the relative enrichment of pSer199, suggests that the misfolded tau isolated from AD, PSP, and DLB reflects an early pathological stage. pSer199 has been reported as an early marker of tau pathology and is detectable in pretangle neurons, preceding robust AT8 positivity (Christensen et al, 2023; Maurage et al, 2001, 2003; Neddens et al, 2018).

Several limitations should be considered when interpreting these findings. The present work constitutes a correlative resource generated from human postmortem brain and the associations between disease state, inferred tau conformation differences, and the interactome. The present work does not address the mechanistic implications of the observed interactions. Further studies will be required to establish causality and to elucidate the functional consequences of these disease-specific interactions. Beyond these interpretive considerations, several methodological limitations should be acknowledged. First, the discovery cohort comprised three cases per disease group; although this constraint reflects the limited availability of well-characterized, fraction-grade postmortem tissue, we sought to mitigate it by validating the principal disease-specific interactors in an independent cohort of five cases per group using targeted PRM-MS (Fig. EV3; Dataset EV6). Second, our analysis was restricted to the frontal cortex in both cohorts. Because tau pathology and its regional vulnerability differ across tauopathies, the interactomes reported here may not fully recapitulate those of other affected regions; the frontal cortex was selected because it is consistently involved across AD, PSP, and DLB, permitting a direct cross-disease comparison. Third, misfolded tau was captured with a single conformation-specific antibody (T18), which recognizes a subset of aggregated tau conformers and could therefore bias the recovered interactome (Arar et al, 2025; Kadamangudi et al, 2024; Lo Cascio et al, 2020; Lo Cascio et al, 2025; Marcatti et al, 2025; Puangmalai et al, 2024; Puangmalai et al, 2022; Ruan et al, 2021; Samelson et al, 2026; Scaduto et al, 2024). Reassuringly, key interactions were independently confirmed in tissue with an orthogonal total-tau antibody by PLA and by western and dot blot analysis. Nevertheless, future studies employing complementary capture strategies will be needed to recover the full breadth of the misfolded tau interactome.

In summary, our findings show that tau polymorph conformations are associated with disease-specific interaction networks and PTM states, providing molecular insight into tauopathy heterogeneity.

## Methods

**Table:** Reagents and tools table

Reagent/resource	Reference or source	Identifier or catalog number
**Experimental models**		
Postmortem human brain tissue: Alzheimer’s disease (AD), Progressive Supranuclear Palsy (PSP), Dementia with Lewy Bodies (DLB), and control cases	University of Kentucky Alzheimer’s Disease Center Tissue Bank	N/A
Postmortem human brain tissue: AD, PSP, DLB, and control cases	Oregon Brain Bank	N/A
Postmortem human brain tissue: AD, PSP, DLB, and control cases	Michigan Brain Bank	N/A
Postmortem human brain tissue: AD, PSP, DLB, and control cases	Brain Resource Center at Johns Hopkins University	N/A
**Recombinant DNA**		
N/A		
**Antibodies**		
Mouse anti-Tau 13 (TAU13)	BioLegend	Cat# 835201**;** RRID:AB_2565341
Rabbit anti-T18 (misfolded tau-specific)	(Arar et al, 2025; Kadamangudi et al, 2024; Lo Cascio et al, 2020; Lo Cascio et al, 2025; Marcatti et al, 2025; Puangmalai et al, 2024; Puangmalai et al, 2022; Ruan et al, 2021; Samelson et al, 2026; Scaduto et al, 2024)	N/A (In-house)
Rabbit anti-FABP5	ProteinTech	Cat# 12348-1-AP; RRID:AB_2100341
Rabbit anti-SLC1A2 (EAAT2)	Abcam	Cat# ab41621; RRID:AB_941782
Rabbit anti-FH (fumarate hydratase)	ProteinTech	Cat# 11375-1-AP; RRID:AB_763171
Rabbit anti-PSMB7 (E1L5H)	Cell Signaling Technology	Cat# 13207; RRID:AB_2798149
Rabbit anti-UBE4B (EPR7471)	Abcam	Cat# ab126759; RRID:AB_11144331
Rabbit anti-MARCH9	Invitrogen	Cat# PA5-103817; RRID:AB_2853150
Rabbit anti-Skp1	Cell Signaling Technology	Cat# 2156; RRID:AB_2270271
Rabbit anti-UBA52 (EPR4546)	Abcam	Cat# ab109227; RRID:AB_10861393
Mouse anti-MARS (MetRS) (MARSD10B4)	Abcam	Cat# ab50793; RRID:AB_881492
Rabbit anti-MAGT1	ProteinTech	Cat# 17430-1-AP; RRID:AB_2138982
Rabbit anti-GAPDH (loading control)	Abcam	Cat# ab9485; RRID:AB_307275
Chicken anti-MAP2 (neuronal marker)	Abcam	Cat# ab5392; RRID: AB_2138153
Goat anti-Chicken IgY (H + L), Alexa Fluor 488 (secondary)	Abcam	Cat# ab150169; RRID:AB_2636803
IRDye 680RD Goat anti-Mouse IgG (H + L)	LI-COR	Cat# 926-68070; RRID:AB_10956588
IRDye 680RD Goat anti-Rabbit IgG (H + L)	LI-COR	Cat# 926-68071; RRID:AB_10956166
IRDye 800CW Goat anti-Mouse IgG (H + L)	LI-COR	Cat# 926-32210; RRID:AB_621842
IRDye 800CW Goat anti-Rabbit IgG (H + L)	LI-COR	Cat# 926-32211; RRID:AB_621843
**Oligonucleotides and other sequence-based reagents**		
N/A		
**Chemicals, enzymes and other reagents**		
cOmplete Protease Inhibitor Cocktail tablets	Roche (Sigma-Aldrich)	Cat# 04693116001
Pierce BCA Protein Assay Kit	Thermo Scientific	Cat# 23225
Dynabeads Protein A Immunoprecipitation Kit	Invitrogen (Thermo Fisher Scientific)	Cat# 10006D
Duolink In Situ Red Starter Kit Mouse/Rabbit (PLA)	Sigma-Aldrich	Cat# DUO92101
NuPAGE 4–12% Bis-Tris Mini Protein Gels, 1.0–1.5 mm	Invitrogen	Cat# NP0321BOX
Odyssey Blocking Buffer (PBS)	LI-COR	Cat# 927-40000
Sequencing-grade modified trypsin	Promega	Cat# V5111
Ammonium bicarbonate (ABC)	Sigma-Aldrich	Cat# A6141
2,2,2-Trifluoroethanol (TFE)	Sigma-Aldrich	Cat# T63002
Acetic acid, ACS grade	Sigma-Aldrich	Cat# 695092
Iodoacetamide	Thermo Scientific	Cat# 122271000
Dithiothreitol (DTT)	Thermo Scientific	Cat# R0862
Acetonitrile (ACN), LC/MS grade	Thermo Scientific	Cat# 047138.K2
Formic acid, LC/MS grade	Thermo Scientific	Cat# 28905
Methanol, LC/MS grade	Thermo Scientific	Cat# 047192.K2
Urea ultra	MP Biomedicals	Cat# 0210569501
10X Phosphate-buffered saline	Corning	Cat# 46-013-CM
Sarkosyl (N-Lauroylsarcosine sodium salt)	Sigma-Aldrich	Cat# 61747
Glycine	Fisher BioReagents	Cat# BP381-5
Tris-HCl	Sigma-Aldrich	Cat# T2694
Bovine Serum Albumin (BSA)	Sigma-Aldrich	Cat# A4503
**Software**		
MaxQuant (v1.5.2.8) with Andromeda search engine	(Cox et al, 2014; Cox and Mann, 2008)	RRID:SCR_014485
Perseus (v1.6.15.0)	(Tyanova et al, 2016)	RRID:SCR_015753
Cytoscape (v3.10.1)	(Shannon et al, 2003)	RRID:SCR_003032
Cytoscape StringApp	(Doncheva et al, 2019)	RRID:SCR_025009
EnrichmentMap (Cytoscape plugin)	(Merico et al, 2010)	RRID:SCR_016052
Gene Ontology Biological Process (GOBP) database	Gene Ontology Consortium	RRID:SCR_002811
KEGG (Kyoto Encyclopedia of Genes and Genomes)	(Kanehisa and Goto, 2000)	RRID:SCR_012773
Reactome	(Jassal et al, 2020)	RRID:SCR_003485
ImageJ	NIH/(Schneider et al, 2012)	RRID:SCR_003070
Skyline (v3.6)	(MacLean et al, 2010)	RRID:SCR_014080
Xcalibur	Thermo Fisher Scientific	RRID:SCR_014593
GraphPad Prism (v10.0)	GraphPad Software	RRID:SCR_002798
UniProtKB/SwissProt human database (released December 8, 2015; 42,130 entries)	UniProt Consortium	RRID:SCR_002380
BioRender	https://BioRender.com/g5zphob https://BioRender.com/vba9ahx https://BioRender.com/d7pyji0 https://BioRender.com/wi2ieka	RRID:SCR_018361
**Other**		
Easy nLC1000 nanoflow UHPLC system	Thermo Fisher Scientific	RRID:SCR_014993
Q Exactive Orbitrap mass spectrometer	Thermo Fisher Scientific	RRID:SCR_020565
Nanoelectrospray ion source	Thermo Fisher Scientific	ES071
C18-reversed-phase column	Thermo Fisher Scientific	Cat# 164568
Olympus SpinSR-10 Yokogawa spinning disk confocal	Olympus	N/A
UIS2XLine UPLAPO 60× oil objective (NA 1.5)	Olympus	N/A
3.2× MagChanger	Olympus	N/A
ORCA Fusion sCMOS camera	Hamamatsu	Cat# C14440-20UP
Odyssey CLx Near-Infrared Imaging System	LI-COR	Cat# 9140
SpeedVac centrifugal concentrator	Thermo Fisher Scientific	Cat# DNA130-230

### Brain homogenate preparation

Postmortem brain tissue samples from AD, PSP, and DLB subjects were obtained from the University of Kentucky Alzheimer’s Disease Center Tissue Bank, Oregon Brain Bank, Michigan Brain Bank, and the Brain Resource Center at Johns Hopkins and approved by the Institutional Ethics Committee. Neuropathological assessment conformed to National Institute on Aging/Reagan Institute consensus criteria. The following information was available for the cases used in this study: diagnosis, age at death, sex, postmortem index, brain area, and Braak stage (Table 1). For independent validation, a separate cohort of frontal cortex tissue from control, AD, PSP, and DLB cases (*n* = 5 per disease group) was obtained under the same neuropathological criteria, with case information provided in Table 1B.

### Separation of soluble and insoluble tau

Separation of PBS-soluble and -insoluble tau was performed in accordance with protocols previously described (Gerson et al, 2014; Lasagna-Reeves et al, 2010; Lasagna-Reeves et al, 2012a; Lasagna-Reeves et al, 2012b; Puangmalai et al, 2024; Puangmalai et al, 2020; Puangmalai et al, 2022; Sengupta et al, 2015). In brief, brain tissues and cell lysates were homogenized in 1X phosphate-buffered saline (PBS) with protease inhibitor (Roche) at 1:3 (w/v) ratio and incubated for 20 min at 4 °C. Then the samples were centrifuged at 11,000×*g* for 20 min at 4 °C. The supernatant was collected and centrifuged at 100,000×*g* for 60 min at 4 °C. To extract the PBS-insoluble tau, the pellets from the first and second cold centrifugation were combined and resuspended in PHF extraction buffer (10 mM Tris-HCl, 1 mM EGTA, 0.85 M NaCl, 10% (w/v) sucrose, pH 7.4) at a 1:10 (w/v) ratio. Samples were centrifuged at 15,000×*g* for 20 min at 4 °C. Sarkosyl solution was added to the supernatant at a final concentration of 1% and stirred for 1 h at room temperature (RT) before centrifugation at 100,000×*g* for 30 min at 4 °C. The supernatant was collected as a sarkosyl-soluble fraction and used for downstream applications. Finally, the sarkosyl-insoluble fraction was made by resuspending the pellet in 1X PBS, depending on the amount of starting material (1 ml buffer for 25 g of starting material). The total protein concentrations of the final fractions were analyzed by bicinchoninic acid (BCA) assay (Thermo Scientific).

### Immunoprecipitation (IP)

PBS-soluble and sarkosyl-soluble tau were immunoprecipitated with a misfolded tau-specific T18 antibody (Arar et al, 2025; Kadamangudi et al, 2024; Lo Cascio et al, 2020; Lo Cascio et al, 2025; Marcatti et al, 2025; Puangmalai et al, 2024; Puangmalai et al, 2022; Ruan et al, 2021; Samelson et al, 2026; Scaduto et al, 2024) using Dynabead Protein A kit (Invitrogen#1006D). Briefly, amine-reactive resin was coupled with affinity-purified T18 antibody, incubated with samples. Bound proteins were eluted in 0.1 M glycine (pH 2.8), and the pH was adjusted to 7.0 by adding 1 M Tris-HCl (pH 8). Isolated fractions were subjected to buffer exchange against 1X PBS, followed by LC-MS/MS and dot blot analysis. The total protein concentration was measured with a BCA assay.

### In situ proximity ligation assay (PLA)

To identify the interaction of tau and its interactors in postmortem human brain tissues, the Duolink In Situ Red starter kit mouse/rabbit (Sigma-Aldrich; DUO92101) was used according to the manufacturer’s guidelines. Briefly, frozen human brain tissues with AD, PSP, and DLB were pretreated with chilled methanol before incubating with blocking solution at 37 °C. After 1 h, the primary antibody solution was added overnight at 4 °C. Primary antibodies used include mouse anti-Tau 13 (1:20000, BioLegend; MMS-520R) and rabbit anti-interactors, including anti-FABP5 (1:2000, ProteinTech, 12348-1-1AP), anti-SLC1A2 (1:1000, Abcam, ab41621), anti-FH (1:1000, ProteinTech, 11375-1-AP), anti-PSMB7 (1:1000, Cell Signaling Technology, 13207), anti-UBE4B (1:1000, Abcam, ab126759), anti-MARCH9 (1:1000, Invitrogen, PA5-103817), anti-Skp1 (1:1000, Cell Signaling Technology, 2156), and anti-UBA52 (1:1000, Abcam, ab109227). After two washes for 5 min with 1X wash buffer A, the PLA probe solution was applied for 1 h at 37 °C. Tissues were washed and incubated with ligation buffer for 30 min at 37 °C, followed by washing and adding the amplification solution for 100 min at 37 °C. Subsequently, the sections were incubated with the anti-MAP2 (1:1000, Abcam; ab5392) diluted in 5% BSA in PBST overnight at 4 °C. After three washes with 1X PBS, the sections were incubated with Alexa-conjugated secondary antibody (1:700, ab150169) diluted in 5% BSA in PBST for 1 h at RT. Slides were then washed 10 min twice with 1X washing buffer B at RT before mounting using Duolink In Situ Mounting Medium with DAPI.

### Confocal image analysis

Brain sections were imaged with UIS2XLine Plan Apochromat (UPLAPO) 63×oil objective (NA 1.5, Olympus) and a 3.2× MagChanger of an Olympus SpinSR-10 Yokogawa spinning disk confocal through an ORCA Fusion sCMOS camera (Hamamatsu). To build the z-stack, 17 stacks/0.37–0.41-μm optimal thickness was captured. Each brain section was randomly imaged in three different regions of interest (Montalbano et al, 2020; Puangmalai et al, 2022). Laser power was set using secondary antibody–only controls. All images were analyzed using ImageJ (NIH). Each image was converted into an 8-bit greyscale for each channel. The channel containing the PLA signal was thresholded until the PLA puncta were reliably isolated from the background, forming a binary image. Overlapping PLA puncta were segmented using a watershed function and over/undersized puncta were excluded by defining the expected size range of PLA puncta. The summary result of PLA puncta/field was reported (George et al, 2022; Romero-Fernandez et al, 2023). From each frame, five region of interests (ROIs) have been randomly selected and quantified using the method described. Data collection includes PLA puncta number, area and fluorescent intensities. The number of puncta has been plotted using GraphPad.

### Western blot and dot blot analysis

For Western blot analysis, an equal proportion of PBS- or sarkosyl-soluble fractions from human brain homogenate and IP with T18 materials were loaded on precast NuPAGE 4 to 12% Bis-Tris gels (Invitrogen) for SDS-PAGE analysis. Gels were subsequently transferred onto nitrocellulose membranes and blocked for 1 h at RT using Odyssey Blocking Buffer (LI-COR). For dot blot, an equal amount of IP-T18 samples were dotted on the membrane and let dry for 1 h at RT, followed by blocking. Membranes were then probed overnight at 4 °C using the corresponding primary antibodies, followed by IRdye secondaries (LI-COR) at 1:10,000 for 1 h at RT. Images were acquired by the LI-COR Odyssey imager. Densitometric analysis was performed using ImageJ software (NIH). The primary antibodies used were the following: mouse anti-Tau 13 (1:20,000, BioLegend; 835204), rabbit anti-T18 (1:2000), rabbit anti-FABP5 (1:2000, ProteinTech, 12348-1-1AP), rabbit anti-SLC1A2 (1:1000, Abcam, ab41621), rabbit anti-FH (1:1000, ProteinTech, 11375-1-AP), rabbit anti-PSMB7 (1:1000, Cell Signaling Technology, 13207), rabbit anti-UBE4B (1:1000, Abcam, ab126759), rabbit anit-MARCH9 (1:1000, Invitrogen, PA5-103817), rabbit anti-Skp1 (1:1000, Cell Signaling Technology, 2156), rabbit anti-UBA52 (1:1000, Abcam, ab109227), mouse anti-MARS (1:2000, Abcam, ab50793), rabbit anti-MAGT1 (1:1000, ProteinTech, 17430-1-AP), and rabbit anti-GAPDH antibody (1:1000, Abcam; ab9485).

### Mass spectrometry

**Regents and chemicals**—all reagents were ACS grade or higher. All solvents used, including water, were LC/MS grade. Ammonium bicarbonate (ABC), 2,2,2-trifluoroethanol (TFE), and acetic acid were purchased from Sigma-Aldrich. Iodoacetamide (IDA), dithiothreitol (DTT), acetonitrile (ACN), formic acid, and methanol were purchased from Thermo Scientific. Urea ultra was from MP Biomedicals. Sequencing-grade modified trypsin (Promega) was used.

### Trypsin digestion

The trypsin digestion was performed as previously described (Tian et al, 2016; Yang et al, 2015). About 100 µL of 50 mM ammonium bicarbonate was added to each sample. The beads were suspended with gentle vortexing for 1 h. The proteins on the beads were reduced using 10 mM DTT for 30 min, then alkylated with 20 mM IDA for 1 h in the dark. An aliquot of 4 µg of sequencing-grade trypsin was added to each sample before a 4 h incubation at 37 °C with gentle shaking; the supernatant was then collected. Another 4 µg of trypsin was then added to the beads, and the sample was incubated at 37 °C overnight with gentle shaking; the supernatant was then collected. After trypsin digestion, the beads were washed twice with 50 µL of 50% ACN, and the supernatants were collected. All the supernatants were combined and dried with a SpeedVac.

### LC-MS/MS analysis

A nanoflow ultra-high performance liquid chromatography (UHPLC) instrument (Easy nLC, Thermo Fisher Scientific) was coupled online to a Q Exactive mass spectrometer (Thermo Fisher Scientific) with a nanoelectrospray ion source (Thermo Fisher Scientific). Peptides were loaded onto a C18-reversed-phase column (25 cm long, 75-μm inner diameter) and separated with a linear gradient of 5–35% buffer B (100% acetonitrile in 0.1% formic acid) at a flow rate of 300 nL/min over 120 min. Each sample was analyzed by LC-MS/MS twice. MS data were acquired using a data-dependent Top15 method, dynamically choosing the most abundant precursor ions from the survey scan (350–1600 m/z) using HCD fragmentation. Survey scans were acquired at a resolution of 70,000 at m/z 400. Unassigned precursor ion charge states, as well as singly charged species, were excluded from fragmentation. The isolation window was set to 3 Da and fragmented with normalized collision energies of 28. The maximum ion injection times for the survey scan and the MS/MS scans were 20 and 120 ms, respectively, and the ion target values were set to 3E6 and 1e5, respectively. Selected sequenced ions were dynamically excluded for 30 s. Data were acquired using Xcalibur software.

### Data processing and bioinformatic analysis

Raw MS data were analyzed using MaxQuant software version 1.5.2.8 using the Andromeda search engine (Cox et al, 2014; Cox and Mann, 2008). The initial maximum allowed mass deviation was set to 10 ppm for monoisotopic precursor ions and 20 ppm for MS/MS peaks. Enzyme specificity was set to trypsin, defined as C-terminal to arginine and lysine, excluding proline, and a maximum of two missed cleavages was allowed. Carbamidomethylcysteine was set as a fixed modification, methionine oxidation, ubiquitination of lysine, acetylation of lysine, phosphorylation of serine, threonine, and tyrosine as variable modifications. The spectra were searched with the Andromeda search engine against the SWISSPROT (Database released on December 8, 2015) human sequence database (containing 42,130 human protein entries), combined with 248 common contaminants and concatenated with the reversed versions of all sequences. Protein identification required at least one unique or razor peptide per protein group. Quantification in MaxQuant was performed using the built-in XIC-based label-free quantification (LFQ) algorithm (Cox et al, 2014). The required false positive rate for identification was set to 1% at both the peptide and protein level, and the minimum required peptide length was set to eight amino acids.

For statistical analysis of MaxQuant output, we used the Perseus platform (Version 1.6.15.0) (Tyanova et al, 2016). Contaminants, reverse identification, and proteins only identified by site were excluded from further data analysis. The LFQ values were log_2_-transformed. After removing the proteins with less than three valid LFQ values in total, the remaining missing LFQ values were imputed from a normal distribution (width, 0.3; down shift, 1.8).

### Network analysis and visualization

The significant proteins were imported to Cytoscape (Version 3.10.1) (Lewandrowski et al, 2024) for network analysis and annotation enrichment analysis. The visualization and functional enrichment analysis of the protein–protein interaction network of soluble tau was conducted using Cytoscape StringApp (Doncheva et al, 2023).

Gene Ontology term enrichment analyses were performed using the EnrichmentMap tool (Reimand et al, 2019). Enrichment and network analyses were applied from Gene Ontology Biological Process (GOBP), Kyoto Encyclopedia of Genes and Genomes (KEGG), and Reactome. Benjamini-Hochberg false discovery rate below 5% was used as the cutoff for significant terms.

### Parallel reaction monitoring-mass spectrometry (PRM) Analysis of tau and disease-specific tau interactors

For PRM analyses, the peptides were analyzed with an Easy nLC1000 UHPLC-Q Exactive Orbitrap LC-MS system (Thermo Scientific, San Jose, CA). The resolution of the full scan was 70,000 (@m/z 200), the target automatic gain control value was set to 3 × 10^6^, and the maximum fill time was 200 ms for full scan; and 17,500 (@m/z 200), a target automatic gain control value of 2 × 10^5^, and maximum fill times of 100 ms for MS2 scan. PRM targeted ubiquitinated tau peptides. The assessment of the detection of peptides was performed after acquisition using Skyline version 3.6 (Thomas and Yang, 2017). For each peptide evaluated, the signals of the 3-5 most intense fragment ions were extracted from each corresponding MS/MS spectrum library of these ubiquitinated tau peptides. The MS/MS spectra with five fragment ions detected were submitted to spectral matching. The comparison of the relative intensities of these fragments with those defined in the reference composite MS/MS spectrum was performed on the basis of the dot product (dotp) value. The same PRM-MS workflow was applied to the independent validation cohort to quantify the disease-specific tau interactors that best discriminated control, AD, PSP, and DLB in the classification analysis (Table 1), monitoring the three to five most intense fragment ions per target peptide (the signature peptides for each protein were listed in Dataset EV6). When multiple peptides were analyzed for a protein, the sum of the peptides’ intensities was calculated as the protein intensity. The intensity of each protein was normalized with total tau intensity.

### Statistical analysis

Statistical analyses were performed using Prism 10.0 (GraphPad Software) through unpaired two-tailed Student’s *t*-test or one-way analysis of variance (ANOVA) according to group number. Results are considered statistically significant at *p* < 0.05.

## Supplementary information

Peer Review File

Dataset EV1

Dataset EV2

Dataset EV3

Dataset EV4

Dataset EV5

Dataset EV6

Source data Fig. 1

Source data Fig. 2

Source data Fig. 3

Source data Fig. 6

Source data Fig. 7

Figure EV1 Source Data

Figure EV2 Source Data

Figure EV3 Source Data

Figure EV4 Source Data

Expanded View Figures

## Data Availability

All data are contained within this manuscript and supporting information. The datasets used during the current study are available from the corresponding authors upon request. The mass spectrometry proteomics data have been deposited to the ProteomeXchange Consortium (Deutsch et al, 2026) via the PRIDE (Perez-Riverol et al, 2025) partner repository with the dataset identifiers PXD079888 (https://www.ebi.ac.uk/pride/archive/projects/PXD079888/public) and PXD079991 (https://www.ebi.ac.uk/pride/archive/projects/PXD079991/public). The source data of this paper are collected in the following database record: biostudies:S-SCDT-10_1038-S44318-026-00897-8.
