# Supplementary material for: Disease-specific tau polymorphs are associated with unique protein networks across proteinopathies
Source: EMBO J. 2026 Aug 18;45(19):6946–77. doi: 10.1038/s44318-026-00897-8 (PMC13631283; doi:10.1038/s44318-026-00897-8)
Supplement: Supplementary file 1 [file 44318_2026_897_MOESM17_ESM.pdf]

Expanded View Figures

Figure EV1. Related to Fig. 1. Comparison of tau interactomes with the brain proteome and validation of selected proteins.

(**A**) Total MAPT signal intensity across PBS-soluble and sarkosyl-soluble fractions in AD (red), PSP (blue), and DLB (green) cases ($n = 3$ independent cases per group) was detected by label-free LC-MS/MS. The adjusted p values are as follows: PBS-soluble AD tau vs sarkosyl-soluble AD tau ($p < 0.0001$), PBS-soluble DLB tau vs sarkosyl-soluble DLB tau ($p < 0.0001$), sarkosyl-soluble AD tau vs PSP tau ($p = 0.0006$), sarkosyl-soluble PSP tau vs DLB tau ($p = 0.0012$). (**B**) Domain map of the full-length human MAPT with magenta letters marking the positions of representative tryptic peptides plotted for PBS-soluble tau (**C–I**). (**C–I**) Normalized Log2 LFQ intensities for representative tau peptides from PBS-soluble fractions across AD, PSP, and DLB ($n = 3$ independent cases per group). The adjusted p values are as follows: (**C**) $_{68}$STPTAEDVTAPLVDEGAPGK$_{87}$; AD tau vs DLB tau ($p = 0.0406$), (**D**) $_{156}$GAAPPGQKGQANATR$_{170}$; AD tau vs DLB tau ($p = 0.0070$), PSP tau vs DLB tau ($p = 0.0070$), (**E**) $_{260}$IGSTENLKHQPGGGK$_{274}$; AD tau vs DLB tau ($p = 0.0331$), (**F**) $_{268}$HQPGGGKVQIINK$_{280}$; AD tau vs PSP tau ($p = 0.0094$), (**H**) $_{299}$HVPGGGSVQIVYKPVDLSK$_{317}$; AD tau vs DLB tau ($p = 0.0306$), (**I**) $_{354}$IGSLDNITHVPGGGNKK$_{370}$; AD tau vs PSP tau ($p = 0.0317$). (**J**) MAPT domain map with peptide positions for sarkosyl-soluble tau (**K**, **L**). (**K**, **L**) Normalized Log2 LFQ intensities for representative tau peptides from sarkosyl-soluble fractions across AD, PSP, and DLB ($n = 3$ independent cases per group). The adjusted p values are as follows: (**K**) $_{195}$SGYSSPGSPGTPGSR$_{209}$; AD tau vs PSP tau ($p = 0.0059$), PSP tau vs DLB tau ($p = 0.0244$). (**M**) Scatter plots comparing Log2 fold change (FC) in the tau interactome (y-axis) versus Log2 FC in total PBS-soluble lysate (x-axis) for PBS-soluble fractions from AD, PSP, and DLB brains. Each point is a protein significantly detected in T18 immunoprecipitations; gray crosshairs mark zero on both axes. (**N**) Scatter plots comparing Log2 FC in the misfolded tau interactome (y-axis) versus Log2 FC in total sarkosyl-soluble lysate (x-axis) for sarkosyl-soluble fractions from AD, PSP, and DLB brains. Each point is a protein significantly detected in T18 immunoprecipitations; gray crosshairs mark zero on both axes. (**O–Q**) Representative immunoblots and quantification of FABP5 and FH normalized to GAPDH in total PBS-soluble (top) and sarkosyl-soluble (bottom) from AD, PSP, and DLB cases ($n = 3$). Blue dots, PBS-soluble; red dots, sarkosyl-soluble. Quantification of band intensity was normalized to GAPDH (mean ± SEM). The same immunoblots probed with loading controls are shown in Figs. 6. and EV3. Statistical analyses were calculated by two-way ANOVA with the Tukey test. Asterisks indicate pairwise differences among diseases within a fraction; hash marks indicate PBS- vs sarkosyl-soluble differences within the same disease (*$p < 0.05$, **$p < 0.01$, ***$p < 0.001$, ****$p < 0.0001$; $^{\#}p < 0.05$, $^{\#\#}p < 0.01$, $^{\#\#\#}p < 0.001$, $^{\#\#\#\#}p < 0.0001$).Source data are available online for this figure.

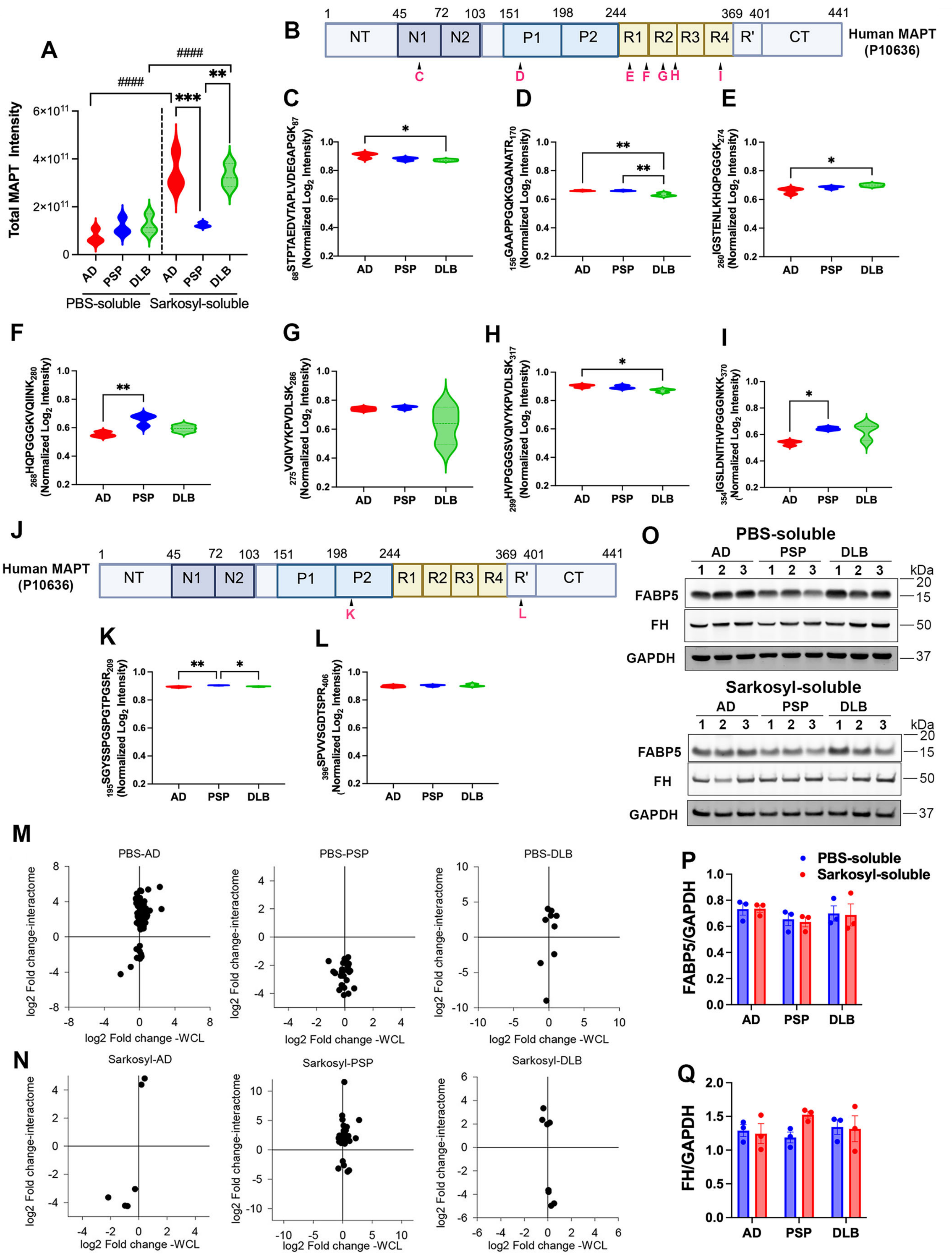
A
B
1 45 72 103 151 198 244 369 401 441
NT N1 N2 P1 P2 R1 R2 R3 R4 R' CT
Human MAPT (P10636)
C D E F G H I
Total MAPT Intensity
PBS-soluble
Sarkosyl-soluble
AD PSP DLB
C
D
E
F
G
H
I
J
Human MAPT (P10636)
K L
K
L
M
PBS-AD
PBS-PSP
PBS-DLB
log2 Fold change-interactome
log2 Fold change -WCL
N
Sarkosyl-AD
Sarkosyl-PSP
Sarkosyl-DLB
O
PBS-soluble
Sarkosyl-soluble
FABP5
FH
GAPDH
kDa
P
PBS-soluble
Sarkosyl-soluble
FABP5/GAPDH
Q
FH/GAPDH

 © The Author(s)

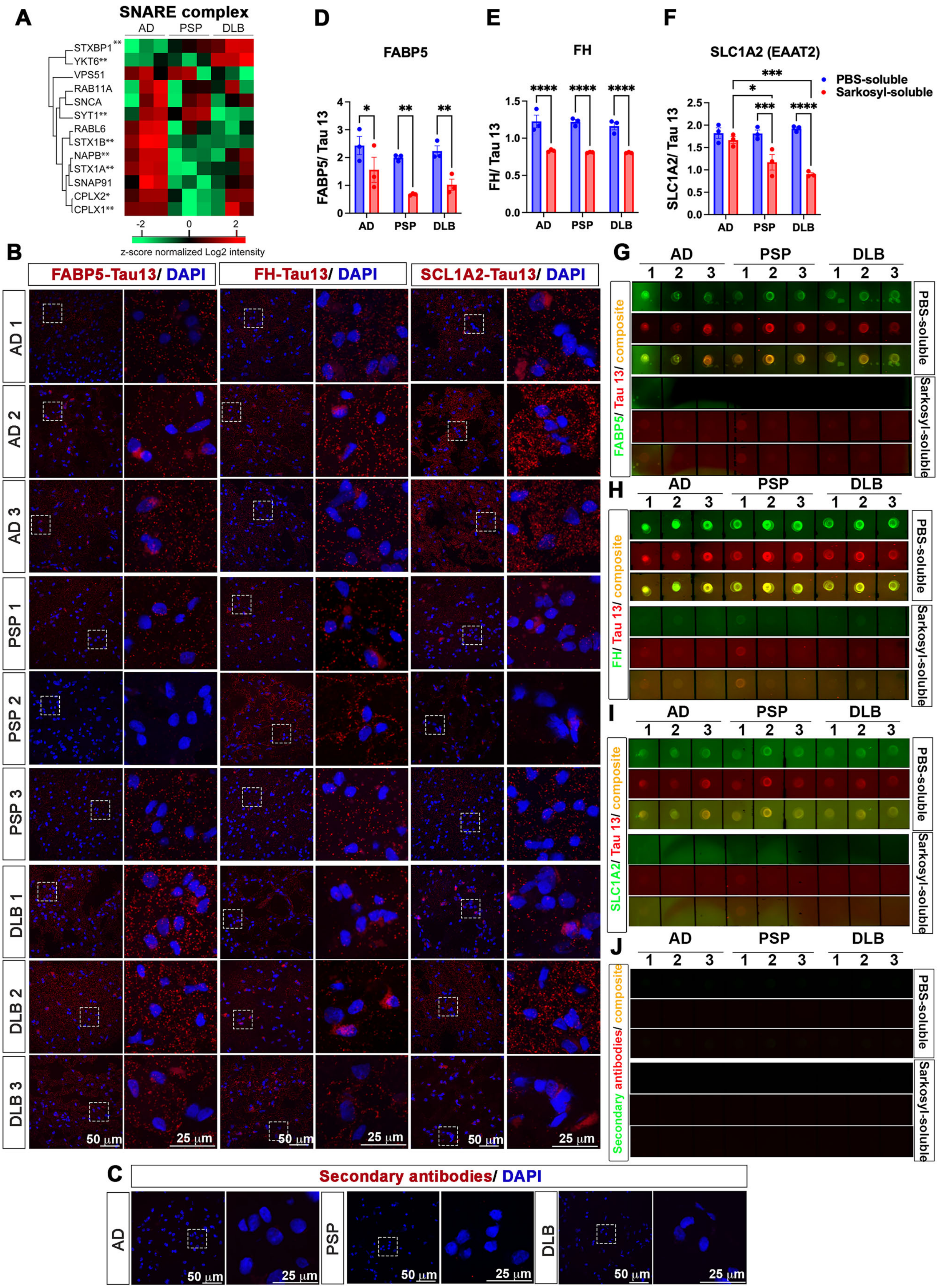
A
SNARE complex
AD PSP DLB
STXBP1**
YKT6**
VPS51
RAB11A
SNCA
SYT1**
RABL6
STX1B**
NAPB**
STX1A**
SNAP91
CPLX2*
CPLX1**
-2 0 2
z-score normalized Log2 intensity
D
FABP5
FABP5/ Tau 13
E
FH
FH/ Tau 13
F
SLC1A2 (EAAT2)
SLC1A2/ Tau 13
PBS-soluble
Sarkosyl-soluble
AD PSP DLB
B
FABP5-Tau13/ DAPI
FH-Tau13/ DAPI
SCL1A2-Tau13/ DAPI
AD 1
AD 2
AD 3
PSP 1
PSP 2
PSP 3
DLB 1
DLB 2
DLB 3
50 µm
25 µm
C
Secondary antibodies/ DAPI
AD
PSP
DLB
G
FABP5/ Tau 13/ composite
H
FH/ Tau 13/ composite
I
SLC1A2/ Tau 13/ composite
J
Secondary antibodies/ composite
PBS-soluble
Sarkosyl-soluble

Figure EV2. Disease-specifi c tau inteact with SNARE complex and biochemical validation of selected interactors.

Related to Fig. 2. (**A**) SNARE complex interacts with disease-relevant misfolded tau. Heatmap of proteins involved in the SNARE complex. **, Class A interactor ($q < 0.01$); *, Class B interactor ($q < 0.05$) (ANOVA with permutation-based FDR <5%). Interactome analysis identified 13 SNARE complex proteins, with 9 showing disease-specific interaction patterns. STXBP1 and YKT6 were enriched with DLB-derived tau, while SYT1 was depleted from DLB tau. Several SNARE proteins, including STX1A, STX1B, and NAPB, significantly interacted with AD tau, but no significant SNARE enrichment was observed with PSP tau. CPLX1 and CPLX2 were significantly depleted from PSP-derived tau compared to AD- or DLB tau. These results highlight disease-specific tau aggregate interactions with SNARE components involved in synaptic vesicle function. (**B**) PLA images showing Tau13 (total tau) in proximity with FABP5, FH, and SLC1A2 in postmortem cortex from AD (AD1–3), PSP (PSP1–3), and DLB (DLB1–3). Boxed regions are magnified at right. Scale bars: 50 µm (left), 25 µm (right). (**C**) Control immunostaining for secondary antibodies shows no background signal across disease groups (AD, PSP, DLB). (**D–J**) Dual-color dot blot assays of IP materials with T18 antibody (misfolded tau) from PBS- and sarkosyl-soluble fractions probed with Tau13 and antibodies against (**D**, **G**) FABP5, (**E**, **H**) FH, and (**F**, **I**) SLC1A2 in AD, PSP, and DLB samples ($n = 3$ independent cases per group). Blue dots, PBS-soluble; red dots, sarkosyl-soluble. Data presented as mean ± SEM. *$p < 0.05$, **$p < 0.01$, ****$p < 0.0001$ by one-way ANOVA with Tukey test. The adjusted p values are as follows: (**D**) FABP5; PBS-soluble vs sarkosyl-soluble AD tau ($p = 0.0331$), PBS-soluble vs sarkosyl-soluble PSP tau ($p = 0.0032$), PBS-soluble vs sarkosyl-soluble DLB tau ($p = 0.0058$), (**E**) FH; PBS-soluble vs sarkosyl-soluble AD tau ($p < 0.0001$), PBS-soluble vs sarkosyl-soluble PSP tau ($p < 0.0001$), PBS-soluble vs sarkosyl-soluble DLB tau ($p < 0.0001$), (**F**) SLC1A2 (EAAT2); sarkosyl-soluble AD tau vs PSP tau ($p = 0.0101$), sarkosyl-soluble AD tau vs DLB tau ($p = 0.0004$), PBS-soluble vs sarkosyl-soluble PSP tau ($p = 0.0006$), PBS-soluble vs sarkosyl-soluble DLB tau ($p < 0.0001$). (**J**) Dot blot negative control (secondary antibodies only) for PBS- and sarkosyl-soluble IP materials with T18 antibody from AD, PSP, and DLB showing no detectable signal.Source data are available online for this figure.

 © The Author(s)

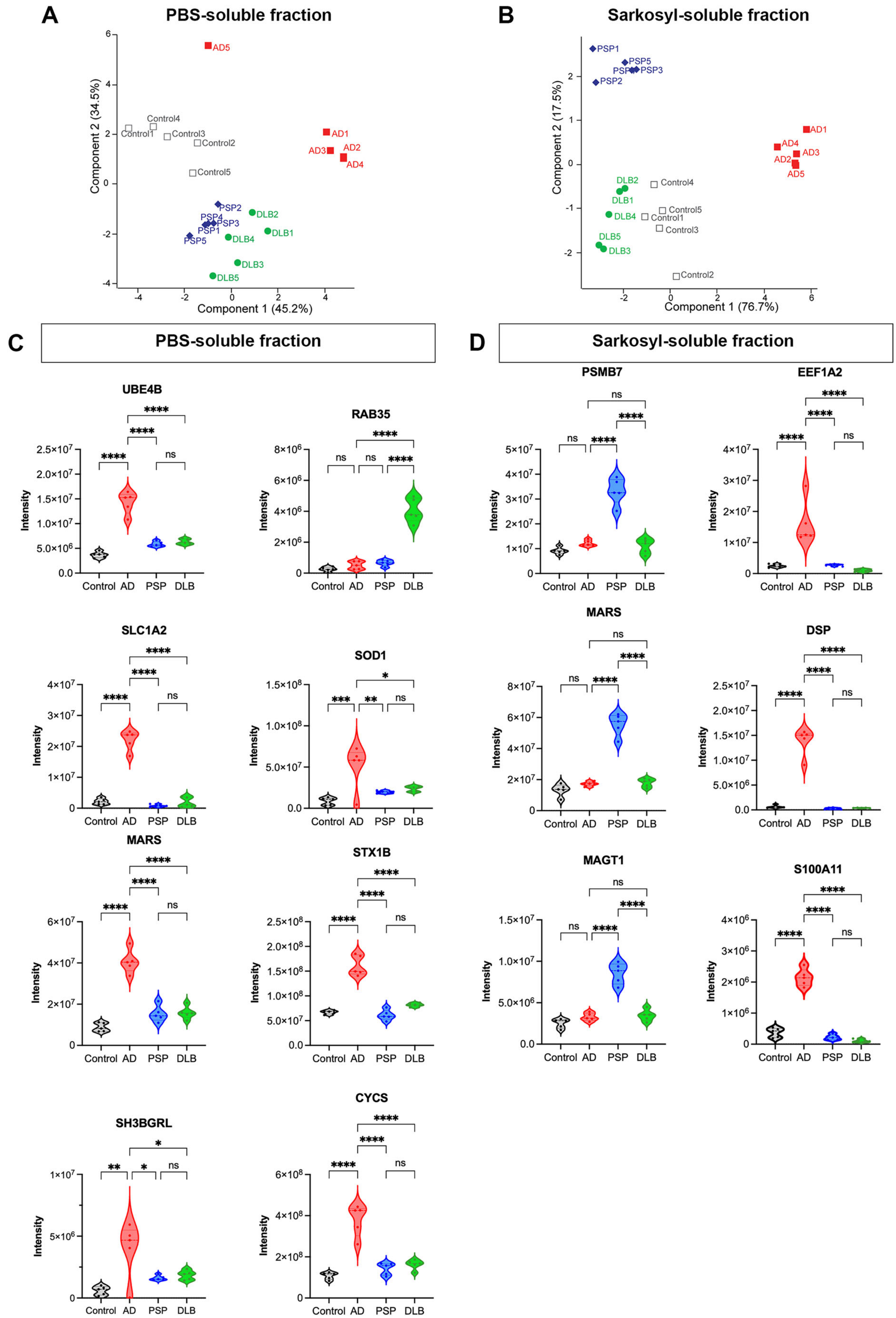
A
PBS-soluble fraction
Component 1 (45.2%)
Component 2 (34.5%)
B
Sarkosyl-soluble fraction
Component 1 (76.7%)
Component 2 (17.5%)
C
PBS-soluble fraction
UBE4B
RAB35
SLC1A2
SOD1
MARS
STX1B
SH3BGRL
CYCS
D
Sarkosyl-soluble fraction
PSMB7
EEF1A2
MARS
DSP
MAGT1
S100A11
Intensity
Control AD PSP DLB

Figure EV3. Related to Fig. 5. Validation of targeted tau interactors in an independent cohort via PRM-MS.

(**A**, **B**) Principal component analysis (PCAs) illustrating the distinct segregation of Con (gray), AD, (red), PSP, (blue), and DLB, (green) cases based on their targeted PRM-MS protein profiles. The distinct clustering demonstrates disease-specific interactomic signatures in both (**A**) PBS-soluble and (**B**) sarkosyl-soluble fractions of the independent validation cohort ($n = 5$ independent cases per group). (**C**, **D**) Targeted quantification of selected significant interactors using PRM-MS in the (**C**) PBS-soluble fraction (UBE4B, RAB35, SLC1A2, SOD1, MARS, STX1B, SH3BGRL, and CYCS) and (**D**) sarkosyl-soluble fraction (PSMB7, EEF1A2, MARS, DSP, MAGT1, and S100A11). Data were presented as violin plots showing the full data distribution, with individual points representing discrete cases across the Con, AD, PSP, and DLB groups ($n = 5$ independent cases per group). *$p < 0.05$, **$p < 0.01$, ***$p < 0.001$, ****$p < 0.0001$, ns not significant, determined by one-way ANOVA followed by Tukey's post hoc test. The adjusted p values are as follows: (**C**) UBE4B; Con vs AD ($p < 0.0001$), AD vs PSP ($p < 0.0001$), AD vs DLB ($p < 0.0001$), SLC1A2; Con vs AD ($p < 0.0001$), AD vs PSP ($p < 0.0001$), AD vs DLB ($p < 0.0001$), MARS; Con vs AD ($p < 0.0001$), AD vs PSP ($p < 0.0001$), AD vs DLB ($p < 0.0001$), SH3BGRL; Con vs AD ($p = 0.0015$), AD vs PSP ($p = 0.0261$), AD vs DLB ($p = 0.0494$), RAB35; AD vs DLB ($p < 0.0001$), PSP vs DLB ($p < 0.0001$), SOD1; Con vs AD ($p = 0.0006$), AD vs PSP ($p = 0.0089$), AD vs DLB ($p = 0.0235$), STX1B; Con vs AD ($p < 0.0001$), AD vs PSP ($p < 0.0001$), AD vs DLB ($p < 0.0001$), CYCS; Con vs AD ($p < 0.0001$), AD vs PSP ($p < 0.0001$), AD vs DLB ($p < 0.0001$), (**D**) PSMB7; AD vs PSP ($p < 0.0001$), PSP vs DLB ($p < 0.0001$), MARS; AD vs PSP ($p < 0.0001$), PSP vs DLB ($p < 0.0001$), MAGT1; AD vs PSP ($p < 0.0001$), PSP vs DLB ($p < 0.0001$), EEF1A2; Con vs AD ($p < 0.0001$), AD vs PSP ($p < 0.0001$), AD vs DLB ($p < 0.0001$), DSP: Con vs AD ($p < 0.0001$), AD vs PSP ($p < 0.0001$), AD vs DLB ($p < 0.0001$), S100A11; Con vs AD ($p < 0.0001$), AD vs PSP ($p < 0.0001$), AD vs DLB ($p < 0.0001$). Source data are available online for this figure.

 © The Author(s)

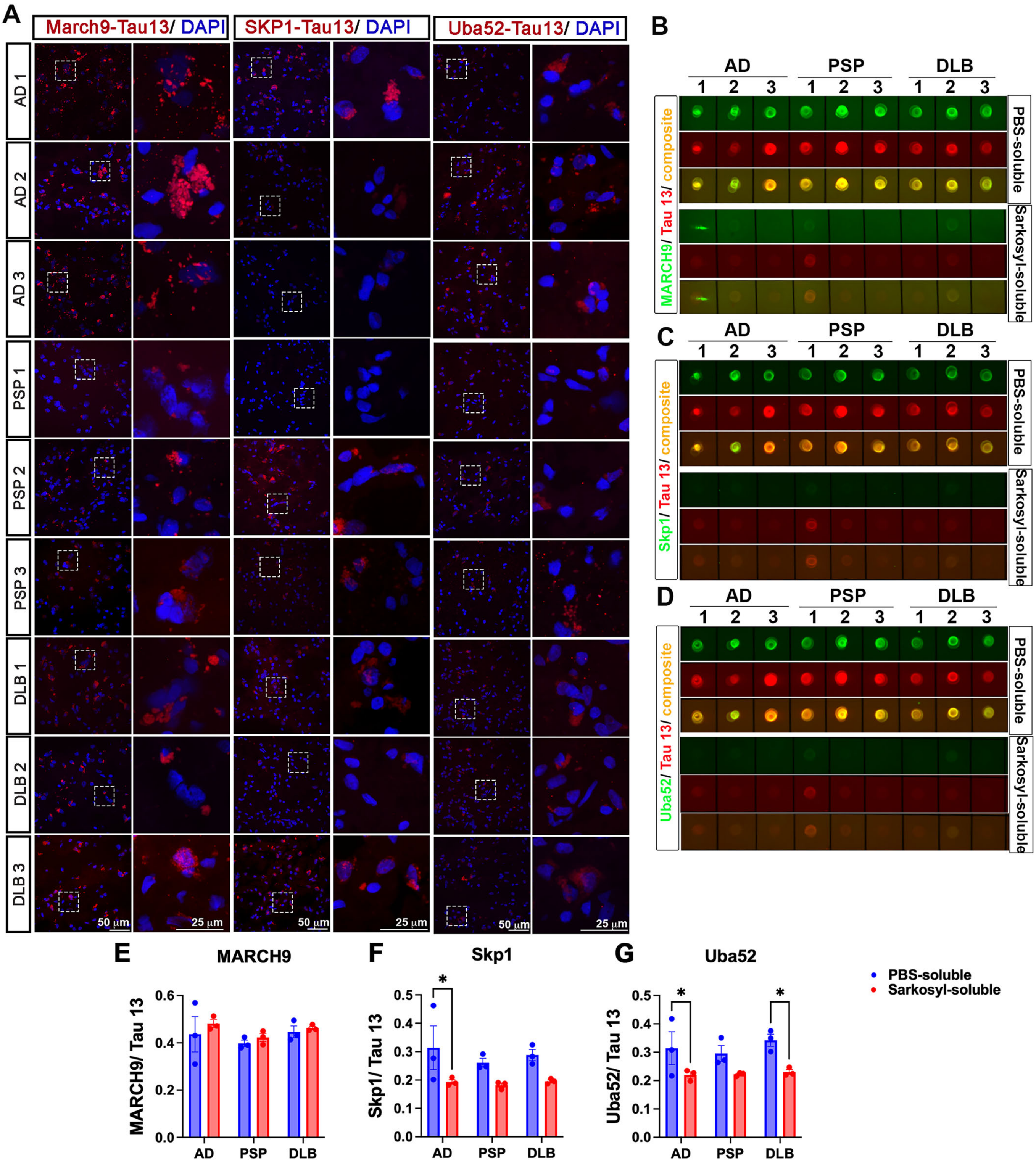

Figure EV4. **Biochemical validation of ubiquitination-related tau interactors.**

Related to Fig. 6. (**A**) Representative PLA images showing colocalization of Tau13 with ubiquitin ligase MARCH9, ubiquitin ligase complex Skp1, and ubiquitin-dependent protein-degradation UBA52 in postmortem brain tissue from AD (AD1–3), PSP (PSP1–3), and DLB (DLB1–3) cases. Boxed regions are magnified at right. Scale bars: 50 µm (left panels), 25 µm (zoom, right panels). (**B–D**) Dot blot analysis of tau immunoprecipitated from PBS- and sarkosyl-soluble brain fractions using Tau13 in combination with antibodies against (**B**) MARCH9, (**C**) Skp1, or (**D**) UBA52. Tau was immunoprecipitated using the T18 antibody from AD, PSP, and DLB PBS-and sarkosyl-soluble fractions ($n = 3$ per disease group). (**E–G**) Quantification of dot-blot signals expressed as protein interactor/Tau13 intensity ratios for (**E**) MARCH9, (**F**) SKP1, and (**G**) UBA52 across AD, PSP, and DLB in PBS (blue) and Sarkosyl (red) fractions. Data were mean ± SEM ($n = 3$ per disease); statistics by two-way ANOVA with Tukey's post hoc test (*$p < 0.05$, **$p < 0.01$). The adjusted p values are as follows: (**F**) SKP1; PBS-soluble vs sarkosyl-soluble AD tau ($p = 0.0265$), (**G**) Uba52; PBS-soluble vs sarkosyl-soluble AD tau ($p = 0.0368$), PBS-soluble vs sarkosyl-soluble DLB tau ($p = 0.0165$). Source data are available online for this figure.

© The Author(s)

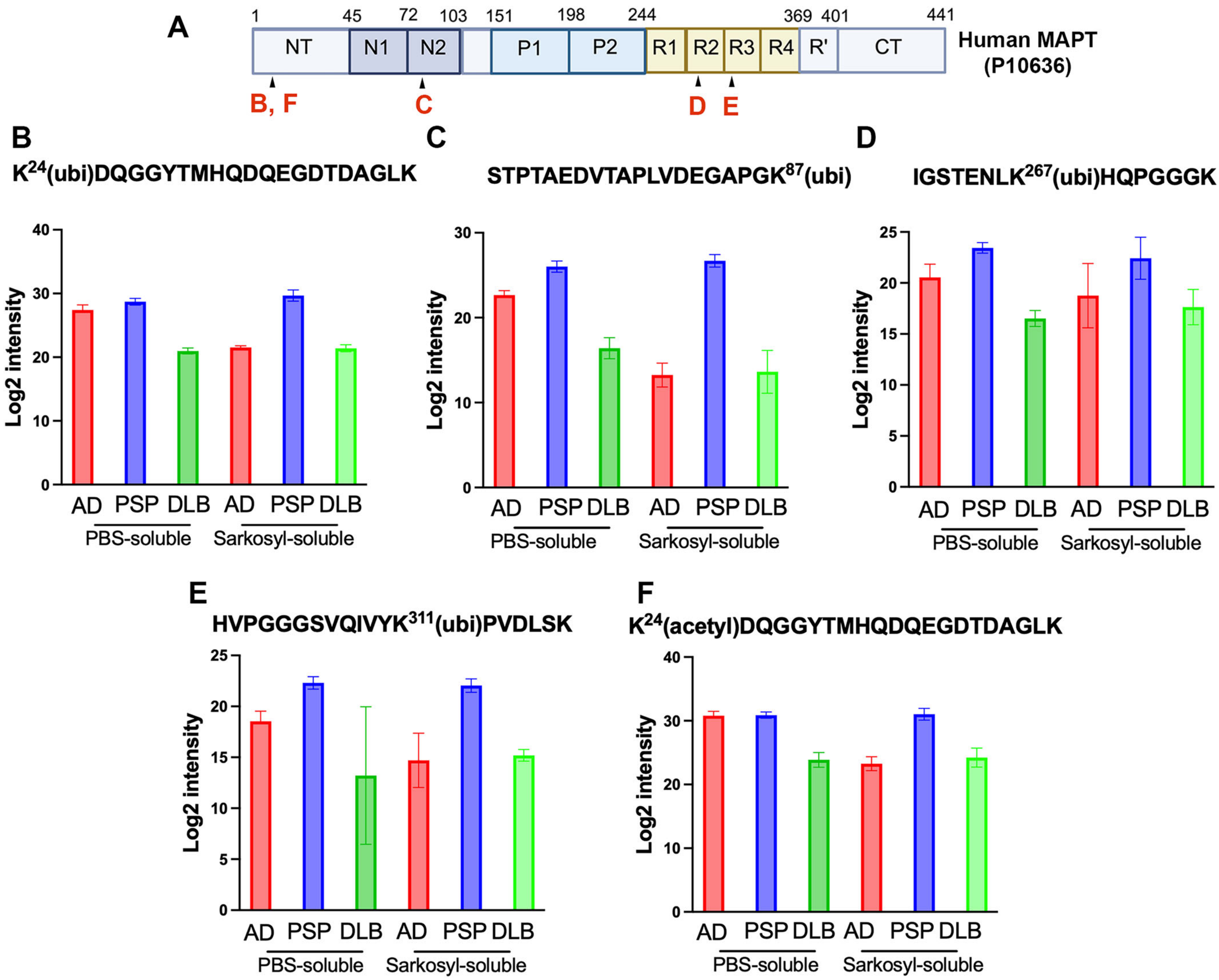

Figure EV5. Related to Figs. 6 and 7. PRM-MS validation of tau ubiquitination and acetylation in the tau interactome.

(**A**) Domain map of human MAPT (UniProt P10636) with red letters marking the locations of the quantified peptides. (**B–E**) Targeted PRM-MS quantification of four ubiquitinated tau peptides in T18 immunoprecipitates from PBS-soluble and Sarkosyl-soluble fractions of AD, PSP, and DLB brains (*n* = 3 per disease). Sequences shown on each panel with modified lysines marked "[ubi]". Bars are $\log_2$ PRM intensities (mean ± SEM). (**F**) Targeted PRM-MS quantification of an acetylated tau peptide (K^{24}[ac] DQGGYTMHQDQEGDTDAGLK) across the same samples and fractions. Bars are $\log_2$ PRM intensities (mean ± SEM).

 © The Author(s)
