# Supplementary material for: Disease-specific tau polymorphs are associated with unique protein networks across proteinopathies
Source: EMBO J. 2026 Aug 18;45(19):6946–77. doi: 10.1038/s44318-026-00897-8 (PMC13631283; doi:10.1038/s44318-026-00897-8)
Supplement: Supplementary file 2 [file 44318_2026_897_MOESM1_ESM.pdf]

THE EMBO JOURNAL

Disease-specific tau polymorphs are associated with unique protein networks across proteinopathies

Nicha Puangmalai, Nemil Bhatt, Nopparat Suthprasertporn, Ernesto Miranda-Morales, Nikita Shchankin, Madison Samples, Ahmet Balci, Jia Yi Liew, Mauro Montalbano, Yingxin Zhao, and Rakez Kayed

Corresponding author(s): Rakez Kayed (rakayed@utmb.edu) , Yingxin Zhao (yizhao@utmb.edu)

Review Timeline:

Submission Date:	8th Sep 25
Editorial Decision:	5th Dec 25
Revision Received:	23rd Jun 26
Editorial Decision:	21st Jul 26
Revision Received:	30th Jul 26
Accepted:	3rd Aug 26

Editor: Hartmut Vodermaier

Transaction Report:

(Note: With the exception of the correction of typographical or spelling errors that could be a source of ambiguity, letters and reports are not edited. Depending on transfer agreements, referee reports obtained elsewhere may or may not be included in this compilation. Referee reports are anonymous unless the Referee chooses to sign their reports.)

Dear Dr. Kayed,

Thank you for submitting your manuscript on distinct interactomes of disease-specific tau aggregate conformations. I am very sorry for the undue delay associated with its evaluation, due to limited availability of referees with the required combination of expertise at this time. We have now finally received reports from three experts in neurodegenerative protein dynamics, neuroproteomics, and tau pathophysiology, copied below for your information. As you will see, all referees appreciate the importance of the question and the potential value of the generated datasets. However, they also retain a number of substantive reservations that would appear to preclude publication of the study in the present form.

While we feel that criticisms about missing causality, functional follow-up, or therapeutic implications could be addressed with more careful phrasing and would not rule out publication of the article as part of our Resource section, we note that various technical issues are more pertinent. This includes concerns regarding sample size, statistical power and reproducibility; incomplete control conditions and inappropriate validation strategies; as well as presentational and data accessibility issues. Since it is not clear whether these problems could be satisfactorily addressed during a regular round of revision, I would at this point invite you to assemble a tentative point-by-point response and revision proposal, and send this back to me by early next week. Based on such a revision plan, which I may in parts share/discuss with some of the referees, we could then get a better understanding of whether a revision for The EMBO Journal would seem realistic, or whether a toned-down version would be a better candidate for rapid publication in one of our sister journals (such as EMBO Reports or Life Science Alliance).

Looking forward to hearing from you,

Best regards,

Hartmut

Hartmut Vodermaier, PhD
Senior Editor, The EMBO Journal
h.vodermaier@embojournal.org

Referee #1 (Report for Author)

General summary and opinion

This manuscript addresses an important question in the field of tauopathies, as it asks whether distinct conformations of aggregated tau proteins are associated with disease-specific interaction networks. The authors combine immunoprecipitation and mass spectrometry from human brain samples of AD, PSP and DLB with targeted validations to identify disease-dependent tau interactomes and post-translational modifications. The topic is timely and relevant, as defining interactome differences across tauopathies could provide insight into disease mechanisms and potentially guide therapeutic strategies.

However, the conclusions drawn in the manuscript tend to overstate the strength of the evidence. While the data show that tau interacts with distinct sets of proteins in different tauopathies, the claims that conformationally distinct tau strains dictate these differences and that therapeutic targets were identified for precision medicine are not fully supported.

Major concerns

1) The conclusion that conformationally distinct tau strains dictate disease-specific protein interaction networks is not directly demonstrated. The study establishes correlations between disease state, tau conformation (which are inferred), and interactome composition, but does not experimentally show that changing tau conformation alters its interactome. Without such causal evidence, the interpretation should be moderated.

2) The sample size (n=3 per disease group) and restriction to a single brain region (frontal cortex) raise concerns about generalizability. Moreover, the use of a single antibody (T18) for tau capture could bias the recovered interactomes. These limitations should be explicitly acknowledged and, if possible addressed, for example by validating in additional samples, regions, or with alternative capture strategies.

3) The machine-learning analysis shows an essentially perfect separation of disease groups using a small set of interactors. However, given the limited sample size, there is a question of overfitting. The conclusions from this analysis should be moderated, and additional validation (for example with an independent cohort) would be required to support the claim of robust disease classification.

4) The study nominates novel interactors of tau, but there is no functional evidence that these proteins are viable therapeutic targets. The current data support their role as candidate interactors but not as validated targets for intervention. The language should be revised to avoid overstatement and to clearly distinguish between discovery of candidate interactors and identification of therapeutic targets.

Minor concerns

1) The criteria for selecting proteins for validation should be described more explicitly.
2) Quantitative details of reproducibility between replicates and across disease groups should be presented more clearly.
3) Some figure legends should be clearer, particularly regarding which interactions are directly confirmed by PLA or dot blot.
4) Statistical details should be consistently reported in the figure legends.

Additional suggestions (non-essential)

As touched upon above, the following point may be considered:
1) Including additional cases or brain regions, if available, would strengthen the robustness of the interactome differences reported.
2) Exploring alternative capture antibodies (targeting distinct tau epitopes) could help rule out potential epitope-driven biases in the interactomes.
3) Functional experiments (perturbing candidate interactors in cellular or animal models) would considerably strengthen the therapeutic relevance of the findings. The authors should at least acknowledge these further studies.

Referee #2 (Report for Author)

Puangmalai and colleagues present a thorough and thoughtfully designed comparative proteomic analysis investigating how distinct conformations of tau aggregates drive disease-specific molecular signatures across Alzheimer's disease (AD), progressive supranuclear palsy (PSP), and dementia with Lewy bodies (DLB). Through conformation-specific immunoprecipitation of misfolded tau (T18 antibody) from PBS- and sarkosyl-soluble brain fractions, followed by label-free LC-MS/MS, the authors identify nearly 500

high-confidence tau interactors with a high disease specificity and no common interactors overlapping across all three tauopathies. Selected candidates are validated via Western blotting and proximity ligation assays (PLA). Functional enrichment and network analyses, including machine learning-based classification, reveal clearly distinct functional landscapes among the diseases.

The study provides strong evidence that tau polymorphs adopt distinct conformations that define unique protein interaction networks, thereby contributing to disease-specific cellular dysfunctions. These findings meaningfully advance our understanding of tauopathy heterogeneity and have the potential to inform future biomarker development and precision therapeutic approaches in neurodegeneration.

Major Points

1. Statistical Power
The use of ANOVA and FDR-controlled analyses is appropriate; however, the relatively small number of biological replicates (three individuals per disease, with two technical replicates each) limits statistical robustness. This constraint should be explicitly acknowledged and discussed in the manuscript.

2. Specificity and Background Controls
Incorporating additional controls would further strengthen the conclusions:
◦ Healthy controls: Although healthy controls should not show marked tau aggregates in the sakroyl-soluble fractions, a comparison with age-matched non-diseased brains (for both soluble and sarkosyl-soluble fractions) would nevertheless help assess the disease specificity of identified interactors.
◦ Matrix/IgG control: Inclusion of a matrix-only or IgG control would help discriminate specific from nonspecific binding events. Without such controls, differences in aggregate composition could influence the apparent interactomes by introducing proteins that bind nonspecifically to the matrix. Although such information can still be biologically informative, it complicates interpretation of specific protein-tau associations and challenges the designation of these proteins as bona fide "tau-interacting."

Minor Points
(a) The number of biological and technical replicates should be clearly stated in both the main text and figure legends, in accordance with journal guidelines.

(b) The release date and version of the UniProt/SwissProt database used for protein

identification should be reported.

(c) While the data-sharing statement regarding RAW data is understandable given patient privacy concerns, providing the complete MaxQuant output tables would enhance transparency as it facilitates to reproduce the data analysis.

Overall Assessment
This is a high-quality and carefully executed study offering valuable mechanistic insight into disease-specific tau interactomes. The results are well presented and interpreted. With clarification of statistical limitations and inclusion or discussion of appropriate specificity controls, the manuscript would be well suited for publication in The EMBO Journal.

Referee #3 (Report for Author)

The manuscript by Puangmalai et al. investigates interactome differences for misfolded Tau between tauopathies. The authors provide a large amount of very interesting Mass Spec data, that informs about differences of oligomeric Tau, recognized by T18 antibody, interactions in AD, PSP, and DLB. Furthermore, they show that different Tau fragments are more or less abundant in these diseases, and that they are differentially posttranslational modified (Ub, Ac, Ph). Unfortunately, these two parts appear quite disconnected from each other. Beyond the data provided, the authors do not provide mechanistic data on why the interactomes are different, and how they differentially shape Tau function and neuronal pathways. Therefore, although the authors use a logical experimental approach to assess the tau interactome, several points would need clarification and improvement. Unfortunately, I cannot support publication in EMBO Journal in the current state of the manuscript.
To improve the manuscript and add causal links between interactomes and Tau fragments/PTMs, the authors could try to show that different interactors bind to certain Tau fragments and are dependent on certain PTMs, and that pathways, in which the identified interactors are involved, are differentially dysregulated in the different diseases. It would. Make sense to focus on no more than 1 or 2 pathways then.
Major points:
- I understand that the authors do a comparative analysis of what is pulled does with T18 in different tauopathies. There is no data shown, however, how specific the AB is for Tau and for oligomeric Tau. How do we know that the pull down is not just pulling total Tau interactomes?
- The T18 Tau interactome should be prepared for control brain cases as well.

- I may have missed it, but I could not find a volcano plot or other analysis comparing the input proteomes (without T18 IP) between diseases (PBS/Sarkosyl). Are they similar or different between diseases? - this would influence the T18 interactome.
- A Western Blot showing total Tau in the input and pulldown fractions of the IP fractions would be needed to show that Tau actually was pulled down and eluted from the T18 column.
- PLAs in Fig 2F-I, Fig 3 G+H, Fig 6H-K are not convincing at all. 1) There are no PLA "dots" to see and the images are full of background and image processing artifacts - the imaging resolution is obviously not good enough and the background needs to be quenched. 2) why has the PLA been done with Tau13 (a total human Tau AB) and not with T18? - 3) PLA is not the right way to show the amount of interaction, esp. when having two soluble proteins in the same cell compartment, the crowded cytosol. PLA can show where interactions happen and has a proximity radius of ~20-30 nm, so it will give signal mostly everywhere in the cytosol. The authors should replace the PLA data with classic co-IP and Western Blot to show increased or decreased interactions of Tau with the selected targets. 4) co-staining with neuronal marker would be necessary to show what we are looking at here.
- It is absolute unclear why the authors use the term "Tau polymorph" in the title as well as in the manuscript. Polymorph comes from morphology = appearance, poly = many. The authors use one kind of conformation/assembly specific antibody, T18, to fish out Tau interactors. Means, the differences in interactors they find are disease-specific for one kind of Tau assembly/conformation/morphology type. The nomenclature "Tau polymorphs" is misleading. Th etitle should be changed in this regard as well.
- The Tau fragment analysis incl PTM analysis, is currently disconnected from the rest of the MS. It is not clear, why the authors did these analyses. I guess the aim was to understand which Tau fragments may be relevant for the interactions identified, and, additionally, how PTMs of Tau influence the interactions. However, this at the moment is not being analyzed, and the Tau fragment data - although very interesting and complex - provide no mechanistic insides into how they shape the Tau interactome, or how they may be connected to Tau conformation/assembly detected by the T18 antibody. If the authors could exemplify how the Tau fragment and PTMs identified influence the interaction of Tau with one or two important interactors, this would certainly boost the impact. At the current state, it seems to remain a collection of interesting observations that are too complex to grasp and therefore remain unclear.

Minor points:
- The authors identify different Tau peptide composition in AD, PSP, LBD cortex. This is very valuable information; however, the authors do not describe or discuss the meaning of these data. More discussion of MS data for Tau fragments in AD/PSP/DLB (Fig. 1 and S1).

Also, Data/Figure for Tau fragment analysis shown in Fig 1 should be shifted in front of ubiquitination/PTM analysis of Tau fragments in Fig 6.

- Please provide the protein names of the most deregulated proteins in Fig 1J-P. This would be useful for all enriched /depleted interactors for all three diseases, e.g., as an additional Supplementary Table.
- Please clarify protocol for Sarkosyl soluble fraction in Methods; clearly state which fraction was taken for IP
- There are very few interactors of Tau pulled down with T18 in the sarkosyl-soluble IP. Obviously, the Sarkosyl can denature proteins and prohibit interactions. Did the authors verify that T18 is binding Tau in 1% Sarkosyl?
- Double check Ref 66, please
- Fig 1M > the Venn diagram seems to not match the volcano plots in J, K, L. Please double check
- Please list the significant enriched proteins in the Figure, so that readers can easily access. For the string networks, please state clearly what are you showing. Currently it is confusing that the protein numbers of the interactors don't match the number of nodes in the STRING.
- Please make the way, how interactors are shown for the different diseases, more uniform. It is confusing that sometimes are STRING networks and sometimes heatmaps are shown. Also, include all significant interactors in the heat maps.
- Please provide a list (Supp Table and listed in Figure for PSP and DLB) of significant enriched and depleted interactors, so the reader has an easier access to the data/findings.
- Fig 4, sarkosyl soluble fraction interactomes, please show the heatmap of significant enriched/depleted interactors across disease, like for PBS-soluble interactors in Fig. 2.
- Please also show interactors common between diseases in the String networks (e.g., different shading), to make the reader aware of all interactions happening.
- Our lab has not much expertise in machine learning, but is there a way to show significance for the difference of the 10 signature proteins between the disease in Fig 5B and F?
- Fig. 7, PTM analysis of Tau fragments, currently does not add much to the manuscript story and should be moved to supplemental material.

Dear Dr. Vodermaier.

We want to thank you and the reviewers for the comments on our manuscripts.

As you suggested, we will revise the manuscript EMBOJ-2025-122410 to address the criticisms about missing causality, functional follow-up, or therapeutic implications.

Regarding various technical, presentation, and data accessibility issues, we have provided a point-by-point response and revision proposal as follows.

REVIEWER 1.

Major concerns

1. The conclusion that conformationally distinct tau strains dictate disease-specific protein interaction networks is not directly demonstrated. The study establishes correlations between disease state, tau conformation (which are inferred), and interactome composition, but does not experimentally show that changing tau conformation alters its interactome. Without such causal evidence, the interpretation should be moderated.

Response: Thank you for bringing up this point. We will revise the discussion to carefully distinguish between findings, correlative associations, and mechanistic interpretations, clarifying the limitations of our current resource dataset.

2. *The sample size (n=3 per disease group) and restriction to a single brain region (frontal cortex) raise concerns about generalizability. Moreover, the use of a single antibody (T18) for tau capture could bias the recovered interactomes. These limitations should be explicitly acknowledged and, if possible addressed, for example by validating in additional samples, regions, or with alternative capture strategies.*

Response: Thank you for your comment. We have validated some of the significant interactome findings in the same brain tissues using independent methods such as PLA and Western Blot. To demonstrate the generalizability of the findings, we will validate the significant results using a separate cohort (n=5 per group) in addition to the cohort present in the current version of the manuscript.

3. *The machine-learning analysis shows an essentially perfect separation of disease groups using a small set of interactors. However, given the limited sample size, there is a question of overfitting. The conclusions from this analysis should be moderated, and additional validation (for example with an independent cohort) would be required to support the claim of robust disease classification.*

Response: We will validate the finding with a separate cohort (n=5 per group) in addition to the cohort presented in the current of the manuscript and targeted MS analysis.

Minor concerns

1. *Quantitative details of reproducibility between replicates and across disease groups should be presented more clearly.*

Response: We will provide additional data to demonstrate reproducibility between replicates and across disease groups.

2. *Some figure legends should be clearer, particularly regarding which interactions are directly confirmed by PLA or dot blot.*

Response: Thank the reviewer's suggestion. We will make changes as suggested.

3. *Statistical details should be consistently reported in the figure legends.*

Response: Thank the reviewer's suggestion. We will make changes as suggested.

REVIEWER 2.

Major Points

7. The use of ANOVA and FDR-controlled analyses is appropriate; however, the relatively small number of biological replicates (three individuals per disease, with two technical replicates each) limits statistical robustness. This constraint should be explicitly acknowledged and discussed in the manuscript.

Response: Thank the reviewer's comment. We will explicitly acknowledge and discuss this limitation in the manuscript. Additionally, we will validate the significant findings using an additional separate cohort (n=5 per group).

8. *Although healthy controls should not show marked tau aggregates in the sakroyl-soluble fractions, a comparison with age-matched non-diseased brains (for both soluble and sarkosyl-soluble fractions) would nevertheless help assess the disease specificity of identified interactors.*

Response: Thank the reviewer's comment. We will include healthy controls in our validation study.

9. *Inclusion of a matrix-only or IgG control would help discriminate specific from nonspecific binding events. Without such controls, differences in aggregate composition could influence the apparent interactomes by introducing proteins that bind nonspecifically to the matrix. Although such information can still be biologically informative, it complicates interpretation of specific protein-tau associations and challenges the designation of these proteins as bona fide "tau-interacting."*

Response: We used a Hawaii plot with a complement negative control, an advanced multi-volcano plot for analyzing large-scale affinity purification–mass spectrometry (AP-MS). This method allows for a global statistical comparison of multiple AP-MS experiments against a single, robust negative control. The complement-negative control strategy used a composite control that was created from the experimental data itself rather than a single dedicated control sample. For the Tau pulldown protein experiment from AD, DLP, and PSP brains, an internal negative control is assembled from the combined pull-downs of all experiments, which better reflects the average background binding across the entire dataset. The Hawaii plot then depicts each AP pull-down relative to this unique, internally generated complement-negative control, thereby accurately identifying genuine protein interactions and eliminating the matrix effect.

Minor Points

10. *The number of biological and technical replicates should be clearly stated in both the main text and figure legends, in accordance with journal guidelines.*

Response: Thank the reviewer's suggestion. We will make changes as suggested.

11. The release date and version of the UniProt/SwissProt database used for protein identification should be reported.

Response: Thank the reviewer's suggestion. We will make changes as suggested.

12. While the data-sharing statement regarding RAW data is understandable given patient privacy concerns, providing the complete MaxQuant output tables would enhance transparency as it facilitates to reproduce the data analysis.

Response: Thank the **reviewer's** suggestion. We will provide a ZIP file containing the MaxQuant output tables as supplemental data.

REVIEWER 3

Major points

13. I understand that the authors do a comparative analysis of what is pulled does with T18 in different tauopathies. There is no data shown, however, how specific the AB is for Tau and for oligomeric Tau. How do we know that the pull down is not just pulling total Tau interactomes?

Response: Thank you for the comment. We have shown the use of T18 to distinguish soluble tau in several of our previous works (PMID: 39809992, 32737202, 40823851, 40681153, 35202653, 39239152, 38135105, 33246331). Moreover, we will provide IHC staining using T18 of AD, PSP, and DLB.

14. *The T18 Tau interactome should be prepared for control brain cases as well.*

Response: Thank the reviewer's comment. We will include healthy controls in our validation study.

15. *I may have missed it, but I could not find a volcano plot or other analysis comparing the input proteomes (without T18 IP) between diseases (PBS/Sarkosyl). Are they similar or different between diseases? - this would influence the T18 interactome.*

Response: We conducted the proteome of the brain frontal cortex and found that the these results suggested that the significant changes observed in misfolded tau pull-downs were caused by alterations in protein-protein interactions, rather than differential expression levels in the brains of patients with AD, PSP, and DLB (Sup. Fig.1O). We will provide volcano plots of the proteome of the brain frontal cortex.

16. *A Western Blot showing total Tau in the input and pulldown fractions of the IP fractions would be needed to show that Tau actually was pulled down and eluted from the T18 column.*

Response: In our interactome study, we identified tau with high sequence coverage, indicating that the tau protein was successfully pulled down and eluted from the T18 column.

17. *PLAs in Fig 2F-I, Fig 3 G+H, Fig 6H-K are not convincing at all. 1) There are no PLA "dots" to see and the images are full of background and image processing artifacts - the imaging resolution is obviously not good enough and the background needs to be quenched. 2) why has the PLA been done with Tau13 (a total human Tau AB) and not with T18? - 3) PLA is not the right way to show the amount of interaction, esp. when having two soluble proteins in the same cell compartment, the crowded cytosol. PLA can show where interactions happen and has a proximity radius of ~20-30 nm, so it will give signal mostly everywhere in the cytosol. The authors should replace the PLA data with classic co-IP and Western Blot to show increased or decreased interactions of Tau with the selected targets. 4) co-staining with neuronal marker would be necessary to show what we are looking at here.*

Response: Thank you for these constructive comments regarding the PLA results.

1) We and others have used the PLA method to confirm the protein-protein interaction in human and mouse brain tissues, which is not always shown as PLA dots, such as the results in Jensen et al., 2024 (PMID 39613827), due to the high abundance of the target proteins and their protein-protein interaction. Regarding this matter, we quantified PLA area (puncta per field) as an appropriate approach rather than dot counts.
2) For the quality control of PLA results, we have included secondary antibody-only staining (Sup. Fig. 2C) to determine the non-specific or background staining. All the images were taken from high-resolution confocal microscopy; however, we will ensure that we adjust the image resolution up to 600 dpi for submission.
3) PLA analysis was performed to validate the MS results of the total tau interactome using Tau13, disregarding their solubility.
4) Thank you for the reviewer's comment on the co-staining with the neuronal marker (MAP2). We will make the change as suggested.

18. *It is absolute unclear why the authors use the term "Tau polymorph" in the title as well as in the manuscript. Polymorph comes from morphology = appearance, poly = many. The authors use one kind of conformation/assembly specific antibody, T18, to fish out Tau interactors. Means, the differences in interactors they find are disease-specific for one kind of Tau assembly/conformation/morphology type. The nomenclature "Tau polymorphs" is misleading. The title should be changed in this regard as well.*

Response: Thank you for the suggestion, however, the authors feel the word polymorph is appropriately used as soluble tau from AD, PSP and DLB have been shown to have different conformations, morphologies, seeding activities, affect neuronal functions, and modulate gene expression differently in excitatory neurons (PMID 39809992). Moreover, fibrillar tau from several neurodegenerative diseases show different conformations at an atomic level (PMID: 34588692). Additionally, analysis of protease cleavage is a widely used technique for distinguishing different conformations for amyloid proteins (PMID: 9771749, 39809992, 40681153, 39145409,

38503425, 38503425, 32350746). Our Tau peptide compositions show that T18-IP AD, PSP, and DLB derived tau exhibit distinct conformations.

19. *The authors identify different Tau peptide composition in AD, PSP, LBD cortex. This is very valuable information; however, the authors do not describe or discuss the meaning of these data. More discussion of MS data for Tau fragments in AD/PSP/DLB (Fig. 1 and S1). Also, Data/Figure for Tau fragment analysis shown in Fig 1 should be shifted in front of ubiquitination/PTM analysis of Tau fragments in Fig 6.*

Response: Thank the reviewer's suggestion. As mentioned above, the use of protease cleavage is critical technique for distinguishing distinct confirmations (PMID: 9771749, 39809992, 40681153, 39145409, 38503425, 38503425, 32350746). We will make changes as suggested.

20. Please provide the protein names of the most deregulated proteins in Fig 1J-P. This would be useful for all enriched /depleted interactors for all three diseases, e.g., as an additional Supplementary Table.

Response: Thank the reviewer's suggestion. We will make changes as suggested.

21. Please clarify protocol for Sarkosyl soluble fraction in Methods; clearly state which fraction was taken for IP

Response: Thank the reviewer's suggestion. We will make changes as suggested in Methods.

22. There are very few interactors of Tau pulled down with T18 in the sarkosyl-soluble IP. Obviously, the Sarkosyl can denature proteins and prohibit interactions. Did the authors verify that T18 is binding Tau in 1% Sarkosyl?

Response: Thank you. Results in Sup. Fig.1A demonstrated the total tau intensity of IP with T18 antibody in the sarkosyl-soluble fraction.

23. Double check Ref 66, please

Response: Thank you. We will correct the reference.

24. Fig 1M > the Venn diagram seems to not match the volcano plots in J, K, L. Please double check

Response: Thank the reviewer's suggestion. We will make changes as suggested.

25. Please list the significant enriched proteins in the Figure, so that readers can easily access. For the string networks, please state clearly what are you showing.

Response: Thank the reviewer's suggestion. We will make changes as suggested.

26. *Please make the way, how interactors are shown for the different diseases, more uniform. It is confusing that sometimes are STRING networks and sometimes heatmaps are shown. Also, include all significant interactors in the heat maps.*

Response: Thank the reviewer's suggestion. We will make changes as suggested.

27. Please provide a list (Supp Table and listed in Figure for PSP and DLB) of significant enriched and depleted interactors, so the reader has an easier access to the data/findings.

Response: These significantly enriched and depleted interactors have been provided as supplemental tables.

28. Fig 4, sarkosyl soluble fraction interactomes, please show the heatmap of significant enriched/depleted interactors across disease, like for PBS-soluble interactors in Fig. 2.

Response: Thank the reviewer's suggestion. We will make changes as suggested.

29. Please also show interactors common between diseases in the String networks (e.g., different shading), to make the reader aware of all interactions happening.

Response: Thank the reviewer's suggestion. We will make changes as suggested.

30. Our lab has not much expertise in machine learning, but is there a way to show significance for the difference of the 10 signature proteins between the disease in Fig 5B and F?

Response: The ranking of the significance of the 10 signature proteins between the disease in Fig. 5B and F are listed in Table 2.

1st Editorial Decision 5th Dec 2025

Prof. Rakez Kayed
The University of Texas Medical Branch
Neurology
301 University Blvd
Galveston, Texas 77555-1045

5th Dec 2025

Re: EMBOJ-2025-122410
Disease-specific tau polymorphs define unique protein interaction networks across proteinopathies

Dear Dr. Kayed,

Thank you for your letter outlining how you might address the referee comments on your recent EMBO Journal submission. I have now had a chance to go through your revision plan, and since the majority of your responses appear reasonable and promising, I would be happy to formally invite you to revise the study for potential publication as a Resource Article in The EMBO Journal. I realize that particularly the extension towards sampling a separate cohort should be able to address several shared concerns of all three referees. My main remaining concern is the response to point 17, in which referee 3 has raised issues with the interaction validation based on PLA: here, I feel that in addition to your clarifications and already proposed additions, it would be still be important to complement the PLA data with biochemical interaction studies (co-IP/Western blot), especially given that antibodies against the tested interactors appear to be available. When revising the study as a Resource Article, please make sure to follow our guidelines for deposition of proteomics datasets (explained at https://link.springer.com/partners/embo-press/editorial-policies/data-deposition and https://link.springer.com/partners/embo-press/editorial-policies#Materials%20and%20data%20sharing). Furthermore, please carefully go through the reference list, double-checking each citation for correctness and making sure to closely adhere to EMBO Journal reference formatting as explained at https://link.springer.com/journal/44318/submission-guidelines#cms-Reference-guidelines . Finally, please shorten the abstract to fit within our journal format of max. 175 words, but at the same time provide a somewhat more extended and informative introduction, with sufficient background to make the article accessible also to non-expert readers.

I should add that it is our policy to allow only a single round of (major) revision, making it important to comprehensively and diligently answer to all points raised by the referees at the time of resubmission. Should this require more than our default 3-months revision period, please let us know and we would be happy to extend the deadline. As always, competing manuscript published elsewhere during the course of this revision will not affect our eventual decision on your study. Finally, please note all detailed further information and guidelines on how to prepare a revision below (and in our online Guide to Authors: https://link.springer.com/journal/44318/submission-guidelines#cms-Revised-submissions) - closely adhering to them shall greatly facilitate the editorial process at the time of resubmission.

Thank you again for the opportunity to consider this work, and I look forward to receiving your revision in due time.

Best regards,

Hartmut Vodermaier

Hartmut Vodermaier, PhD
Senior Editor, The EMBO Journal
h.vodermaier@embojournal.org

*** PLEASE NOTE: All revised manuscript are subject to initial checks for completeness and adherence to our formatting guidelines. Revisions may be returned to the authors and delayed in their editorial re-evaluation if they fail to comply to the following requirements. As a first step please read our guidelines for revised submissions: https://link.springer.com/journal/44318/submission-guidelines#cms-Revised-submissions

1) Every manuscript requires a Data Availability section (even if only stating that no deposited datasets are included). Primary datasets or computer code produced in the current study have to be deposited in appropriate public repositories prior to resubmission, and reviewer access details provided in case that public access is not yet allowed.

2) Each figure legend must specify
- size of the scale bars that are mandatory for all micrograph panels

- the statistical test used to generate error bars and P-values
- the type error bars (e.g., S.E.M., S.D.)
- the number (n) and nature (biological or technical replicate) of independent experiments underlying each data point
- Figures may not include error bars for experiments with n<3; scatter plots showing individual data points should be used instead.

3) Revised manuscript text (including main tables, and figure legends for main and EV figures) has to be submitted as editable text file (e.g., .docx format). We encourage highlighting of changes (e.g., via text color) for the referees' reference.

4) Each main and each Expanded View (EV) figure should be uploaded as individual production-quality files (preferably in .eps, .tif, .jpg formats). For suggestions on figure preparation/layout, please refer to our Figure Preparation Guidelines: https://media.springernature.com/original/springer-cms/rest/v1/content/27825798/data/v1

5) Point-by-point response letters should include the original referee comments in full together with your detailed responses to them (and to specific editor requests if applicable), and also be uploaded as editable (e.g., .docx) text files.

6) Please complete our Author Checklist, and make sure that information entered into the checklist is also reflected in the manuscript; the checklist will be available to readers as part of the Review Process File.

7) All authors listed as (co-)corresponding need to deposit, in their respective author profiles in our submission system, a unique ORCiD identifier linked to their name. Please see our Guide to Authors for detailed instructions.

8) Please note that supplementary information at EMBO Press has been superseded by the 'Expanded View' for inclusion of additional figures, tables, movies or datasets; with up to five EV Figures being typeset and directly accessible in the HTML version of the article.

9) To facilitate reproducibilty and cross-laboratory adoption of methodologies, please structure the Materials & Methods section as outlined in our guide to authors, including a completed Reagents and Tools Table.

10) Digital image enhancement is acceptable practice, as long as it accurately represents the original data and conforms to community standards. If a figure has been subjected to significant electronic manipulation, this must be clearly noted in the figure legend and/or the 'Materials and Methods' section. The editors reserve the right to request original versions of figures and the original images that were used to assemble the figure. Finally, we generally encourage uploading of numerical as well as gel/blot image source data.

At EMBO Press we ask authors to provide source data for the main manuscript figures. You will receive a separate email with instructions for providing source data with your revised manuscript, including how to upload and organize the files.

In the interest of ensuring the conceptual advance provided by the work, we recommend submitting a revision within 3 months (5th Mar 2026). Please discuss the revision progress ahead of this time with the editor if you require more time to complete the revisions.

Dear Dr. Vodermaier.

We want to thank you and the reviewers for the comments on our manuscripts.

As you suggested, we have revised the manuscript to address the criticisms about missing causality, functional follow-up, or therapeutic implications.

Regarding various technical, presentation, and data accessibility issues, we have provided a point-by-point response and revision proposal as follows.

Editor's comments

Thank you for your letter outlining how you might address the referee comments on your recent EMBO Journal submission. I have now had a chance to go through your revision plan, and since the majority of your responses appear reasonable and promising, I would be happy to formally invite you to revise the study for potential publication as a Resource Article in The EMBO Journal. I realize that particularly the extension towards sampling a separate cohort should be able to address several shared concerns of all three referees. My main remaining concern is the response to point 17, in which referee 3 has raised issues with the interaction validation based on PLA: here, I feel that in addition to your clarifications and already proposed additions, it would be still be important to complement the PLA data with biochemical interaction studies (co-IP/Western blot), especially given that antibodies against the tested interactors appear to be available.

Response: Thank you very much for your encouragement and insightful suggestion. We have complemented the PLA data with biochemical interaction studies (co-IP/Western blot) in the revised **Fig. 2**, **3**, and **6**. Additionally, we have validated the significant results in a separate cohort (n=5 per group) in addition to the cohort present in the manuscript, using parallel reaction monitoring-mass spectrometry (PRM-MS) analysis, as shown in **Fig. EV3** and **Appendix Table S6.**

Referee #1:

General summary and opinion

This manuscript addresses an important question in the field of tauopathies, as it asks whether distinct conformations of aggregated tau proteins are associated with disease-specific interaction networks. The authors combine immunoprecipitation and mass spectrometry from human brain samples of AD, PSP and DLB with targeted validations to identify disease-dependent tau interactomes and post-translational modifications. The topic is timely and relevant, as defining interactome differences across tauopathies could provide insight into disease mechanisms and potentially guide therapeutic strategies. However, the conclusions drawn in the manuscript tend to overstate the strength of the evidence. While the data show that tau interacts with distinct sets of proteins in different tauopathies, the claims that conformationally distinct tau strains dictate these differences and that therapeutic targets were identified for precision medicine are not fully supported.

Major concerns

1. The conclusion that conformationally distinct tau strains dictate disease-specific protein interaction networks is not directly demonstrated. The study establishes correlations between disease state, tau conformation (which are inferred), and interactome composition, but does not experimentally show that changing tau conformation alters its interactome. Without such causal evidence, the interpretation should be moderated.

Response: Thank you for bringing up this point. We have revised the discussion to carefully distinguish between findings, correlative associations, and mechanistic interpretations, clarifying the limitations of our current resource dataset (lines 452-472).

2. The sample size (n=3 per disease group) and restriction to a single brain region (frontal cortex) raise concerns about generalizability. Moreover, the use of a single antibody (T18) for tau capture could bias the recovered interactomes. These limitations should be explicitly acknowledged and, if possible addressed, for example by validating in additional samples, regions, or with alternative capture strategies.

Response: Thank you for your comment. We have validated some of the significant interactome findings in the same brain tissues using independent methods such as PLA and Western Blot, as shown in revised **Fig. 2, 3,** and **6**. To demonstrate the generalizability of the findings, we have validated the significant results in a separate cohort (n=5 per group) in addition to the cohort present in the manuscript, using parallel reaction monitoring-mass spectrometry (PRM-MS) analysis, as shown in **Fig. EV3 and Appendix Table S6**. In addition, the frontal cortex was selected because it consistently involved across AD, PSP, and DLB, permitting a direct cross-disease comparison. Moreover, the use of a single antibody T18 can therefore bias the recovered interactome, however, we have shown the use of T18 to distinguish soluble tau in several of our previous works (PMID: 39809992, 32737202, 40823851, 40681153, 35202653, 39239152, 38135105, 33246331, 41610849, 39688618). Reassuringly, key interactions were independently confirmed in tissue with an orthogonal total-tau antibody (Tau13) by proximity ligation assay and by western blot analysis. This has been added in the discussion under the limitation paragraph (lines 452-472).

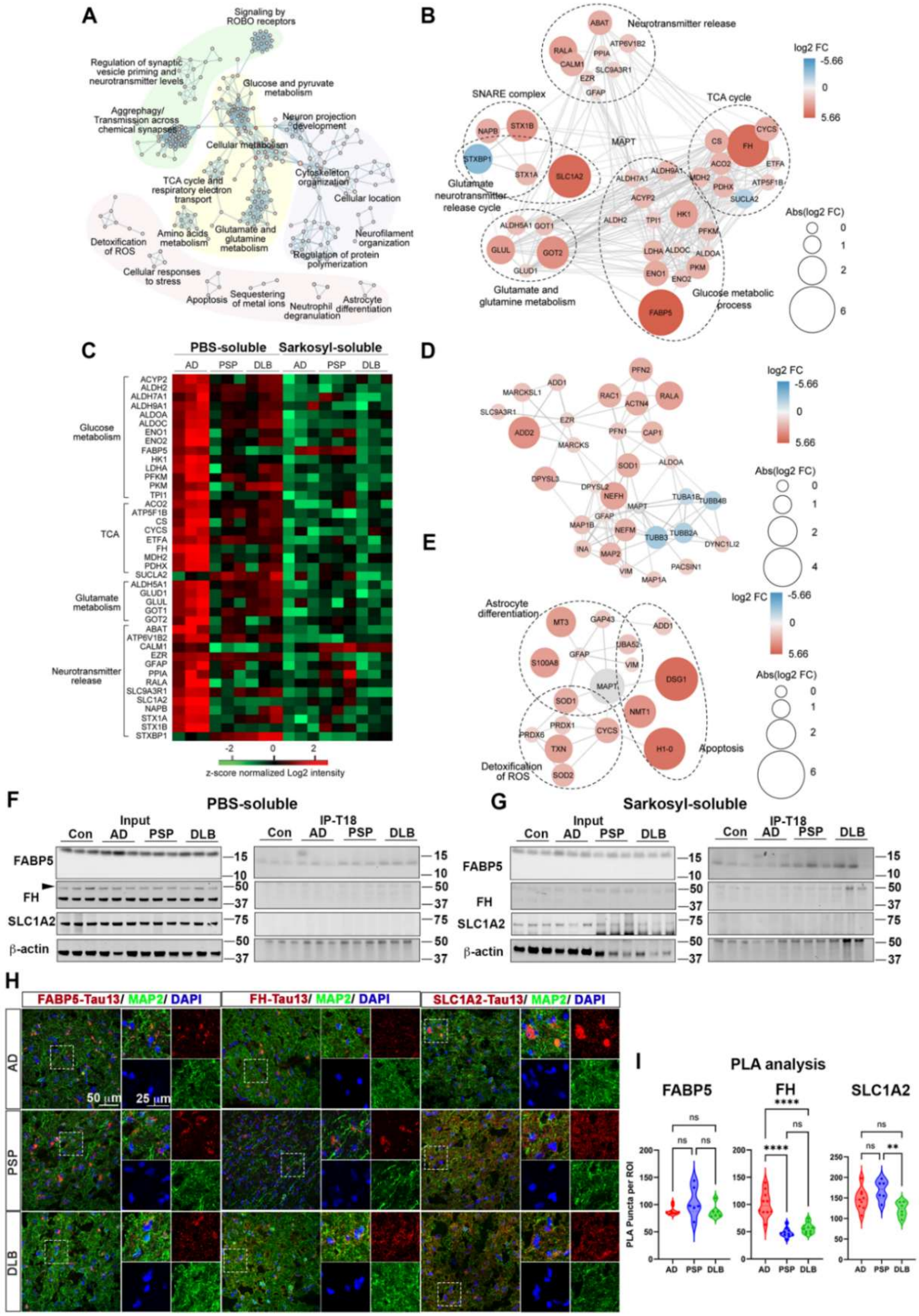

Figure 2. Characterization of PBS- and sarkosyl-soluble AD tau interactome. (A) Functional landscape of the PBS-soluble tau interactome. The network map highlights enriched biological processes among the 169 proteins significantly enriched with, or depleted

from, misfolded tau immunoprecipitated from PBS-soluble AD brain extracts. **(B)** Sub-network analysis of significant AD tau interactors associated with glucose metabolism, the TCA cycle, glutamine and glutamate metabolism, and neurotransmitter release (including the SNARE complex). Node color indicates the log2 fold change (FC) relative to complement controls (red: enriched in AD; blue: depleted in AD), and node size reflects the absolute magnitude.

(**C**) Heatmap displaying the relative abundance of the significant interactors highlighted in (**B**). Data are shown as z-score–normalized log2 LFQ intensities across both PBS- and sarkosyl-soluble fractions from AD, PSP, and DLB (ANOVA with permutation-based FDR < 5%).

(D, E) Sub-networks detailing significant AD tau interactors implicated in **(D)** cytoskeleton organization and **(E)** reactive oxygen species (ROS) detoxification, astrocyte differentiation, and apoptosis. Node color indicates the log2 FC relative to complement controls (red: enriched in AD; blue: depleted in AD), and node size reflects the absolute magnitude.

(F, G) Biochemical validation of selected interactors. Representative WB of inputs and misfolded-tau (T18) immunoprecipitates (IP-T18) from **(F)** PBS-soluble and **(G)** sarkosyl-soluble fractions of Control (Con), AD, PSP, and DLB brain extracts. Blots were probed for FABP5, FH, and SLC1A2. β-actin serves as a loading control.

(H) Validation of *in situ* protein interactions via PLA. Representative confocal micrographs showing PLA signals (red) for FABP5–Tau13, FH–Tau13, and SLC1A2–Tau13 interactions in AD, PSP, and DLB brain sections. Neurons are counterstained with MAP2 (green) and nuclei with DAPI (blue). Scale bars: 50 μm (main panels) and 25 μm (magnified insets).

(I) Quantification of PLA puncta per region of interest (ROI) for the interactions shown in (H). Data are presented as violin plots showing the data distribution (n = 3 independent cases per group). *p < 0.05, p < 0.01, ****p < 0.0001, ns = not significant, determined by one-way ANOVA followed by Tukey's post hoc test.

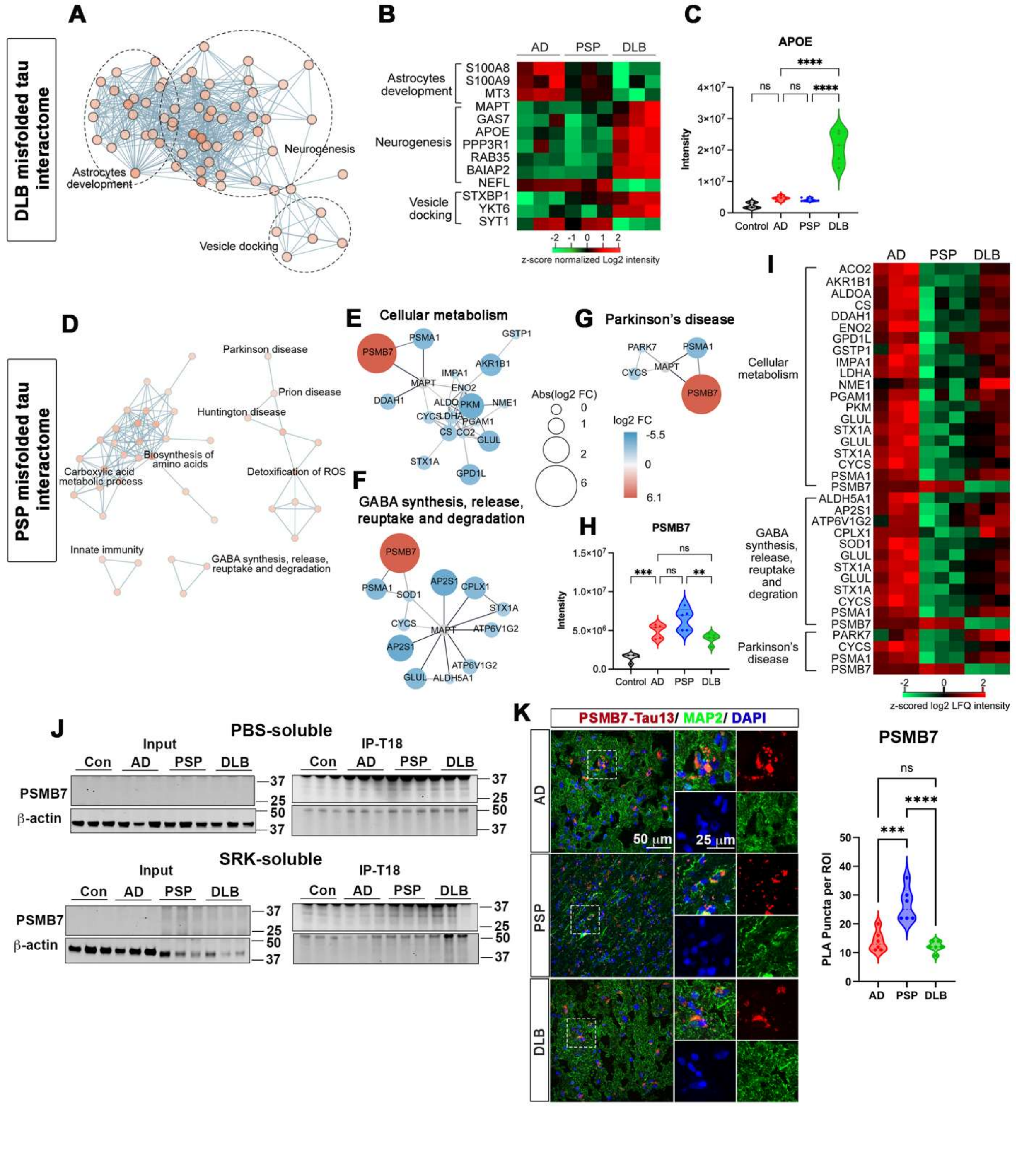

Figure 3. Characterization of the PBS-soluble DLB and PSP tau interactomes.

(A) Functional landscape of the DLB tau interactome. The network map illustrates the functional enrichment of 20 significant proteins either enriched with, or depleted from, misfolded tau immunoprecipitated from DLB cases.

(B) Heatmap displaying the relative abundances of significant DLB tau interactors involved in astrocyte development, neurogenesis, and vesicle docking. Data are presented as z-score–normalized log2 LFQ intensities across AD, PSP, and DLB fractions (ANOVA with permutation-based FDR < 5%).

(C) Quantification of APOE targeted PRM-MS intensity across a new set of Control, AD, PSP, and DLB groups (n=5). Data are presented as violin plots showing the full data distribution, with individual points representing discrete cases across the Con, AD, PSP, and DLB groups (n = 5 independent cases per group). *p < 0.05, p < 0.01, ***p < 0.001, ****p < 0.0001, ns = not significant, determined by one-way ANOVA followed by Tukey's post hoc test.
(D) Functional enrichment network of the 57 significant proteins either enriched with, or depleted from, PSP tau.
(E–G) Sub-networks detailing significant PSP tau interactors implicated in **(E)** cellular metabolism, **(F)** GABA synthesis, release, reuptake, and degradation, and **(G)** Parkinson's disease. Node size correlates with the absolute magnitude of log2 fold change. Node color indicates the log2 FC relative to complement controls (red: enriched in PSP; blue: depleted in PSP), and node size reflects the absolute magnitude.
(H) Quantification of PSMB7 targeted PRM-MS intensity across a new set of Control, AD, PSP, and DLB groups (n=5). Data are presented as violin plots showing the full data distribution, with individual points representing discrete cases across the Con, AD, PSP, and DLB groups (n = 5 independent cases per group). *p < 0.05, p < 0.01, ***p < 0.001, ****p < 0.0001, ns = not significant, determined by one-way ANOVA followed by Tukey's post hoc test.
(I) Heatmap of significant PSP tau interactors highlighted in the sub-networks (**E–G**), shown as z-score–normalized log2 LFQ intensities across AD, PSP, and DLB (ANOVA with permutation-based FDR < 5%).
(J) Biochemical validation of the tau-PSMB7 interaction. Representative WBs of inputs and misfolded-tau (T18) immunoprecipitates (IP-T18) from both PBS-soluble and sarkosyl-soluble (SRK-soluble) brain extracts of Con, AD, PSP, and DLB cases. Blots were probed for PSMB7, with β-actin serving as a loading control.
(K) *In situ* validation of the PSMB7-tau interaction. **Left:** Representative confocal images of PLA showing PSMB7-Tau13 interactions (red puncta) in AD, PSP, and DLB brain tissues. Neurons are counterstained with MAP2 (green) and nuclei with DAPI (blue). Scale bars: 50 μm (main panels) and 25 μm (magnified right panels). **Right:** Quantification of PLA puncta per region of interest (ROI) across the disease groups. (For **C, H, K**) Data are presented as violin plots showing the data distribution, with individual points representing discrete cases (n = 3 independent cases per group). p < 0.01, ***p < 0.001, ****p < 0.0001, ns = not significant, determined by one-way ANOVA followed by Tukey's post hoc test.

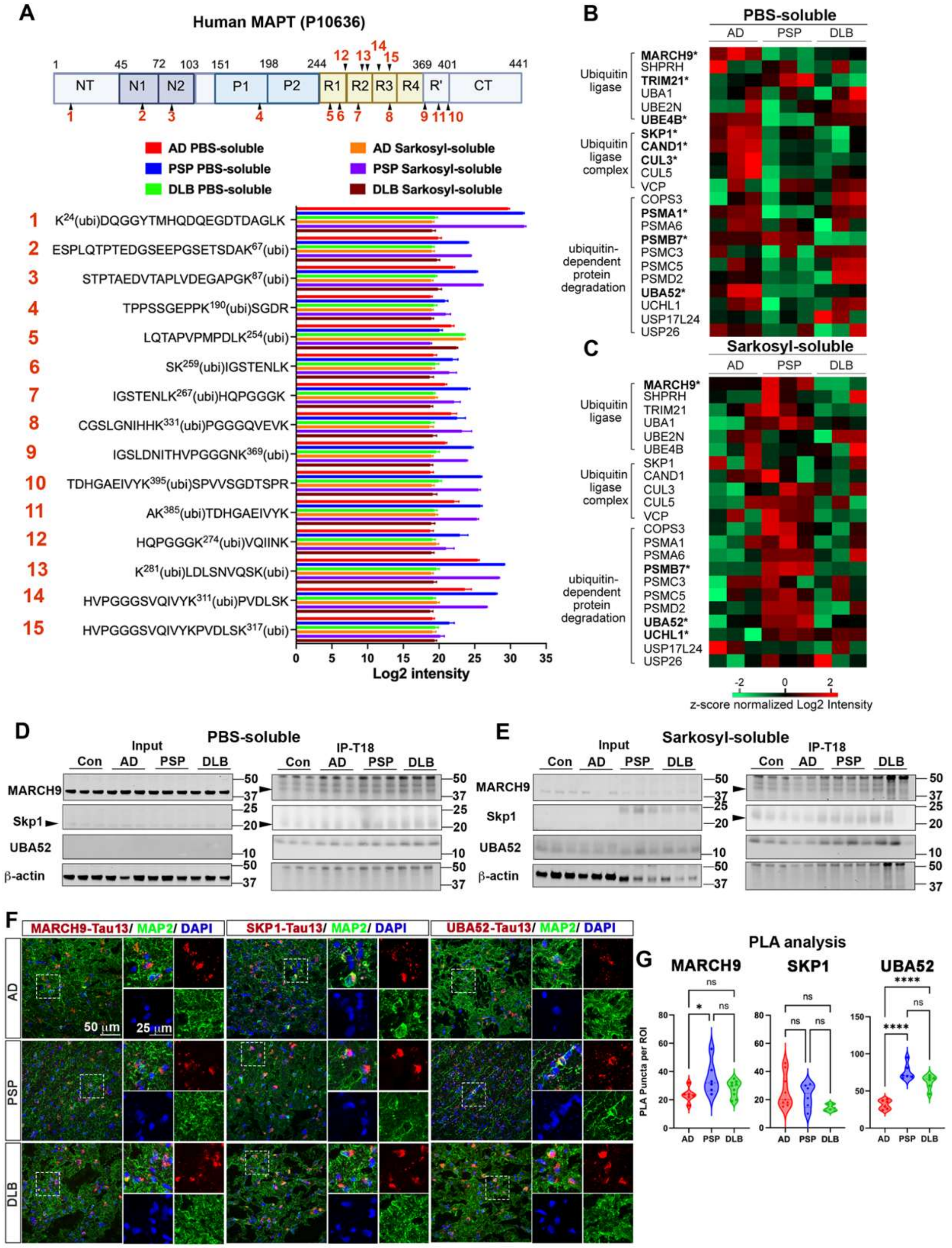

Figure 6. Ubiquitination profiling of tau and validation of ubiquitination-related interactors.

(**A**) Top: Domain map of human tau (MAPT, UniProt P10636) highlighting the locations of 15 identified ubiquitinated lysine residues (red arrows, 1–15). Bottom: Quantitative profiles of the 15 ubiquitinated tau peptides, expressed as log2 intensity, demonstrating significant variance across AD, PSP, and DLB in both PBS- and sarkosyl-soluble fractions (ANOVA with

permutation-based FDR < 0.01).
(B, C) Heatmaps detailing the relative abundance of ubiquitination-related tau interactors in **(B)** PBS-soluble and **(C)** sarkosyl-soluble fractions. Proteins are categorized into ubiquitin ligases, ubiquitin ligase complexes, and ubiquitin-dependent protein degradation pathways. Proteins in bold and marked with an asterisk (*) indicate statistical significance (ANOVA with permutation-based FDR < 0.01). Data are presented as z-score–normalized log2 intensities across AD, PSP, and DLB cases.
(D, E) Biochemical validation of tau interaction with ubiquitination machinery. Representative Western blots of inputs and misfolded tau (T18) immunoprecipitates (IP-T18) from **(D)** PBS-soluble and **(E)** sarkosyl-soluble extracts of Con, AD, PSP, and DLB brains. Blots were probed for MARCH9 (ubiquitin E3 ligase), Skp1 (SCF complex), and UBA52 (ubiquitin-ribosomal fusion protein). β-actin serves as the loading control.
(F) *In situ* validation of protein interactions. Representative confocal micrographs of PLA demonstrating spatial colocalization (red puncta) of Tau13 with MARCH9, SKP1, and UBA52 in AD, PSP, and DLB postmortem brain tissues. Neurons are counterstained with MAP2 (green) and nuclei with DAPI (blue). Scale bars: 50 μm (main panels) and 25 μm (magnified right panels).
(G) Quantification of PLA puncta per region of interest (ROI) for the interactions shown in (**F**). Data are presented as violin plots illustrating the full data distribution (n = 3 independent cases per group). $^*p < 0.05$, $^{****}p < 0.0001$, ns = not significant, determined by one-way ANOVA followed by Tukey's post hoc test.

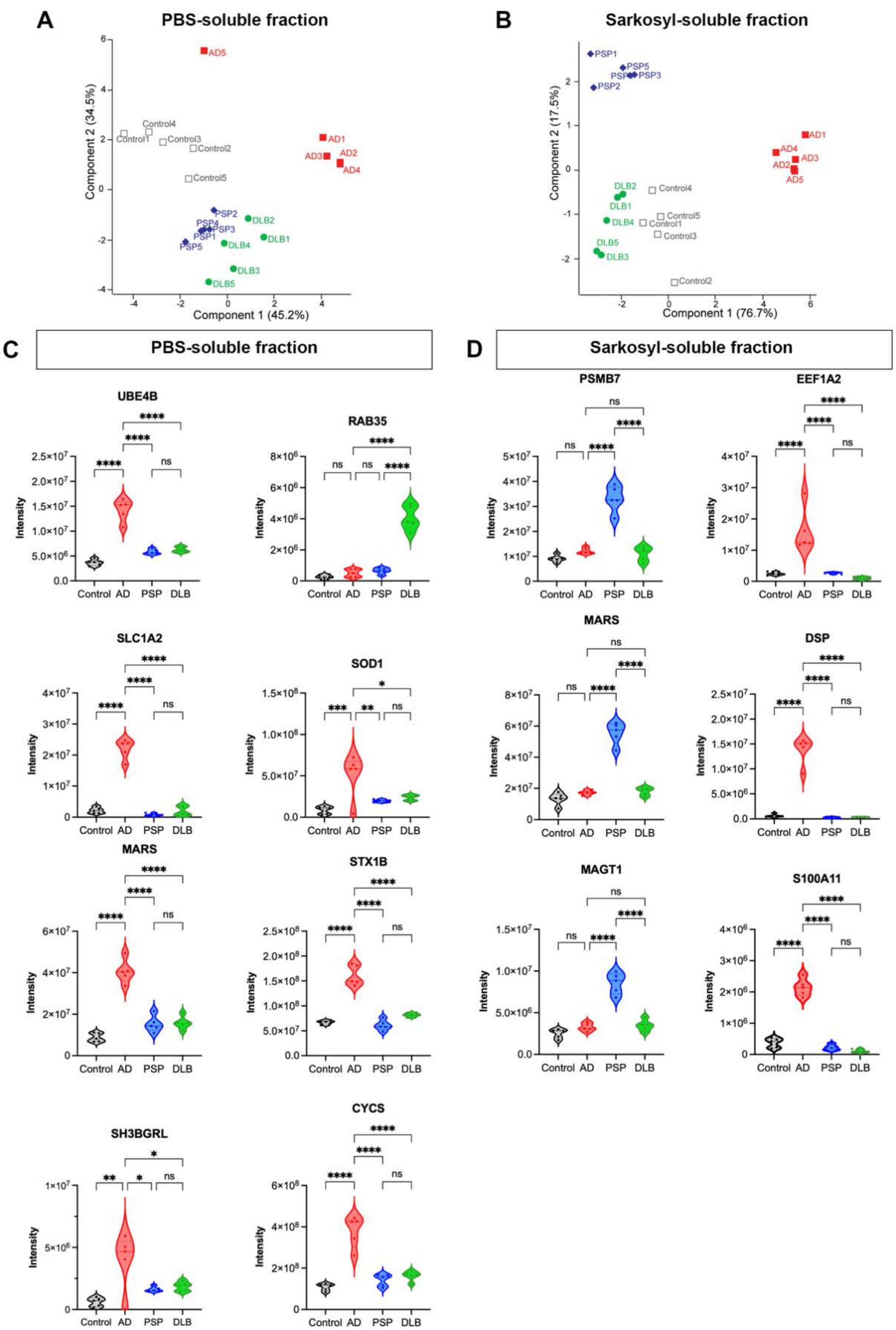

Figure EV3. Validation of targeted misfolded-tau interactors in an independent cohort via PRM-MS.

(**A, B**) Principal component analysis (PCAs) illustrating the distinct segregation of Con (gray), AD, (red), PSP, (blue), and DLB, (green) cases based on their targeted PRM-MS protein profiles. The

distinct clustering demonstrates disease-specific interactomic signatures in both (**A**) PBS-soluble and (**B**) sarkosyl-soluble fractions of the independent validation cohort (n = 5 independent cases per group).

(**C, D**) Targeted quantification of selected significant interactors using PRM-MS in the (**C**) PBS-soluble fraction (UBE4B, RAB35, SLC1A2, SOD1, MARS, STX1B, SH3BGRL, and CYCS) and (**D**) sarkosyl-soluble fraction (PSMB7, EEF1A2, MARS, DSP, MAGT1, and S100A11). Data are presented as violin plots showing the full data distribution, with individual points representing discrete cases across the Con, AD, PSP, and DLB groups (n = 5 independent cases per group). *p < 0.05, p < 0.01, ***p < 0.001, ****p < 0.0001, ns = not significant, determined by one-way ANOVA followed by Tukey's post hoc test.

3. *The machine-learning analysis shows an essentially perfect separation of disease groups using a small set of interactors. However, given the limited sample size, there is a question of overfitting. The conclusions from this analysis should be moderated, and additional validation (for example with an independent cohort) would be required to support the claim of robust disease classification.*

   Response: We have validated the findings with a separate cohort (n=5 per group), as shown in **Fig. EV3, Appendix Table S6**.

4. *The study nominates novel interactors of tau, but there is no functional evidence that these proteins are viable therapeutic targets. The current data support their role as candidate interactors but not as validated targets for intervention. The language should be revised to avoid overstatement and to clearly distinguish between discovery of candidate interactors and identification of therapeutic targets.*

   Response: Thank you for pointing out this important comment. We have revised abstract and the discussion (lines 473-474) to emphasize our key findings that distinct conformations of aggregated tau proteins are associated with disease-specific interaction networks. Our current work lays the foundation for future studies that elucidate the key mechanistic consequences of these disease-specific interactions.

<u>Minor concerns</u>

1. *The criteria for selecting proteins for validation should be described more explicitly.*
   Response: Thank you for the comment. The targeted PRM-MS was to identify the top 10 interactors in PBS-and sarkosyl-soluble in separate cohort (n=5/group, **Fig. EV3, Appendix Table S6**). The selected proteins were selected for Co-IP Western blot and PLA studies based on their disease-specific enrichment with tau from the Top 10 features of Disease-relevant misfolded tau interactors in PBS-soluble and sarkosyl-soluble fractions (**Table 2**).

2. *Quantitative details of reproducibility between replicates and across disease groups should be presented more clearly.*

Response: We have provided additional data to demonstrate reproducibility between replicates and across disease groups below.

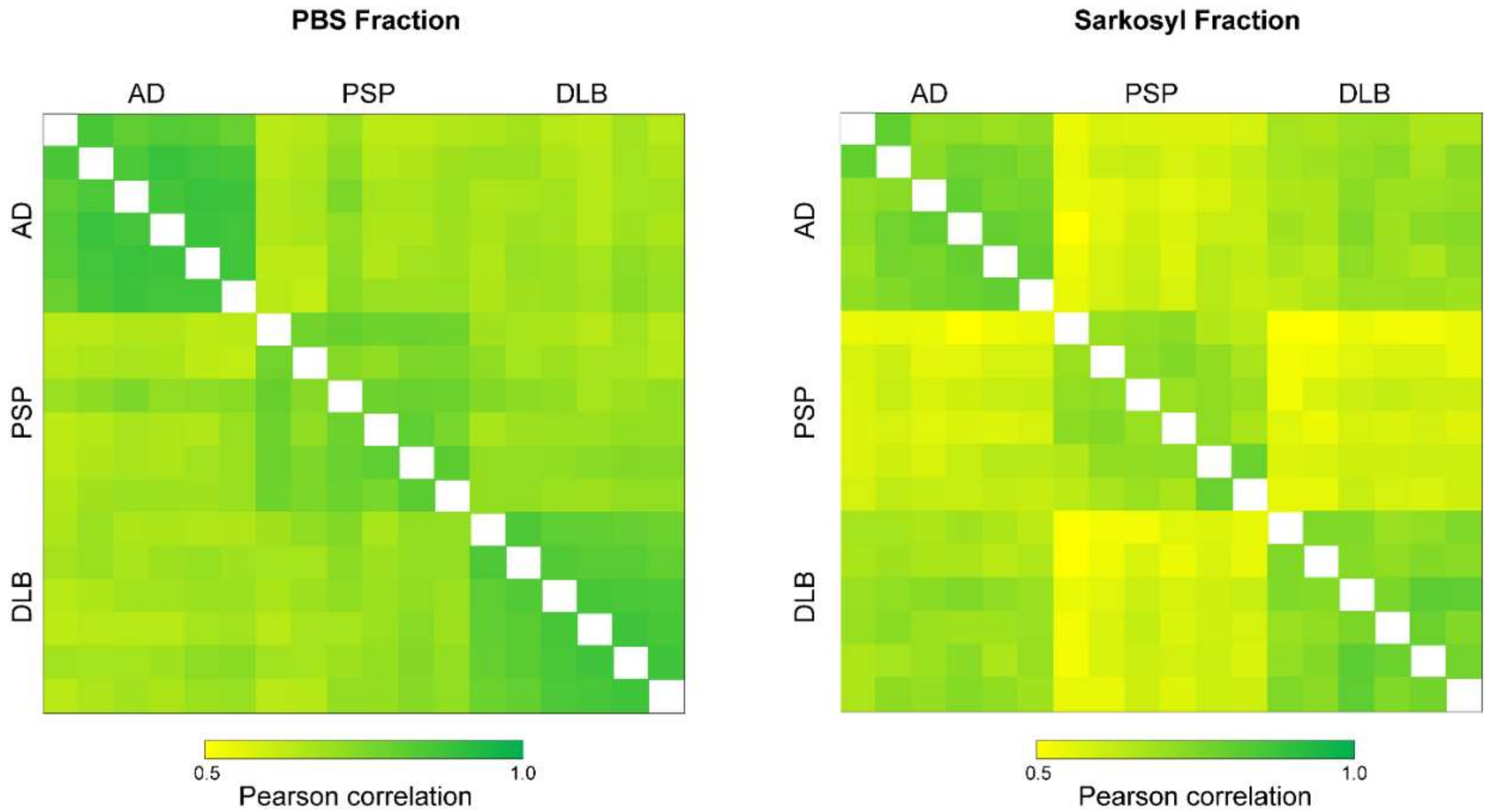

Proteomic reproducibility across biological replicates and disease cohorts. Global Pearson correlation heatmaps of protein LFQ intensities comparing biological replicates within and across AD, PSP, and DLB cohorts. Correlation matrices are shown for both the **(Left)** PBS-soluble and **(Right)** sarkosyl-soluble fractions (n = 5 independent cases per disease group). The color gradient reflects the PCC (*r*), ranging from 0.5 (yellow, lower correlation) to 1.0 (dark green, high correlation). The white diagonal represents absolute self-correlation (*r* = 1.0). Distinct dark green modular blocks along the diagonal demonstrate robust intra-group reproducibility among biological replicates, while the lighter off-diagonal regions highlight the divergence of the tau interactome across different proteinopathies.

3. *Some figure legends should be clearer, particularly regarding which interactions are directly confirmed by PLA or dot blot.*

Response: Thank the reviewer's suggestion. We have made the changes as suggested.

4. *Statistical details should be consistently reported in the figure legends.*

Response: Thank the reviewer's suggestion. We have made the changes as suggested.

<u>**Additional suggestions (non-essential)**</u>

As touched upon above, the following point may be considered:
1) Including additional cases or brain regions, if available, would strengthen the robustness of the interactome differences reported.
Response: Thank you for the comment. To strengthen the robustness of the interactome differences, we have validated the significant results in a separate cohort (n=5 per group) in

addition to the cohort present in the manuscript, using parallel reaction monitoring-mass spectrometry (PRM-MS) analysis, as shown in **Fig. EV3, Appendix Table S6.**

2) Exploring alternative capture antibodies (targeting distinct tau epitopes) could help rule out potential epitope-driven biases in the interactomes.

Response: Thank you. We have shown the use of T18 to distinguish soluble tau in several of our previous works (PMID: 39809992, 32737202, 40823851, 40681153, 35202653, 39239152, 38135105, 33246331, 41610849, 39688618). Moreover, we have provided the IHC staining using T18 of AD, PSP, and DLB. The focus of our study was to evaluate the distinct conformations of aggregated tau proteins that are associated with disease-specific interaction networks, a critical knowledge gap within the field.

3) Functional experiments (perturbing candidate interactors in cellular or animal models) would considerably strengthen the therapeutic relevance of the findings. The authors should at least acknowledge these further studies.
Response: Thank you for the comment. We have included suggested changes in limitations paragraph (Lines 452-472).

Referee #2:

Puangmalai and colleagues present a thorough and thoughtfully designed comparative proteomic analysis investigating how distinct conformations of tau aggregates drive disease-specific molecular signatures across Alzheimer's disease (AD), progressive supranuclear palsy (PSP), and dementia with Lewy bodies (DLB). Through conformation-specific immunoprecipitation of misfolded tau (T18 antibody) from PBS- and sarkosyl-soluble brain fractions, followed by label-free LC-MS/MS, the authors identify nearly 500 high-confidence tau interactors with a high disease specificity and no common interactors overlapping across all three tauopathies. Selected candidates are validated via Western blotting and proximity ligation assays (PLA). Functional enrichment and network analyses, including machine learning-based classification, reveal clearly distinct functional landscapes among the diseases.

The study provides strong evidence that tau polymorphs adopt distinct conformations that define unique protein interaction networks, thereby contributing to disease-specific cellular dysfunctions. These findings meaningfully advance our understanding of tauopathy heterogeneity and have the potential to inform future biomarker development and precision therapeutic approaches in neurodegeneration.

<u>Major Points</u>

1. **Statistical Power**
The use of ANOVA and FDR-controlled analyses is appropriate; however, the relatively small number of biological replicates (three individuals per disease, with two technical replicates each) limits statistical robustness. This constraint should be explicitly acknowledged and discussed in the manuscript.

Response: Thank you for the comment. We have validated the significant findings using an additional separate cohort (n=5 per group), shown in **Fig. EV3, Appendix Table S6.**

2. Specificity and Background Controls
Incorporating additional controls would further strengthen the conclusions:
◦ ***Healthy controls:*** *Although healthy controls should not show marked tau aggregates in the sakroyl-soluble fractions, a comparison with age-matched non-diseased brains (for both soluble and sarkosyl-soluble fractions) would nevertheless help assess the disease specificity of identified interactors.*

Response: Thank you for the comment. We have included healthy controls in our validation study.

◦ ***Matrix/IgG control:*** *Inclusion of a matrix-only or IgG control would help discriminate specific from nonspecific binding events. Without such controls, differences in aggregate composition could influence the apparent interactomes by introducing proteins that bind nonspecifically to the matrix. Although such information can still be biologically informative, it complicates interpretation of specific protein-tau associations and challenges the designation of these proteins as bona fide "tau-interacting."*

Response: We used a Hawaii plot with a complement negative control, an advanced multi-volcano plot for analyzing large-scale affinity purification–mass spectrometry (AP-MS). This method allows for a global statistical comparison of multiple AP-MS experiments against a single, robust negative control. The complement-negative control strategy used a composite control that was created from the experimental data itself rather than a single dedicated control sample. For the Tau pulldown protein experiment from AD, DLP, and PSP brains, an internal negative control is assembled from the combined pull-downs of all experiments, which better reflects the average background binding across the entire dataset. The Hawaii plot then depicts each AP pull-down relative to this unique, internally generated complement-negative control, thereby accurately identifying genuine protein interactions and eliminating the matrix effect.

Minor Points

a) *The number of biological and technical replicates should be clearly stated in both the main text and figure legends, in accordance with journal guidelines.*

Response: Thank you for the suggestion. We have made the changes as suggested.

b) The release date and version of the UniProt/SwissProt database used for protein identification should be reported.

Response: Thank you for the suggestion. We have made the changes as suggested in the Materials and Methods section (Line 596).

c) While the data-sharing statement regarding RAW data is understandable given patient privacy concerns, providing the complete MaxQuant output tables would enhance transparency as it facilitates to reproduce the data analysis.

Response: Thank you for the comment. We have provided a ZIP file containing the MaxQuant output tables as supplemental data.

Overall Assessment

This is a high-quality and carefully executed study offering valuable mechanistic insight into disease-specific tau interactomes. The results are well presented and interpreted. With

clarification of statistical limitations and inclusion or discussion of appropriate specificity controls, the manuscript would be well suited for publication in The EMBO Journal.

Response: Thank you for the encouragement.

Referee #3:

The manuscript by Puangmalai et al. investigates interactome differences for misfolded Tau between tauopathies. The authors provide a large amount of very interesting Mass Spec data, that informs about differences of oligomeric Tau, recognized by T18 antibody, interactions in AD, PSP, and DLB. Furthermore, they show that different Tau fragments are more or less abundant in these diseases, and that they are differentially posttranslational modified (Ub, Ac, Ph). Unfortunately, these two parts appear quite disconnected from each other. Beyond the data provided, the authors do not provide mechanistic data on why the interactomes are different, and how they differentially shape Tau function and neuronal pathways. Therefore, although the authors use a logical experimental approach to assess the tau interactome, several points would need clarification and improvement. Unfortunately, I cannot support publication in EMBO Journal in the current state of the manuscript.
To improve the manuscript and add causal links between interactomes and Tau fragments/PTMs, the authors could try to show that different interactors bind to certain Tau fragments and are dependent on certain PTMs, and that pathways, in which the identified interactors are involved, are differentially dysregulated in the different diseases. It would. Make sense to focus on no more than 1 or 2 pathways then.

<u>Major points</u>

1. I understand that the authors do a comparative analysis of what is pulled does with T18 in different tauopathies. There is no data shown, however, how specific the AB is for Tau and for oligomeric Tau. How do we know that the pull down is not just pulling total Tau interactomes?

Response: Thank you for the comment. We have shown the use of T18 to distinguish soluble tau in several of our previous works (PMID: 39809992, 32737202, 40823851, 40681153, 35202653, 39239152, 38135105, 33246331, 41610849, 39688618). Moreover, we have provided the IHC staining using T18 of AD, PSP, and DLB below:

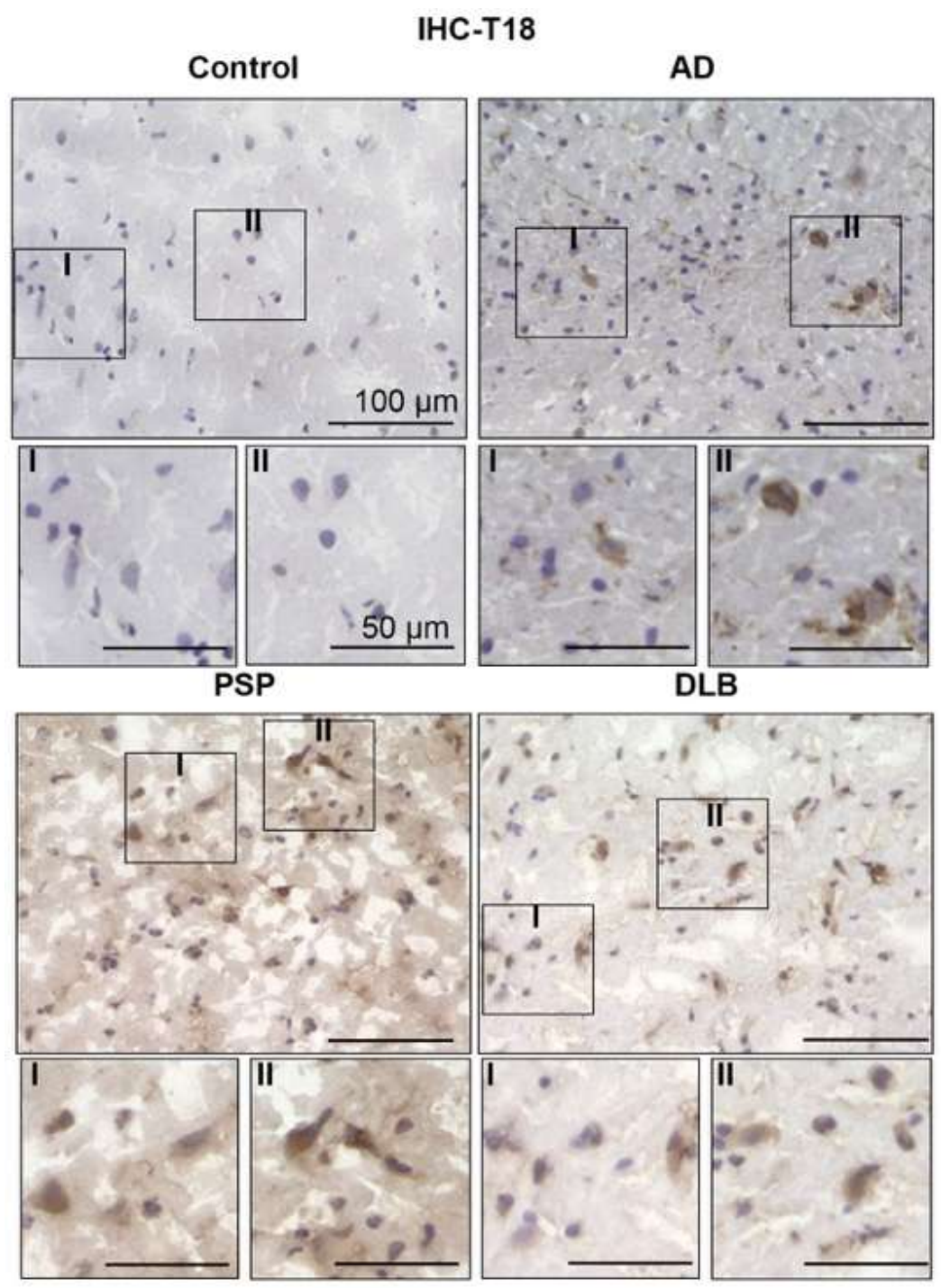

Immunohistochemical validation of T18 antibody specificity for pathological misfolded tau. Representative brightfield micrographs of postmortem brain tissue from cognitively normal Con, AD, PSP, and DLB cases immunostained with the T18 anti-tau antibody (brown). Nuclei are counterstained with hematoxylin (blue). The Control tissue demonstrates a complete absence of T18 immunoreactivity, confirming the antibody does not cross-react with highly abundant physiological monomeric tau. In contrast, prominent T18-positive pathological tau inclusions are visible across the AD, PSP, and DLB cohorts. Boxed regions (**I** and **II**) in the main images are shown at higher magnification in the corresponding bottom panels to highlight distinct intracellular tau pathology. Scale bars: 100 μm (main panels) and 50 μm (magnified insets).

2. The T18 Tau interactome should be prepared for control brain cases as well.

Response: Thank the reviewer's comment. We have included healthy controls in our validation study.

3. *I may have missed it, but I could not find a volcano plot or other analysis comparing the input proteomes (without T18 IP) between diseases (PBS/Sarkosyl). Are they similar or different between diseases? - this would influence the T18 interactome.*

Response: We conducted the proteome of the brain frontal cortex and found that these results suggested that the significant changes observed in misfolded tau pull-downs were caused by alterations in protein-protein interactions, rather than differential expression levels in the brains of patients with AD, PSP, and DLB (**Fig. EV1M, N**). We have provided volcano plots of the proteome of the brain frontal cortex below**.**

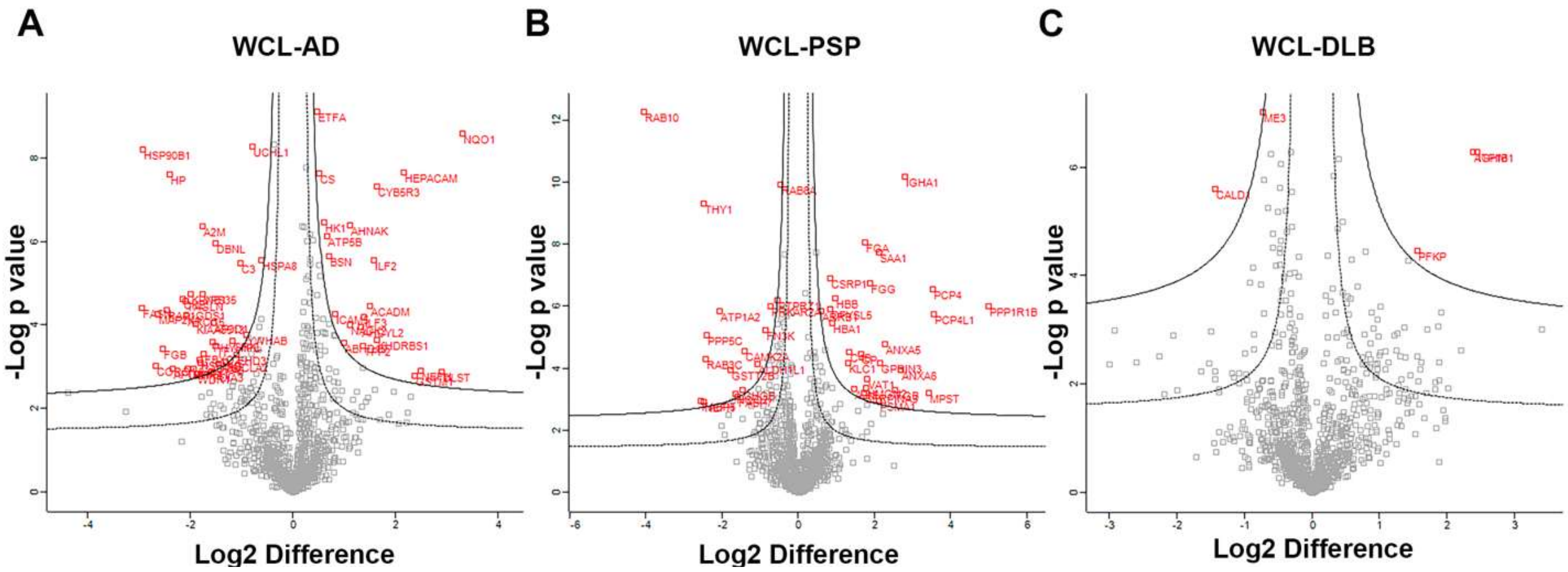

Global proteomic profiling of input brain homogenates across disease cohorts.

(**A–C**) Volcano plots illustrating the differential protein expression in the whole cell lysate (WCL) input proteome of the frontal cortex—prior to T18 immunoprecipitation—from **(A)** AD**, (B)** PSP, and (**C**) DLB cases. A Hawaii plot analysis with a complement control was conducted. The plots display the log2 fold difference (x-axis) against the negative log10 p-value (y-axis). Proteins exhibiting statistically significant changes in overall expression levels are highlighted in red with their corresponding gene names, while non-significant background proteins are represented by grey markers. Solid and dashed curves denote statistical significance thresholds (permutation-based FDR). These input proteome profiles demonstrate the baseline global protein abundance, supporting the conclusion that the distinct interactomic signatures observed in the misfolded-tau pull-downs are driven primarily by disease-specific alterations in protein-protein interactions rather than differential global protein expression.

4. A Western Blot showing total Tau in the input and pulldown fractions of the IP fractions would be needed to show that Tau actually was pulled down and eluted from the T18 column.

Response: In our interactome study, we identified tau with high sequence coverage, indicating that the tau protein was successfully pulled down and eluted from the T18 column. In addition, we have shown the WB of Tau 13 to demonstrate successful pull down below.

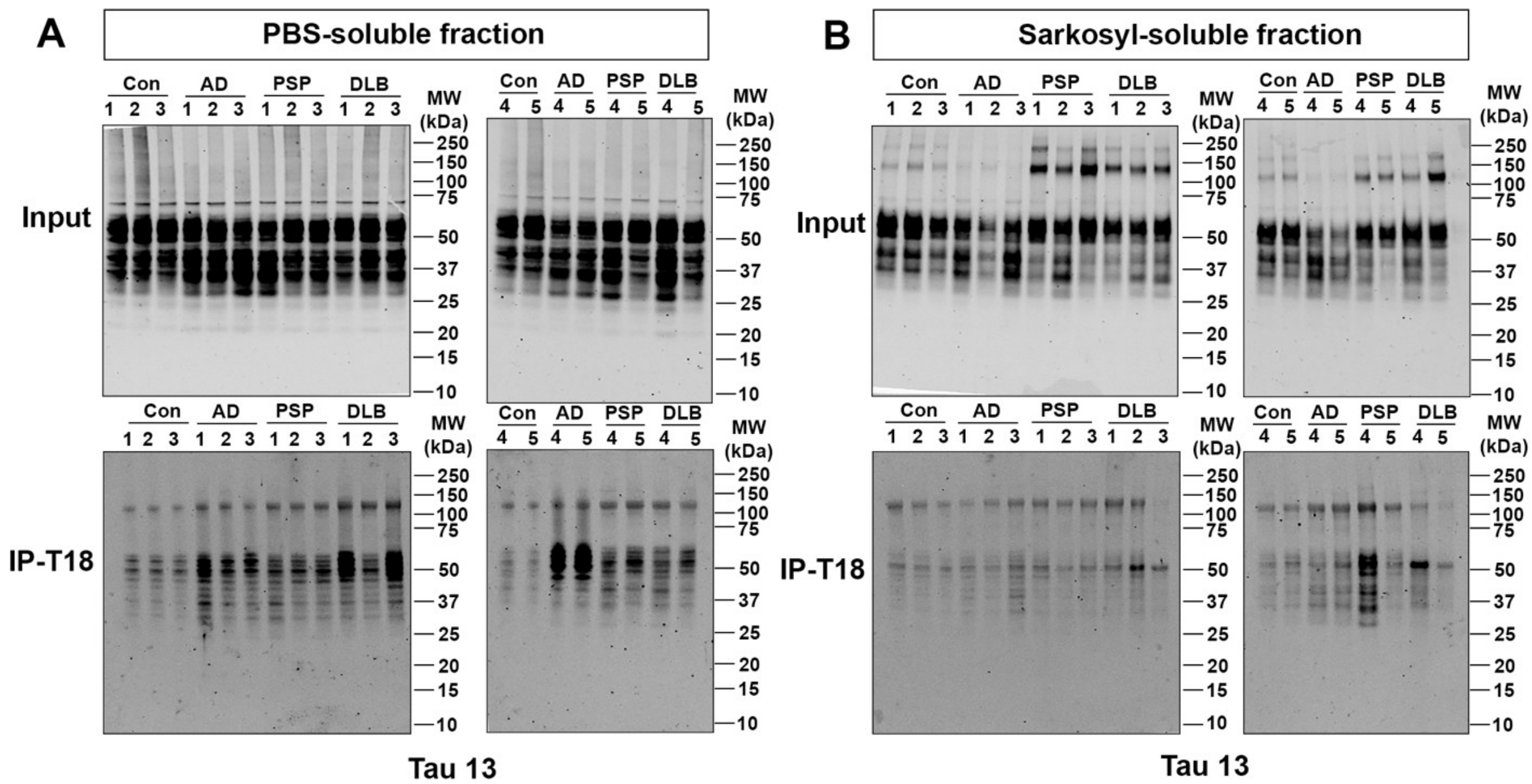

Validation of tau immunoprecipitation and elution by the T18 column in distinct solubility fractions. (**A-B**) Representative Western blots demonstrating total tau levels in the initial brain lysates (Input, top panels) and the subsequent T18 immunoprecipitated elution fractions (IP-T18, bottom panels). Assays were performed on both (**A**) PBS-soluble and (**B**) Sarkosyl-soluble fractions derived from post-mortem human brain tissue. Blots were probed with the pan-tau antibody Tau 13. To ensure reproducibility, samples were analyzed from a new, independent validation cohort consisting of neurologically Con, AD, PSP, and DLB cases (n = 5 cases per group; individual biological replicates are numbered 1–5 across the split membranes). The presence of tau bands in the IP-T18 fractions across all disease states and solubility conditions confirms the successful capture and elution of total tau from the T18 column.

5. PLAs in Fig 2F-I, Fig 3 G+H, Fig 6H-K are not convincing at all. 1) There are no PLA "dots" to see and the images are full of background and image processing artifacts - the imaging resolution is obviously not good enough and the background needs to be quenched. 2) why has the PLA been done with Tau13 (a total human Tau AB) and not with T18? - 3) PLA is not the right way to show the amount of interaction, esp. when having two soluble proteins in the same cell compartment, the crowded cytosol. PLA can show where interactions happen and has a proximity radius of ~20-30 nm, so it will give signal mostly everywhere in the cytosol. The authors should replace the PLA data with classic co-IP and Western Blot to show increased or decreased interactions of Tau with the selected targets. 4) co-staining with neuronal marker would be necessary to show what we are looking at here.

Response: Thank you for these constructive comments regarding the PLA results.

1) We and others have used the PLA method to confirm the protein-protein interaction in human and mouse brain tissues, which is not always shown as PLA dots, such as the results in Jensen et al., 2024 (PMID 39613827), due to the high abundance of the target proteins and their protein-protein interaction. Regarding this matter, we quantified PLA area (puncta per field) as an appropriate approach rather than dot counts.

2) For the quality control of PLA results, we have included secondary antibody-only staining (**Fig. EV2C**) to determine the non-specific or background staining. All the images were taken from high-resolution confocal microscopy; however, we have ensured that we adjust the image resolution up to 600 dpi for submission.

3) PLA analysis was performed to validate the MS results of the total tau interactome using Tau13, disregarding their solubility.

4) Thank you for the reviewer's comment on the co-staining with the neuronal marker (MAP2). We have made the change as suggested.

6. *It is absolute unclear why the authors use the term "Tau polymorph" in the title as well as in the manuscript. Polymorph comes from morphology = appearance, poly = many. The authors use one kind of conformation/assembly specific antibody, T18, to fish out Tau interactors. Means, the differences in interactors they find are disease-specific for one kind of Tau assembly/conformation/morphology type. The nomenclature "Tau polymorphs" is misleading. The title should be changed in this regard as well.*

Response: Thank you for the suggestion, however, the authors feel the word polymorph is appropriately used as soluble tau from AD, PSP and DLB have been shown to have different conformations, morphologies, seeding activities, affect neuronal functions, and modulate gene expression differently in excitatory neurons (PMID 39809992). Moreover, fibrillar tau from several neurodegenerative diseases show different conformations at an atomic level (PMID: 34588692). Additionally, analysis of protease cleavage is a widely used technique for distinguishing different conformations for amyloid proteins (PMID: 9771749, 39809992, 40681153, 39145409, 38503425, 38503425, 32350746). Our Tau peptide compositions show that T18-IP AD, PSP, and DLB derived tau exhibit distinct conformations.

7. The Tau fragment analysis include PTM analysis, is currently disconnected from the rest of the MS. It is not clear, why the authors did these analyses. I guess the aim was to understand which Tau fragments may be relevant for the interactions identified, and, additionally, how PTMs of Tau influence the interactions. However, this at the moment is not being analyzed, and the Tau fragment data - although very interesting and complex - provide no mechanistic insides into how they shape the Tau interactome, or how they may be connected to Tau conformation/assembly detected by the T18 antibody. If the authors could exemplify how the Tau fragment and PTMs identified influence the interaction of Tau with one or two important interactors, this would certainly boost the impact. At the current state, it seems to remain a collection of interesting observations that are too complex to grasp and therefore remain unclear.

Response: We appreciate the reviewer's careful evaluation of our fragment and PTM analyses. The primary objective of the trypsin susceptibility (fragment) and PTM analyses was to provide orthogonal, structural evidence that the T18 antibody captures fundamentally distinct tau polymorphs in AD, PSP, and DLB (PMID: 39809992). By demonstrating that these aggregates expose different lysine residues to trypsin and possess unique PTM signatures, we establish that their physical conformations differ. We hypothesize that it is this overarching conformational divergence, rather than isolated, single-residue modifications, that acts as the primary driver of the distinct interactomes we observe. For example, in our study, AD tau uniquely enriches for the E3 ligases MARCH9 and UBE4B in the PBS-soluble fraction, and exhibits specific patterns of ubiquitination. Though this study did not include Cryo-Electron microscopy, previous studies have reported that ubiquitination modifications mediate structural diversity in tauopathies (PMID: 32032505).

Minor Points

1. The authors identify different Tau peptide composition in AD, PSP, LBD cortex. This is very valuable information; however, the authors do not describe or discuss the meaning of these data. More discussion of MS data for Tau fragments in AD/PSP/DLB (Fig. 1 and S1). Also, Data/Figure for Tau fragment analysis shown in Fig 1 should be shifted in front of ubiquitination/PTM analysis of Tau fragments in Fig 6.

Response: Thank the reviewer's suggestion. As mentioned above, the use of protease cleavage is critical technique for distinguishing distinct confirmations (PMID: 9771749, 39809992, 40681153, 39145409, 38503425, 38503425, 32350746). Regarding the reorganization of the figures, we carefully considered the reviewer's logical suggestion to group the fragment analysis (**Fig. 1**) with the PTM analysis (**Fig. 6**), as both describe the structural features of the tau polymorphs. However, we respectfully opted to retain the fragment data in Figure 1 to preserve the chronological narrative of our experimental rationale.

2. Please provide the protein names of the most deregulated proteins in Fig 1J-P. This would be useful for all enriched /depleted interactors for all three diseases, e.g., as an additional Supplementary Table.

Response: Thank the reviewer's suggestion. We will make changes as suggested.

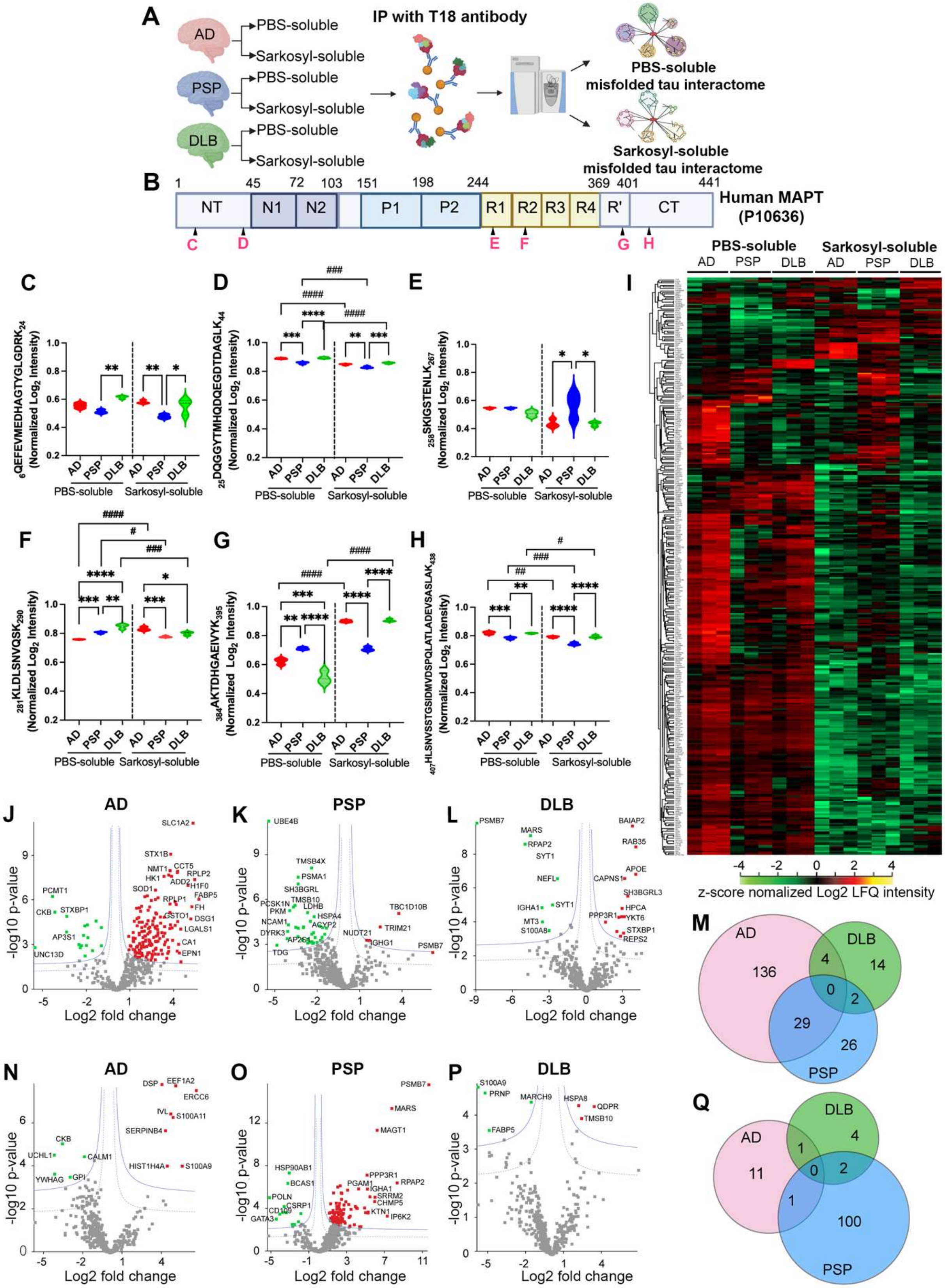

A
AD
PSP
DLB
PBS-soluble
Sarkosyl-soluble
IP with T18 antibody
PBS-soluble
misfolded tau interactome
Sarkosyl-soluble
misfolded tau interactome
B
1 45 72 103 151 198 244 369 401 441
NT N1 N2 P1 P2 R1 R2 R3 R4 R' CT
Human MAPT
(P10636)
C D E F G H
C
$_{6}$QEFEVMEDHAGTYGLGDRK$_{24}$
(Normalized Log$_2$ Intensity)
D
$_{25}$DQGGYTMHQDQEGDTDAGLK$_{44}$
(Normalized Log$_2$ Intensity)
E
$_{258}$SKIGSTENLK$_{267}$
(Normalized Log$_2$ Intensity)
F
$_{281}$KLDLSNVQSK$_{290}$
(Normalized Log$_2$ Intensity)
G
$_{384}$AKTDHGAEIVYK$_{395}$
(Normalized Log$_2$ Intensity)
H
$_{407}$HLSNVSSTGSIDMVDSPQLATLADEVSASLAK$_{438}$
(Normalized Log$_2$ Intensity)
AD PSP DLB AD PSP DLB
PBS-soluble Sarkosyl-soluble
I
PBS-soluble Sarkosyl-soluble
AD PSP DLB AD PSP DLB
-4 -2 0 2 4
z-score nomalized Log2 LFQ intensity
J AD
SLC1A2 STX1B NMT1 CCT5 RPLP2 HK1 ADD2 H1F0 SOD1 FABP5 RPLP1 FH GSTO1 DSG1 LGALS1 CA1 EPN1 PCMT1 CKB STXBP1 AP3S1 UNC13D
K PSP
UBE4B TMSB4X PSMA1 SH3BGRL PCSK1N TMSB10 PKM LDHB NCAM1 HSPA4 ACYP2 DYRK3 AP2B1 TDG NUDT21 TBC1D10B TRIM21 IGHG1 PSMB7
L DLB
PSMB7 MARS RPAP2 SYT1 NEFL IGHA1 SYT1 MT3 S100A8 BAIAP2 RAB35 APOE CAPNS1 SH3BGRL3 HPCA PPP3R1 YKT6 STXBP1 REPS2
-log10 p-value
Log2 fold change
M
AD DLB PSP
136 4 14 0 2 29 26
N AD
DSP EEF1A2 ERCC6 IVL S100A11 SERPINB4 HIST1H4A S100A9 CKB UCHL1 CALM1 YWHAG GPI
O PSP
PSMB7 MARS MAGT1 HSP90AB1 BCAS1 PPP3R1 RPAP2 PGAM1 IGHA1 SRRM2 CHMP5 KTN1 IP6K2 POLN CD109 CSRP1 GATA3
P DLB
S100A9 PRNP MARCH9 HSPA8 QDPR TMSB10 FABP5
Q
AD DLB PSP
11 1 4 0 2 1 100

Figure 1. Identification of disease-specific PBS- and sarkosyl-soluble tau interactomes in postmortem brains.

(A) Schematic workflow of the IP-MS approach used to isolate and quantify the disease-specific misfolded-tau interactome. Misfolded tau was immunoprecipitated from PBS-soluble and sarkosyl-soluble fractions of AD, PSP, and DLB human brain tissues using the conformation-specific T18 antibody, followed by LC-MS/MS (n = 3 independent cases per group). **(B)** Domain map of the longest human tau isoform (2N4R; UniProt P10636), detailing the N-terminal region (NT), inserts (N1, N2), proline-rich regions (P1, P2), microtubule-binding repeats (R1–R4 and R′), and the C-terminus (CT). Magenta letters (**C–H**) map the precise locations of the representative tryptic peptides quantified below.

(C–H) Quantitative profiles of representative misfolded-tau peptides captured in the pull-downs. Data are presented as violin plots showing the normalized log2 LFQ intensities in PBS- and sarkosyl-soluble fractions from AD (red), PSP (blue), and DLB (green) cases. *$p < 0.05$, $p < 0.01$, ***$p < 0.001$, ****$p < 0.0001$ for pairwise differences across diseases within the same fraction; $^{\#}p < 0.05$, $^{\#\#}p < 0.01$, $^{\#\#\#}p < 0.001$, $^{\#\#\#\#}p < 0.0001$ for differences between PBS- and sarkosyl-soluble fractions within the same disease, determined by two-way ANOVA with Tukey's post hoc test. **(I)** Hierarchical clustering heatmap of all significant proteins identified from the T18 immunoprecipitation (ANOVA with permutation-based FDR < 5%). Data are displayed as z-score–normalized log2 LFQ intensities.

(J–L, N–P) Volcano plots illustrating the statistical significance versus magnitude of change for proteins quantified from misfolded-tau pull-downs in the **(J–L)** PBS-soluble and **(N–P)** sarkosyl-soluble fractions of AD, PSP, and DLB brains. Significant interactors were determined using a permutation-based false discovery rate (FDR). Solid and dashed blue lines denote high-confidence (Class A, 1% FDR) and lower-confidence (Class B, 5% FDR) thresholds, respectively. Red dots represent significantly enriched proteins (Class A, 1% FDR), green dots represent significantly depleted proteins (Class A, 1% FDR), and grey dots denote non-significant background proteins.

(M, Q) Venn diagrams detailing the intersection of significant misfolded-tau interacting proteins across the AD, PSP, and DLB cohorts in the **(M)** PBS-soluble and **(Q)** sarkosyl-soluble fractions.

3. Please clarify protocol for Sarkosyl soluble fraction in Methods; clearly state which fraction was taken for IP

Response: Thank the reviewer's suggestion. We have made the changes as suggested in Methods.

4. There are very few interactors of Tau pulled down with T18 in the sarkosyl-soluble IP. Obviously, the Sarkosyl can denature proteins and prohibit interactions. Did the authors verify that T18 is binding Tau in 1% Sarkosyl?

Response: Thank you. Results in **Fig. EV1A** demonstrated the total tau intensity of IP with T18 antibody in the sarkosyl-soluble fraction. In addition, we have shown the WB of Tau 13 to demonstrate successful pull down above in point 4.

5. *Double check Ref 66, please*

Response: Thank you. We have corrected the reference.

6. Fig 1M > the Venn diagram seems to not match the volcano plots in J, K, L. Please double check

Response: Thank the reviewer's suggestion. We have double checked Fig. 1M and the Venn Diagram.

7. Please list the significant enriched proteins in the Figure, so that readers can easily access. For the string networks, please state clearly what you are showing.

Response: Thank the reviewer's suggestion. We have made the changes as suggested.

8. Please make the way, how interactors are shown for the different diseases, more uniform. It is confusing that sometimes are STRING networks and sometimes heatmaps are shown. Also, include all significant interactors in the heat maps.

Response: Thank the reviewer's suggestion. We have made the changes as suggested.

9. Please provide a list (Supp Table and listed in Figure for PSP and DLB) of significant enriched and depleted interactors, so the reader has an easier access to the data/findings.

Response: These significantly enriched and depleted interactors have been provided as supplemental tables.

10. Fig 4, sarkosyl soluble fraction interactomes, please show the heatmap of significant enriched/depleted interactors across disease, like for PBS-soluble interactors in Fig. 2.

Response: Thank the reviewer's suggestion. We have made the changes as suggested.

11. Please also show interactors common between diseases in the String networks (e.g., different shading), to make the reader aware of all interactions happening.

Response: Thank the reviewer's suggestion. We have made changes as suggested.

12. Our lab has not much expertise in machine learning, but is there a way to show significance for the difference of the 10 signature proteins between the disease in Fig 5B and F?

Response: The ranking of the significance of the 10 signature proteins between the disease in **Fig. 5B** and F are listed in **Table 2**.

13. PTM analysis of Tau fragments, currently does not add much to the manuscript story and should be moved to supplemental material.

Response: We thank the reviewer for this feedback. We carefully considered moving this analysis to the supplemental material; however, we respectfully elected to retain it in the main text because it forms a critical pillar of our overarching hypothesis. As detailed in our response to *Comment 7*, the trypsin susceptibility and PTM profiles provide the essential orthogonal evidence that the misfolded tau aggregates captured by the T18 antibody are fundamentally distinct polymorphs across AD, PSP, and DLB. Removing the PTM and fragment data from the main text would disconnect the conformational identity of the tau polymorphs from the distinct interaction networks

they assemble. We believe retaining this data in the main figures is necessary to provide readers with a complete, cohesive narrative.

Additional Revised Figures:

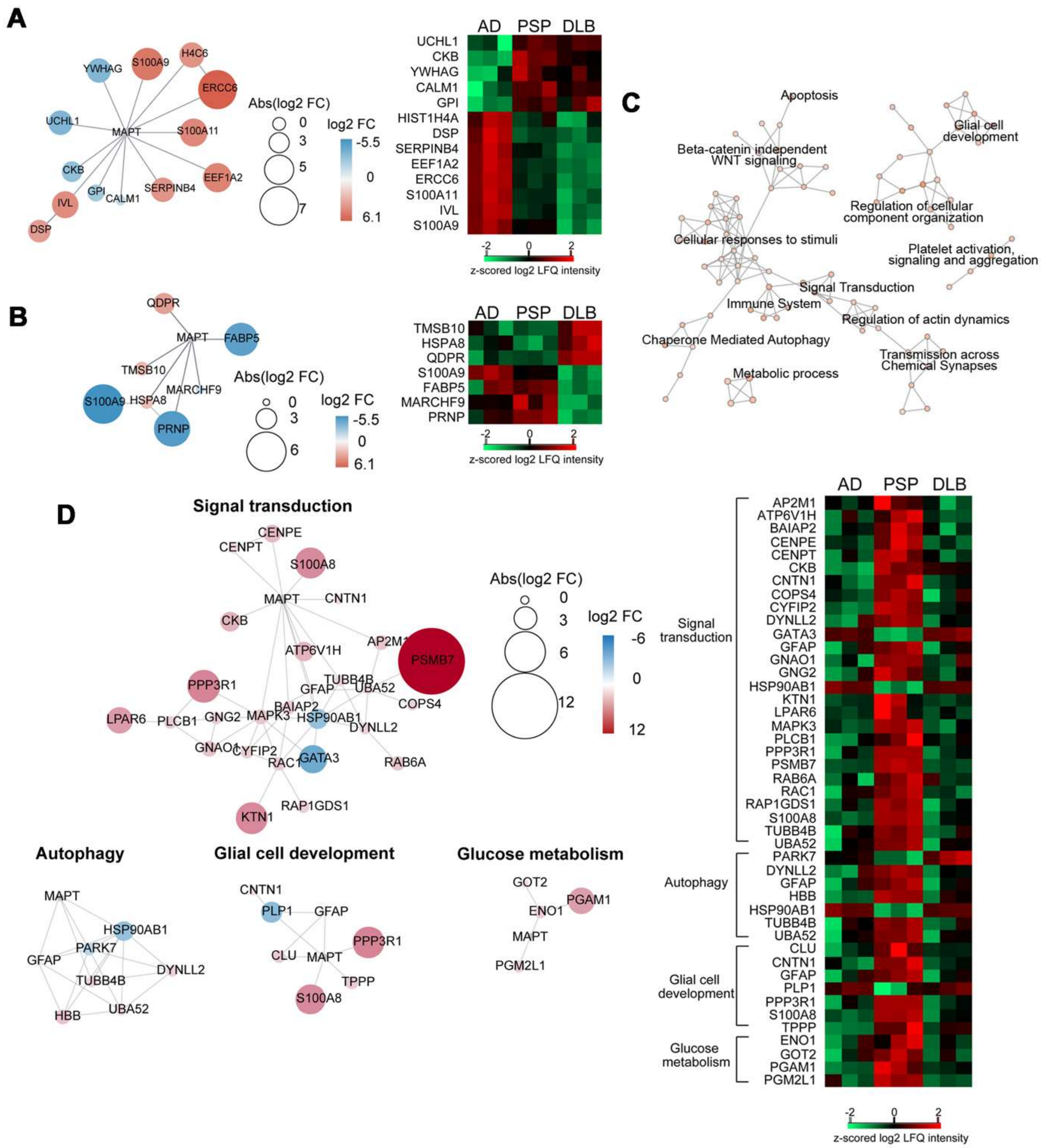

Figure 4. Characterization of disease-specific sarkosyl-soluble tau interactomes.

(A) Left: Protein interaction network of significant sarkosyl-soluble AD tau interactors in the fraction of AD brains. Node color (red: enriched; blue: depleted) and size correspond to log2 FC. Right: Heatmap detailing the relative abundance of these significant AD tau interactors, presented as z-score–normalized log2 LFQ intensities across AD, PSP, and DLB cases (ANOVA with permutation-based FDR < 5%).
(B) Left: Protein interaction network of significant sarkosyl-soluble DLB tau interactors. Node color (red: enriched; blue: depleted) and size correspond to log2 FC. Right: Heatmap showing the relative abundance of these significant DLB tau interactors across the three disease cohorts.
(C) Functional landscape of the PSP tau interactome. The network map illustrates the broad functional enrichment of the 103 significant proteins either enriched with, or depleted from, sarkosyl-soluble PSP tau.
(D) Left: Sub-network analysis detailing specific functional clusters of significant PSP tau interactors, specifically those involved in signal transduction, autophagy, glial cell development, and glucose metabolism. Node color (red: enriched; blue: depleted) and size correspond to log2 FC. Right: Heatmap displaying the z-score–normalized log2 LFQ intensities of the proteins highlighted in these specific functional sub-networks across the AD, PSP, and DLB fractions.

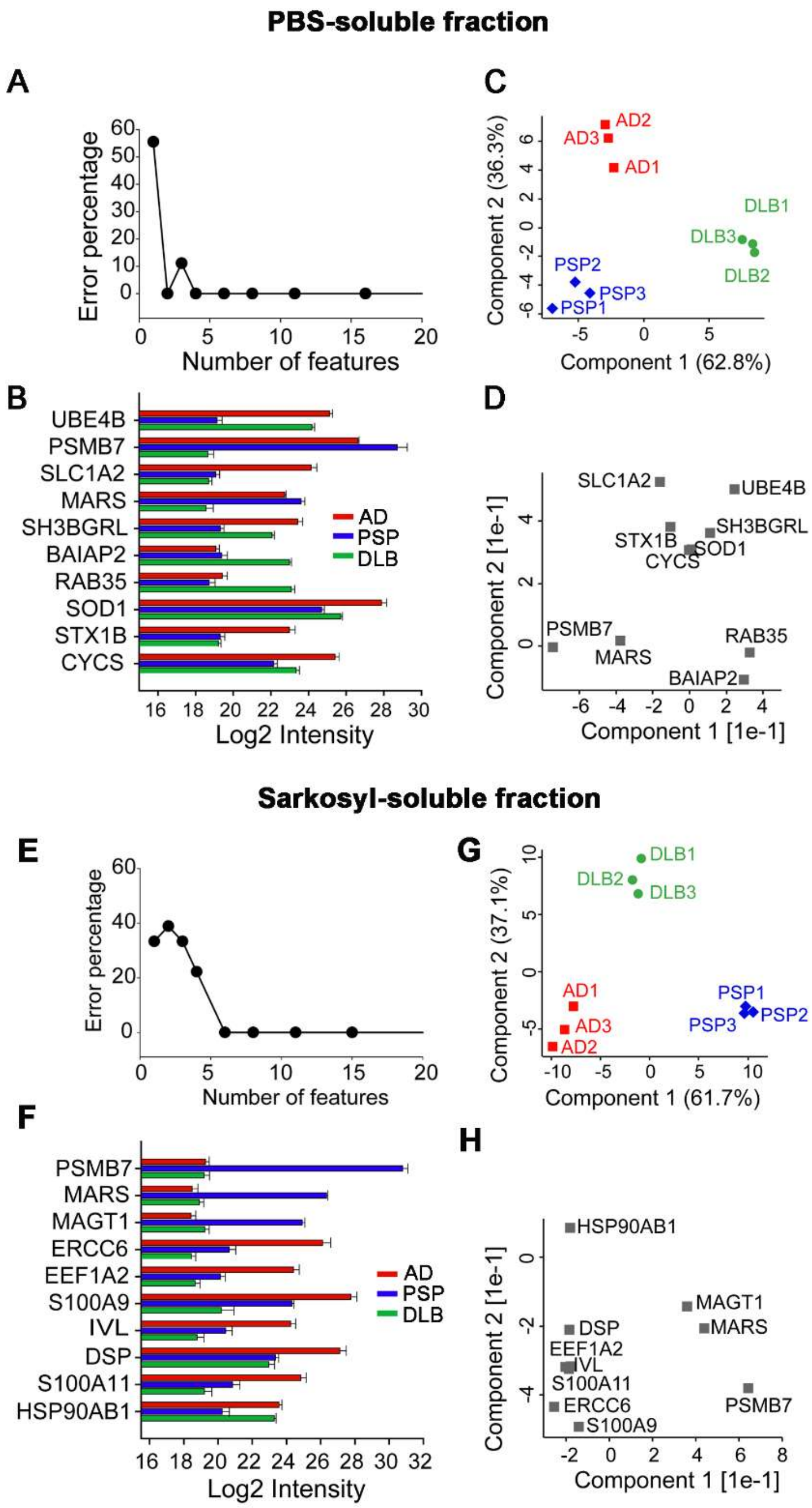

Figure 5. Classification analysis of disease-specific PBS- and sarkosyl-soluble tau interactomes.

(**A, E**) The prediction error percentage corresponding to the number of features used for disease phenotype prediction in (**A**) PBS-soluble or (**E**) sarkosyl-soluble fraction by using the SVM algorithm.

(**B, F**) Abundance (Log2 LFQ intensity) of the ten most informative proteins in (**B**) PBS-soluble or (**F**) sarkosyl-soluble fraction across AD (red), PSP (blue), and DLB (green) tau interactomes.

(**C, G**) PCA analysis using the abundances of these top ten proteins in (**C**) PBS-soluble or (**G**) sarkosyl-soluble fraction across AD (red), PSP (blue), and DLB (green) tau interactomes.

(**D, H**) The PCA loadings of the top ten proteins in (**D**) PBS-soluble or (**H**) sarkosyl-soluble fraction. It illustrates how much each protein influences the variance captured by that component.

Prof. Rakez Kayed
The University of Texas Medical Branch
Neurology
301 University Blvd
Galveston, Texas 77555-1045

21st Jul 2026

Re: EMBOJ-2025-122410R
Disease-specific tau polymorphs define unique protein interaction networks across proteinopathies

Dear Dr. Kayed,

Thank you for submitting your revised manuscript to The EMBO Journal. It has now been re-reviewed by two of the original referees, whose comments are copied below. Both of them find the study substantially strengthened, however referee 1 still retains a few specific concerns that I feel would need to be addressed prior to publication. In particular, the issue regarding overfitting of data from small sample sizes would either require additional analyses or substantive qualifications of statements made in this section. In this light, I am returning the study to you once more with the invitation to address and respond to these remaining points.

When preparing a re-revised manuscript, please also make sure to incorporate the following editorial points at this stage:

- Importantly, our routine image checks for revised manuscripts indicated that the same beta-actin loading controls are shown for the Western blots in Figures 2, 3, and 6, although this is not indicated in either figure legend. Should all the proteins of interest probed for here indeed have been detected on the same, re-probed membrane, this has to be clearly indicated in the legends of these figures. If not, then one loading control specific to each of the blots would have to be provided instead of a generic one. Please explain this in detail.

- Still related to the Western blots in these figures, it is not always easy to identify the band of interest in all of the rather faint cropped blots. It would be good to always indicate with arrows, as has been done for some but not other panels.

- Please carefully go through the reference list and make sure that each reference is complete with citation year, volume, and page/eLocator numbers, information that is currently missing for several of them.

- Please move the information on Funding from the title page into the Acknowledgements section.

- For the Data Availability section, please remove the referee access information but make sure to include a URL to the PRIDE archive in which data have been deposited; and initiate the official release process at this stage. Moreover, the information on BioRender should be relocated into the Methods section.

- The 'Appendix Tables S1-6' would be more appropriate as 'Expanded View Datasets'; hence please rename them into 'Dataset EV1-6' both in the files as well as when referring to them in the manuscript text and elsewhere. Please make sure to include their respective legends in a separate tab of each respective XLSX workbook.

- Finally, please provide suggestions for a short 'blurb' text prefacing and summing up the conceptual aspect of the study in two sentences (max. 250 characters), followed by 3-5 one-sentence 'bullet points' with brief factual statements of key results of the paper; they will form the basis of an editor-written 'Synopsis' accompanying the online version of the article. Please also upload a synopsis image, which can be used as a "visual title" for the synopsis section of your paper. The image (maybe based on the upper right part of Figure 8?) should be ideally in JPG format, and please make sure that it remains in the modest dimensions of exactly 550 pixels wide and 300 pixels high.

I am therefore returning the manuscript to you for a second, final round of revision, with the link below for eventual resubmission. Should you have any questions regarding the referee comments or this decision, please do not hesitate to contact me directly.

Yours sincerely,

Hartmut Vodermaier

Hartmut Vodermaier, PhD
Senior Editor, The EMBO Journal

h.vodermaier@embojournal.org

*** PLEASE NOTE: All revised manuscript are subject to initial checks for completeness and adherence to our formatting guidelines. Revisions may be returned to the authors and delayed in their editorial re-evaluation if they fail to comply to the following requirements. As a first step please read our guidelines for revised submissions: https://link.springer.com/journal/44318/submission-guidelines#cms-Revised-submissions

1) Every manuscript requires a Data Availability section (even if only stating that no deposited datasets are included). Primary datasets or computer code produced in the current study have to be deposited in appropriate public repositories prior to resubmission, and reviewer access details provided in case that public access is not yet allowed.

2) Each figure legend must specify
- size of the scale bars that are mandatory for all micrograph panels
- the statistical test used to generate error bars and P-values
- the type error bars (e.g., S.E.M., S.D.)
- the number (n) and nature (biological or technical replicate) of independent experiments underlying each data point
- Figures may not include error bars for experiments with $n<3$; scatter plots showing individual data points should be used instead.

3) Revised manuscript text (including main tables, and figure legends for main and EV figures) has to be submitted as editable text file (e.g., .docx format). We encourage highlighting of changes (e.g., via text color) for the referees' reference.

4) Each main and each Expanded View (EV) figure should be uploaded as individual production-quality files (preferably in .eps, .tif, .jpg formats). For suggestions on figure preparation/layout, please refer to our Figure Preparation Guidelines: https://media.springernature.com/original/springer-cms/rest/v1/content/27825798/data/v1

5) Point-by-point response letters should include the original referee comments in full together with your detailed responses to them (and to specific editor requests if applicable), and also be uploaded as editable (e.g., .docx) text files.

6) Please complete our Author Checklist, and make sure that information entered into the checklist is also reflected in the manuscript; the checklist will be available to readers as part of the Review Process File.

7) All authors listed as (co-)corresponding need to deposit, in their respective author profiles in our submission system, a unique ORCiD identifier linked to their name. Please see our Guide to Authors for detailed instructions.

8) Please note that supplementary information at EMBO Press has been superseded by the 'Expanded View' for inclusion of additional figures, tables, movies or datasets; with up to five EV Figures being typeset and directly accessible in the HTML version of the article.

9) To facilitate reproducibilty and cross-laboratory adoption of methodologies, please structure the Materials & Methods section as outlined in our guide to authors, including a completed Reagents and Tools Table.

10) Digital image enhancement is acceptable practice, as long as it accurately represents the original data and conforms to community standards. If a figure has been subjected to significant electronic manipulation, this must be clearly noted in the figure legend and/or the 'Materials and Methods' section. The editors reserve the right to request original versions of figures and the original images that were used to assemble the figure. Finally, we generally encourage uploading of numerical as well as gel/blot image source data.

At EMBO Press we ask authors to provide source data for the main manuscript figures. You will receive a separate email with instructions for providing source data with your revised manuscript, including how to upload and organize the files.

In the interest of ensuring the conceptual advance provided by the work, we recommend submitting a revision within 3 months (19th Oct 2026). Please discuss the revision progress ahead of this time with the editor if you require more time to complete the revisions. Use the link below to submit your revision:

Link Not Available

Referee #1:

The revised manuscript is strengthened by the independent PRM-MS validation cohort, and the additional co-IP/western and dot-blot data. The revised Discussion also now describes the study as a correlative resource, distinguishing observations from mechanistic interpretation. Most of my original concerns have been addressed.

However, the title still states that tau polymorphs "define" unique interaction networks. This is stronger than the data support and is inconsistent with the revised limitations paragraph. I would suggest "are associated with" instead of "define".

Also, the overfitting point has not yet been adequately addressed. The authors have validated selected signature proteins in an independent cohort, which is useful, but the machine-learning claim itself remains essentially unchanged. The manuscript still reports 0% SVM prediction error using as few as four to six features from a discovery cohort of only three cases per group. With such a small sample size, perfect separation is not persuasive evidence of a generalizable classifier, particularly if feature selection and model evaluation were performed on the same nine samples. A label-shuffling analysis using the full feature-selection-plus-classification pipeline, together with a clear statement of whether feature selection was performed inside the cross-validation loop, is straightforward to provide and should accompany any retained classification claim. Testing the trained classifier on the independent cohort would be stronger still. If this is not done, the section should be substantially de-emphasised and presented as exploratory feature discovery rather than as evidence for "accurate phenotypic classification".

Referee #2:

The authors have adequately addressed my concerns. The revisions have strengthened the manuscript, and the additional validation increases confidence in the main conclusions. I have no further comments and consider the manuscript suitable for publication in its present form.

Dear Dr. Vodermaier.

We want to thank you and the reviewers for the comments on our manuscripts.

As you suggested, we have revised the manuscript to address the editorial comments. In addition, we have addressed the comment regarding overfitting in the point-by-point response below:

Editor's comments:

- Thank you for submitting your revised manuscript to The EMBO Journal. It has now been re-reviewed by two of the original referees, whose comments are copied below. Both of them find the study substantially strengthened, however referee 1 still retains a few specific concerns that I feel would need to be addressed prior to publication. In particular, the issue regarding overfitting of data from small sample sizes would either require additional analyses or substantive qualifications of statements made in this section. In this light, I am returning the study to you once more with the invitation to address and respond to these remaining points.

Response: Thank you for your positive comments. We have addressed Referee 1's comments by changing the title to: **"**Disease-specific tau polymorphs are associated with unique protein networks across proteinopathies", as well as subheading of the result section to "Distinct acetylation and phosphorylation profiles are associated with disease-specific tau aggregates", and parts of the Discussion. We have also revised the machine Learning Section to de-emphasize and present as exploratory feature discovery.

Referee #1:

The revised manuscript is strengthened by the independent PRM-MS validation cohort, and the additional co-IP/western and dot-blot data. The revised Discussion also now describes the study as a correlative resource, distinguishing observations from mechanistic interpretation. Most of my original concerns have been addressed.

Response: Thank you for the encouragement!

However, the title still states that tau polymorphs "define" unique interaction networks. This is stronger than the data support and is inconsistent with the revised limitations paragraph. I would suggest "are associated with" instead of "define".

Response: Thank you! We have adjusted the title, subheading of the results section, and Discussion to reflect the suggested changes.

Also, the overfitting point has not yet been adequately addressed. The authors have validated selected signature proteins in an independent cohort, which is useful, but the machine-learning claim itself remains essentially unchanged. The manuscript still reports 0% SVM prediction error using as few as four to six features from a discovery cohort of only three cases per group. With such a small sample size, perfect separation is not persuasive evidence of a generalizable classifier, particularly if feature selection and model evaluation were performed on the same nine samples. A label-shuffling analysis using the full feature-selection-plus-classification pipeline, together with a clear statement of whether feature selection was performed inside the cross-validation loop, is straightforward to provide and should accompany any retained classification claim. Testing the trained classifier on the independent cohort would be stronger still. If this is not done, the section should be substantially de-emphasised and presented as exploratory feature discovery rather than as evidence for "accurate phenotypic classification".

Response: Thank you. We have revised the language to describe this analysis as exploratory feature discovery rather than a validated classifier and moved/softened the corresponding claims in the Abstract stating, “As an exploratory feature discovery machine learning classification discriminated against diseases using as few as four to six protein features.” as well in the Results section stating “Machine learning analysis was employed as an exploratory discovery to identify the most discriminative features of tau interactome across disease phenotypes” and “These findings demonstrate that tau interactomes contain robust, disease-specific molecular signatures that enable exploratory feature discovery classification”.

Referee #2:

The authors have adequately addressed my concerns. The revisions have strengthened the manuscript, and the additional validation increases confidence in the main conclusions. I have no further comments and consider the manuscript suitable for publication in its present form.

Response: Thank you very much your encouragement and seeing how our work contributes to the field.

2nd Revision - Editorial Decision 3rd Aug 2026

Prof. Rakez Kayed
The University of Texas Medical Branch
Neurology
301 University Blvd
Galveston, Texas 77555-1045

3rd Aug 2026

Re: EMBOJ-2025-122410R1
Disease-specific tau polymorphs are associated with unique protein networks across proteinopathies

Dear Dr. Kayed,

Thank you for submitting your final revised manuscript for our consideration. I am pleased to inform you that we have now accepted it for publication in The EMBO Journal.

Your manuscript will be processed for publication by EMBO Press. It will be copy edited and you will receive page proofs prior to publication. Please note that you will be contacted by Springer Nature Author Services to complete licensing and payment information.

You may qualify for financial assistance for your publication charges - either via a Springer Nature fully open access agreement or an EMBO initiative. Check your eligibility: https://link.springer.com/journal/44318/how-to-publish-with-us

Should you be planning a Press Release on your article, please notify the production team via this form in order to coordinate publication and release dates: https://forms.cloud.microsoft/e/eH4A8JVP6g

Thank you again for this contribution to The EMBO Journal and congratulations on a successful publication! Please consider us again in the future for your most exciting work.

Yours sincerely,

Hartmut Vodermaier

Hartmut Vodermaier, PhD
Senior Editor, The EMBO Journal
h.vodermaier@embojournal.org

*****
Please note that it is The EMBO Journal policy for the transcript of the editorial process (containing referee reports and your response letters) to be published as an online supplement to each paper. If you should prefer removal of any referee-only figures included in the point-by-point response(s), e.g. because they may still be used for future publication or because they have been reproduced from published work by others, please do let us know immediately via response email.
More information is available here: https://link.springer.com/partners/embo-press/editorial-policies#Peer%20review
*****

EMBO Press Author Checklist

Corresponding Author Name: Rakez Kayed, Yingxin Zhao	
Journal Submitted to: The EMBO Journal	
Manuscript Number: EMBOJ-2025-122410	

USEFUL LINKS FOR COMPLETING THIS FORM
The EMBO Journal - Author Guidelines
EMBO Reports - Author Guidelines
Molecular Systems Biology - Author Guidelines
EMBO Molecular Medicine - Author Guidelines

Reporting Checklist for Life Science Articles (updated January
This checklist is adapted from Materials Design Analysis Reporting (MDAR) Checklist for Authors. MDAR establishes a minimum set of requirements in transparent reporting in the life sciences (see Statement of Task: 10.31222/osf.io/9sm4x). Please follow the journal's guidelines in preparing your
Please note that a copy of this checklist will be published alongside your article.

Abridged guidelines for figures
1. Data
The data shown in figures should satisfy the following conditions:
- the data were obtained and processed according to the field's best practice and are presented to reflect the results of the experiments in an accurate and unbiased manner.
- ideally, figure panels should include only measurements that are directly comparable to each other and obtained with the same assay.
- plots include clearly labeled error bars for independent experiments and sample sizes. Unless justified, error bars should not be shown for technical
- if n<5, the individual data points from each experiment should be plotted. Any statistical test employed should be justified.
- Source Data should be included to report the data underlying figures according to the guidelines set out in the authorship guidelines on Data

2. Captions
Each figure caption should contain the following information, for each panel where they are relevant:
- a specification of the experimental system investigated (eg cell line, species name).
- the assay(s) and method(s) used to carry out the reported observations and measurements.
- an explicit mention of the biological and chemical entity(ies) that are being measured.
- an explicit mention of the biological and chemical entity(ies) that are altered/varied/perturbed in a controlled manner.
- the exact sample size (n) for each experimental group/condition, given as a number, not a range;
- a description of the sample collection allowing the reader to understand whether the samples represent technical or biological replicates (including how many animals, litters, cultures, etc.).
- a statement of how many times the experiment shown was independently replicated in the laboratory.
- definitions of statistical methods and measures:
  - common tests, such as t-test (please specify whether paired vs. unpaired), simple χ2 tests, Wilcoxon and Mann-Whitney tests, can be unambiguously identified by name only, but more complex techniques should be described in the methods section;
  - are tests one-sided or two-sided?
  - are there adjustments for multiple comparisons?
  - exact statistical test results, e.g., P values = x but not P values < x;
  - definition of 'center values' as median or average;
  - definition of error bars as s.d. or s.e.m.

Please complete ALL of the questions below.
Select "Not Applicable" only when the requested information is not relevant for your study.

Materials

Newly Created Materials	Information included in the manuscript?	In which section is the information available? (Reagents and Tools Table, Materials and Methods, Figures, Data Availability Section)
New materials and reagents need to be available; do any restrictions apply?	Not Applicable	

Antibodies	Information included in the manuscript?	In which section is the information available? (Reagents and Tools Table, Materials and Methods, Figures, Data Availability Section)
For **antibodies** provide the following information:<br>- Commercial antibodies: RRID (if possible) or supplier name, catalogue number and or/clone number<br>- Non-commercial: RRID or citation	Yes	Materials and Method

DNA and RNA sequences	Information included in the manuscript?	In which section is the information available? (Reagents and Tools Table, Materials and Methods, Figures, Data Availability Section)
**Short novel DNA or RNA including primers, probes**: provide the sequences.	Not Applicable	

Cell materials	Information included in the manuscript?	In which section is the information available? (Reagents and Tools Table, Materials and Methods, Figures, Data Availability Section)
**Cell lines:** Provide species information, strain. Provide accession number in repository **OR** supplier name, catalog number, clone number, and/**OR** RRID.	Not Applicable	
**Primary cultures:** Provide species, strain, sex of origin, genetic modification status.	Not Applicable	
Report if the cell lines were recently **authenticated** (e.g., by STR profiling) and tested for mycoplasma contamination.	Not Applicable	

Experimental animals	Information included in the manuscript?	In which section is the information available? (Reagents and Tools Table, Materials and Methods, Figures, Data Availability Section)
**Laboratory animals or Model organisms:** Provide species, strain, sex, age, genetic modification status. Provide accession number in repository **OR** supplier name, catalog number, clone number, **OR** RRID.	Not Applicable	
**Animal observed in or captured from the field:** Provide species, sex, and age where possible.	Not Applicable	
Please detail **housing and husbandry conditions**.	Not Applicable	

Plants and microbes	Information included in the manuscript?	In which section is the information available? (Reagents and Tools Table, Materials and Methods, Figures, Data Availability Section)
**Plants:** provide species and strain, ecotype and cultivar where relevant, unique accession number if available, and source (including location for collected wild specimens).	Not Applicable	
**Microbes:** provide species and strain, unique accession number if available, and source.	Not Applicable	

Human research participants	Information included in the manuscript?	In which section is the information available? (Reagents and Tools Table, Materials and Methods, Figures, Data Availability Section)
If collected and within the bounds of privacy constraints report on age, sex and gender or ethnicity for all study participants.	Not Applicable	

Core facilities	Information included in the manuscript?	In which section is the information available? (Reagents and Tools Table, Materials and Methods, Figures, Data Availability Section)
If your work benefited from core facilities, was their service mentioned in the acknowledgments section?	Not Applicable	

Design

Study protocol	Information included in the manuscript?	In which section is the information available? (Reagents and Tools Table, Materials and Methods, Figures, Data Availability Section)
If study protocol has been **pre-registered, provide DOI in the manuscript**. For clinical trials, provide the trial registration number **OR** cite DOI.	Not Applicable	
Report the **clinical trial registration number** (at ClinicalTrials.gov or equivalent), where applicable.	Not Applicable	

Laboratory protocol	Information included in the manuscript?	In which section is the information available? (Reagents and Tools Table, Materials and Methods, Figures, Data Availability Section)
Provide DOI OR other citation details if **external detailed step-by-step protocols** are available.	Not Applicable	

Experimental study design and statistics	Information included in the manuscript?	In which section is the information available? (Reagents and Tools Table, Materials and Methods, Figures, Data Availability Section)
Include a statement about **sample size** estimate even if no statistical methods were used.	Yes	Results, Figure legend
Were any steps taken to minimize the effects of subjective bias when allocating animals/samples to treatment (e.g. **randomization procedure**)? If yes, have they been described?	Not Applicable	
Include a statement about **blinding** even if no blinding was done.	Not Applicable	
Describe **inclusion/exclusion criteria** if samples or animals were excluded from the analysis. Were the criteria pre-established? If sample or data points were omitted from analysis, report if this was due to attrition or intentional exclusion and provide justification.	Not Applicable	
For every figure, are **statistical tests** justified as appropriate? Do the data meet the assumptions of the tests (e.g., normal distribution)? Describe any methods used to assess it. Is there an estimate of variation within each group of data? Is the variance similar between the groups that are being statistically compared?	Yes	Figure legend

Sample definition and in-laboratory replication	Information included in the manuscript?	In which section is the information available? (Reagents and Tools Table, Materials and Methods, Figures, Data Availability Section)
In the figure legends: state number of times the experiment was **replicated** in laboratory.	Yes	Figure Legend
In the figure legends: define whether data describe **technical or biological replicates**.	Yes	Figure Legend

Ethics

Ethics	Information included in the manuscript?	In which section is the information available? (Reagents and Tools Table, Materials and Methods, Figures, Data Availability Section)
Studies involving **human participants**: State details of **authority granting ethics approval** (IRB or equivalent committee(s), provide reference number for approval.	Not Applicable	
Studies involving **human participants**: Include a statement confirming that **informed consent** was obtained from all subjects and that the experiments conformed to the principles set out in the WMA Declaration of Helsinki and the Department of Health and Human Services Belmont Report.	Not Applicable	
Studies involving **human participants:** For publication of **patient photos**, include a statement confirming that consent to publish was obtained.	Not Applicable	
Studies involving experimental **animals**: State details of **authority granting ethics approval** (IRB or equivalent committee(s), provide reference number for approval. Include a statement of compliance with ethical regulations.	Not Applicable	
Studies involving **specimen and field samples:** State if relevant **permits** obtained, provide details of authority approving study; if none were required, explain why.	Not Applicable	

Dual Use Research of Concern (DURC)	Information included in the manuscript?	In which section is the information available? (Reagents and Tools Table, Materials and Methods, Figures, Data Availability Section)
Could your study fall under dual use research restrictions? Please check biosecurity documents and list of **select agents and toxins** (CDC): https://www.selectagents.gov/sat/list.htm	Not Applicable	
If you used a select agent, is the security level of the lab appropriate and reported in the manuscript?	Not Applicable	
If a study is subject to dual use research of concern regulations, is the name of the **authority granting approval and reference number** for the regulatory approval provided in the manuscript?	Not Applicable	

Reporting

The MDAR framework recommends adoption of discipline-specific guidelines, established and endorsed through community initiatives. Journals have their own policy about requiring specific guidelines and recommendations to complement MDAR.

Adherence to community standards	Information included in the manuscript?	In which section is the information available? (Reagents and Tools Table, Materials and Methods, Figures, Data Availability Section)
State if relevant guidelines or checklists (e.g., **ICMJE, MIBBI, ARRIVE, PRISMA**) have been followed or provided.	Not Applicable	
For **tumor marker prognostic studies,** we recommend that you follow the **REMARK** reporting guidelines (see link list at top right). See author guidelines, under 'Reporting Guidelines'. Please confirm you have followed these guidelines.	Not Applicable	
For **phase II and III randomized controlled trials**, please refer to the **CONSORT** flow diagram (see link list at top right) and submit the CONSORT checklist (see link list at top right) with your submission. See author guidelines, under 'Reporting Guidelines'. Please confirm you have submitted this list.	Not Applicable	

Data Availability

Data availability	Information included in the manuscript?	In which section is the information available? (Reagents and Tools Table, Materials and Methods, Figures, Data Availability Section)
Have **primary datasets** been deposited according to the journal's guidelines (see 'Data Deposition' section) and the respective accession numbers provided in the Data Availability Section?	Yes	Data Availability Section
Were **human clinical and genomic datasets** deposited in a public access-controlled repository in accordance to ethical obligations to the patients and to the applicable consent agreement?	Not Applicable	
Are **computational models** that are central and integral to a study available without restrictions in a machine-readable form? Were the relevant accession numbers or links provided?	Not Applicable	
If publicly available data were reused, provide the respective **data citations in the reference list**.	Not Applicable	
