# Supplementary material for: Disease-specific tau polymorphs are associated with unique protein networks across proteinopathies
Source: EMBO J. 2026 Aug 18;45(19):6946–77. doi: 10.1038/s44318-026-00897-8 (PMC13631283; doi:10.1038/s44318-026-00897-8)
Supplement: Supplementary file 3 [file 44318_2026_897_MOESM10_ESM.zip › Figure 3C, 3J-K/README Fiure 3C, 3J-K.docx]

**README**

**Figure 3C:** Data table represents APOE targeted PRM-MS intensity across a new set of Control, AD, PSP, and DLB groups (n=5), with ordinary one-way ANOVA and Tukey’s post-hoc multiple comparison statistical analysis.

**Figure 3J:** Immunoblots of input (left) and IP with T18 (right) materials from additional cases 1-3 of control (con), AD, PSP, and DLB, showing the full (top) and cropped (bottom) of immunoreactivity for PSMB7, and β-actin, respectively.

**Figure 3K**: Confocal image files as high-resolution multichannel max intensity projection (RGB, TIF) of human brain tissues with AD, PSP, and DLB, showing the immunoreactivity for PLA of Tau13 with PSMB7 (red), MAP2 (green), and DAPI (blue), respectively.

Data table (.xlsx) represents PLA analysis of PSMB7 (PLA puncta per ROI) in human brain tissues with AD, PSP, and DLB, with ordinary one-way ANOVA and Tukey’s post-hoc multiple comparison statistical analysis. Raw data was saved as .csv.
