# Supplementary material for: Disease-specific tau polymorphs are associated with unique protein networks across proteinopathies
Source: EMBO J. 2026 Aug 18;45(19):6946–77. doi: 10.1038/s44318-026-00897-8 (PMC13631283; doi:10.1038/s44318-026-00897-8)
Supplement: Supplementary file 4 [file 44318_2026_897_MOESM11_ESM.zip › Figure 6A, D-G/README Figure 6A, D-G.docx]

**README**

**Figure 6A:** Data table represents normalized abundance of significant tau ubiquitination sites in PBS-soluble and sarkosyl-soluble fractions of AD, PSP, and DLB, with ordinary two-way ANOVA and Tukey’s post-hoc multiple comparison statistical analysis.

**Figure 6D, E:** Immunoblots of input (left) and IP with T18 (right) materials from additional cases 1-3 of control (con), AD, PSP, and DLB, showing the full (top) and cropped (bottom) of immunoreactivity for MARCH9, Skp1, UBA52, and β-actin, respectively.

**Figure 6F**: Confocal image files as high-resolution multichannel max intensity projection (RGB, TIF) of human brain tissues with AD, PSP, and DLB, showing the immunoreactivity for PLA of Tau13 with MARCH9, Skp1, or UBA52 (red), MAP2 (green), and DAPI (blue), respectively.

**Figure 6G:** Data table (.xlsx) represents PLA analysis of MARCH9, SKP1, UBA52 (PLA puncta per ROI) in human brain tissues with AD, PSP, and DLB, with ordinary one-way ANOVA and Tukey’s post-hoc multiple comparison statistical analysis. Raw data was saved as .csv.
