# Supplementary material for: Disease-specific tau polymorphs are associated with unique protein networks across proteinopathies
Source: EMBO J. 2026 Aug 18;45(19):6946–77. doi: 10.1038/s44318-026-00897-8 (PMC13631283; doi:10.1038/s44318-026-00897-8)
Supplement: Supplementary file 5 [file 44318_2026_897_MOESM12_ESM.zip › Figure 7B-F/README Figure 7B-F.docx]

**README**

**Figure 7B-E:** Data table represents normalized abundance of significant tau acetylation sites in PBS-soluble and sarkosyl-soluble fractions of AD, PSP, and DLB, with ordinary two-way ANOVA and Tukey’s post-hoc multiple comparison statistical analysis.

**Figure 7F:** Data table represents normalized abundance of significant tau phosphorylation site in PBS-soluble and sarkosyl-soluble fractions of AD, PSP, and DLB, with ordinary two-way ANOVA and Tukey’s post-hoc multiple comparison statistical analysis.
