# Supplementary material for: Disease-specific tau polymorphs are associated with unique protein networks across proteinopathies
Source: EMBO J. 2026 Aug 18;45(19):6946–77. doi: 10.1038/s44318-026-00897-8 (PMC13631283; doi:10.1038/s44318-026-00897-8)
Supplement: Supplementary file 10 [file 44318_2026_897_MOESM8_ESM.zip › Figure 1C-H/README Figure 1C-H.docx]

**README**

Data table represents normalized abundance of the MAPT peptide in PBS-soluble and sarkosyl-soluble fractions, with ordinary one-way ANOVA and Tukey’s post-hoc multiple comparison statistical analysis.
