# Supplementary material for: Disease-specific tau polymorphs are associated with unique protein networks across proteinopathies
Source: EMBO J. 2026 Aug 18;45(19):6946–77. doi: 10.1038/s44318-026-00897-8 (PMC13631283; doi:10.1038/s44318-026-00897-8)
Supplement: Supplementary file 11 [file 44318_2026_897_MOESM9_ESM.zip › Figure 2F-I/README Figure 2F-I.docx]

**README**

**Figure 2F, G:** Immunoblots of input (left) and IP with T18 (right) materials from additional cases 1-3 of control (con), AD, PSP, and DLB, showing the full (top) and cropped (bottom) of immunoreactivity for FABP5, FH, SLC1A2, and β-actin, respectively.

**Figure 2H**: Confocal image files as high-resolution multichannel max intensity projection (RGB, TIF) of human brain tissues with AD, PSP, and DLB, showing the immunoreactivity for PLA of Tau13 with FABP5, FH, or SLC1A2 (red), MAP2 (green), and DAPI (blue), respectively.

**Figure 2I:** Data table (.xlsx) represents PLA analysis of FABP5, FH, and SLC1A2 (PLA puncta per ROI) in human brain tissues with AD, PSP, and DLB, with ordinary one-way ANOVA and Tukey’s post-hoc multiple comparison statistical analysis. Raw data was saved as .csv.
